# SMARCB1 regulates a TFCP2L1-MYC transcriptional switch promoting renal medullary carcinoma transformation and ferroptosis resistance

**DOI:** 10.1038/s41467-023-38472-y

**Published:** 2023-05-26

**Authors:** Bujamin H. Vokshi, Guillaume Davidson, Nassim Tawanaie Pour Sedehi, Alexandra Helleux, Marc Rippinger, Alexandre R. Haller, Justine Gantzer, Jonathan Thouvenin, Philippe Baltzinger, Rachida Bouarich, Valeria Manriquez, Sakina Zaidi, Priya Rao, Pavlos Msaouel, Xiaoping Su, Hervé Lang, Thibault Tricard, Véronique Lindner, Didier Surdez, Jean-Emmanuel Kurtz, Franck Bourdeaut, Nizar M. Tannir, Irwin Davidson, Gabriel G. Malouf

**Affiliations:** 1 Department of Cancer and Functional Genomics, Institute of Genetics and Molecular and Cellular Biology, CNRS/INSERM/UNISTRA, 67400 Illkirch, France; 2 Department of Medical Oncology, Institut de Cancérologie Strasbourg Europe, 67200 Strasbourg, France; 3 INSERM U830, Équipe Labellisée LNCC, Diversity and Plasticity of Childhood Tumors Lab, Institut Curie Research Centre, 75005 Paris, France; 4 Department of Pathology, The University of Texas MD Anderson Cancer Center, Houston, TX 77030 USA; 5 Department of Genitourinary Medical Oncology, The University of Texas MD Anderson Cancer Center, Houston, TX 77030 USA; 6 Department of Bioinformatics and Computational Biology, Division of Quantitative Sciences, The University of Texas MD Anderson Cancer Center, Houston, TX 77030 USA; 7 Department of Urology, CHRU Strasbourg, Strasbourg University, 67000 Strasbourg, France; 8 Department of Pathology, CHRU Strasbourg, Strasbourg University, 67200 Strasbourg, France; 9 Balgrist University Hospital, University of Zurich, Zurich, Switzerland; 10 INSERM, U830, Pediatric Translational Research, PSL Research University, SIREDO Oncology Center, Institut Curie, Paris, France; 11 ‘Équipe Labellisée’ Ligue National contre le Cancer, Paris, France

**Keywords:** Renal cell carcinoma, Cell death, Targeted therapies, Cancer epigenetics

## Abstract

Renal medullary carcinoma (RMC) is an aggressive tumour driven by bi-allelic loss of SMARCB1 and tightly associated with sickle cell trait. However, the cell-of-origin and oncogenic mechanism remain poorly understood. Using single-cell sequencing of human RMC, we defined transformation of thick ascending limb (TAL) cells into an epithelial-mesenchymal gradient of RMC cells associated with loss of renal epithelial transcription factors *TFCP2L1*, *HOXB9* and *MITF* and gain of *MYC* and *NFE2L2*-associated oncogenic and ferroptosis resistance programs. We describe the molecular basis for this transcriptional switch that is reversed by SMARCB1 re-expression repressing the oncogenic and ferroptosis resistance programs leading to ferroptotic cell death. Ferroptosis resistance links TAL cell survival with the high extracellular medullar iron concentrations associated with sickle cell trait, an environment propitious to the mutagenic events associated with RMC development. This unique environment may explain why RMC is the only SMARCB1-deficient tumour arising from epithelial cells, differentiating RMC from rhabdoid tumours arising from neural crest cells.

## Abstract (web summary)

The molecular mechanisms involved in the development of SMARCB1-deficient renal medullary carcinomas (RMCs) remain to be characterised. Here, the authors integrated RMC omics data to show that ferroptosis resistance contributes to transformation of renal thick ascending limb cells into several RMC cell states.

## Introduction

First described in 1995^1^, renal medullary carcinoma (RMC) is a lethal malignant neoplasm arising from the kidney medulla region. Despite its relative rarity, RMC is the third most common renal cancer among young adults^2^. It typically afflicts male patients of African descent with sickle cell trait at a median age of 28 years, yet the association is still poorly understood^3,4^. RMC is highly aggressive with most patients presenting metastatic disease at the time of diagnosis and less than 5% survive longer than 36 months^5,6^. In addition, RMC tumours are resistant to targeted therapies used for other renal cancers and the best available cytotoxic chemotherapy regimens produce a brief objective response in less than 30% of cases^7,8^. Alternative treatments such as anti-angiogenics, EZH2 inhibitors and immunotherapy have been tested with varying success^6^. RMC tumour tissue resembles a high-grade carcinoma exhibiting reticular or cribriform patterns and usually stain positive for VIM, MUC1, pankeratins, PAX8, HIF1α and VEGF^8,9^. RMC are also characterized by a strong desmoplasia, a prominent inflammatory infiltrate as well as the frequent presence of sickled red blood cells^10,11^.

The hallmark of RMC is loss of SMARCB1 expression^12^, a core subunit of the SWItch/Sucrose Non-Fermentable (SWI/SNF) chromatin remodelling complex. Several mechanisms lead to SMARCB1 loss in RMC including deletions, point mutations, inactivating translocations and loss-of-heterozygosity^6^. SMARCB1 loss is also the hallmark of malignant rhabdoid tumours (RTs), atypical teratoid/rhabdoid tumours (ATRTs) and epithelioid sarcomas (ESs). The majority of RTs and RMCs share common features such as their renal location and low mutation burden^6^. We recently characterized the molecular characteristics of RMC identifying frequent chromosome 8q gain associated with a copy-number gain of MYC^6^. SMARCB1 loss activates the MYC pathway resulting in increased DNA replication stress and DNA damage response. RMC are thought to arise from the distal region of the nephron, however evidence is limited to correlation inference using bulk RNA-seq data from 8 nephron biopsies with identified renal cell populations^6,13^. Thus, despite the above pathology and molecular characterization, the cell of RMC origin is as yet not fully defined and the molecular mechanisms involved in oncogenic transformation associated with SMARCB1 loss remain poorly characterized.

To address these issues, we integrated data from single-cell (sc)RNA sequencing of human tumours, multi-region RNA sequencing, bulk transcriptomic data from 2 RMC cohorts, and SMARCB1 gain of function experiments in cellular models. This comprehensive approach revealed how the thick ascending limb (TAL) cells are transformed into RMC through a transcriptional switch involving loss of renal master regulator TFCP2L1 and activation of a MYC and NFE2L2-associated transformation and ferroptosis resistance programs.

## Results

### RMC ontogeny and molecular characterization of tumour cell states

**Fig. 1:**
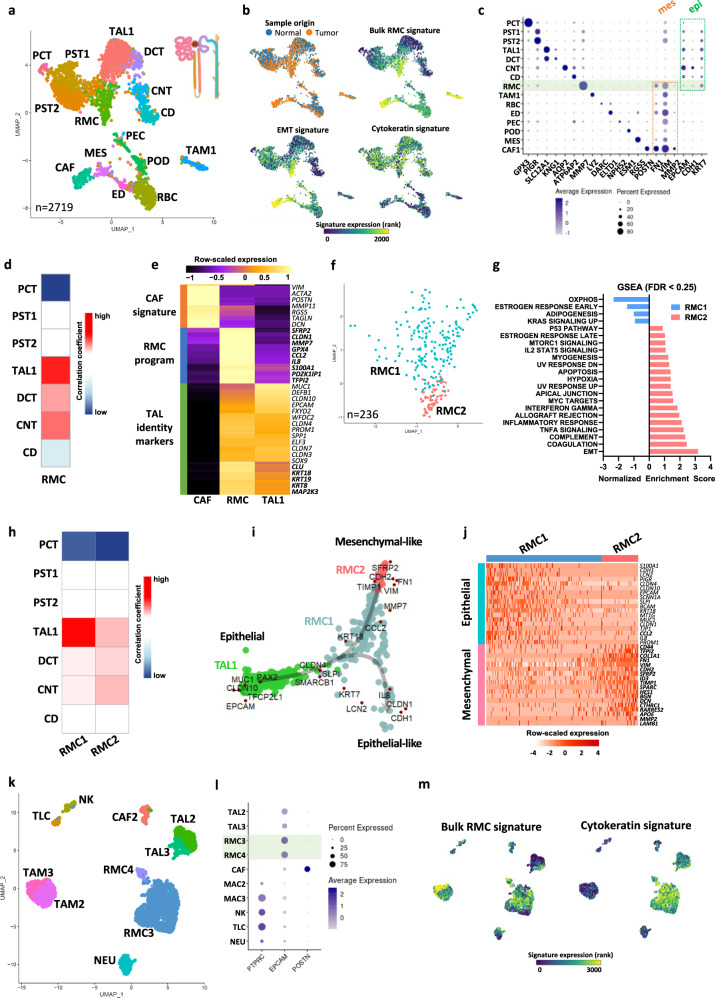
Single-cell RNA sequencing of treated (A-J) and (K-M) naive RMC tumours. **a** UMAP plot of the aggregated treated tumour and normal adjacent tissue (NAT) representing the clusters identified by Seurat using a resolution of 1.12. PCT proximal convoluted tubule cells, PST1/2 proximal straight tubule cells 1 and 2, RMC renal medullary carcinoma cells, TAL1 thick ascending tubule cells of Henle’s loop, DCT distal convoluted tubule cells, CNT connecting tubule cells, CD collecting duct cells, CAF cancer-associated fibroblasts, MES mesangial cells, ED endothelial cells, RBC red blood cells, PEC parietal epithelial cells, POD podocytes, TAM1 tumour-associated macrophages. **b** UMAP projection of sample origin or selected gene signatures. **c** Dot-plots representing gene markers of each identified cluster in the RMC treated sample. Rectangles regroup clusters according to either mesenchymal or epithelial markers. **d** Clustifyr correlation between RMC cells and renal epithelial tubules transcriptomes. **e** Pseudo-bulk heatmap of 100 RMC-specific and 50 CAF-specific genes using CAF1, RMC and TAL1 clusters as a matrix. **f** UMAP representing RMC subclusters as identified by Seurat using a resolution of 1. **g** GSEA showing enriched “Hallmark gene sets” in RMC1 relative to RMC2 cell clusters. **h** Clustifyr correlation between RMC subclusters and renal epithelial tubules transcriptomes**. i** SWNE trajectory analysis of the treated RMC clusters using a set of selected markers per cluster and assuming TAL1 cells as origin. **j** Heatmap representation of a set of selected EMT genes in the 2 RMC subclusters. **k** UMAP plot of the naive tumour cell clusters as identified by Seurat. RMC3/4: Renal medullary carcinoma cells; TAL2/3: thick ascending tubule cells of Henle’s loop; NEU neutrophils, CAF2 cancer-associated fibroblasts, NK natural killers, TLC T-lymphocyte cells, TAM2/3 tumour-associated macrophages. **l** Dot-plots of selected gene markers of immune, epithelial and CAF cells. **m** UMAP projection of the bulk RMC and cytokeratin signatures.

To characterize the molecular features and ontogeny of RMC, we performed scRNA-seq on a post-treatment primary nephrectomy from an RMC patient with lung metastases at diagnosis. The patient showed complete response following 6 cycles of Methotrexate, Vinblastine, Doxorubicin, Cisplatin (MVAC) treatment. A total of 996 cells from the residual tumour site and 1722 cells from normal adjacent renal tissue (NAT) were aggregated and analysed. Seurat UMAP clustering revealed 14 distinct populations amongst which were 7 renal epithelial clusters and 7 stromal and immune clusters (Fig. 1a, b). Epithelial clusters comprised 6 groups of cells from the proximal and distal tubules and 1 group of collecting duct cells each expressing specific markers (Fig. 1c). Amongst these, we identified thick ascending limb (TAL) cells with expression of *SLC12A1*, *EPCAM*, *CDH1* and keratin 7 (*KRT7*), consistent with previous renal scRNA-seq datasets^14–16^.

After merging cancer and NAT samples, we identified populations enriched in the tumour sample comprising TAMs (tumour-associated macrophages) and 2 clusters of cells harbouring an epithelial mesenchymal transition (EMT) signature that we identified to be the RMC tumour and CAF (cancer-associated fibroblast) cells (Fig. 1b). All three clusters expressed specific markers (*LYZ*, *MMP7* and *POSTN*, respectively) with cytokeratin expression in RMC cells (Fig. 1c). Further analyses of RMC and CAFs showed that each expressed overlapping as well as distinct sets of EMT markers (Fig. S1a and Supplementary Data 1a).

The UMAP plot revealed that RMC cells were located close to the TAL population, consistent with a putative cell of origin located in the distal part of the nephron. We interrogated all renal epithelial populations for shared transcriptional signatures with RMC cells and found the best correlation with TAL cells of the kidney medulla (Fig. 1d). Differential gene expression analysis of a pseudo-bulk reconstitution of the RMC versus the CAF populations identified about 150 signature genes for RMC and 50 genes for CAF (Fig. 1e). RMC cells showed a specific oncogenic program, but retained many genes associated with TAL and more broadly epithelial identities (Fig. 1e). RMC and CAF cells did however commonly express EMT genes such as *VIM* and *FN1*, in contrast to TAL cells (Figs. 1c, e). Altogether, these observations identified TAL cells to be the normal renal population most related to RMC and hence the likely cell-of-origin.

To investigate intra-tumoural heterogeneity, we re-clustered the RMC cells identifying distinct RMC1 and RMC2 subpopulations (Fig. 1f). Gene Set Enrichment Analysis (GSEA) revealed that RMC1 were enriched in oxidative phosphorylation (OXPHOS), whereas RMC2 were enriched in EMT, interferon gamma, inflammatory response and hypoxia (Fig. 1g). Correlation of the RMC1 and RMC2 specific signatures to those of normal tubules revealed that RMC1 partly retained a TAL signature that was reduced in RMC2 (Fig. 1h). These observations were independently confirmed by SWNE trajectory analysis that traced transformation of TAL cells to RMC2 via the RMC1 population with some cells retaining a more epithelial identity (Fig. 1i). This was further supported by separation of RMC cells into a ‘stressed’ epithelial-like phenotype with higher levels of cytokines (*IL8*, *LCN2*), keratins and epithelial markers such as *CDH1*, *CLDN1* and into RMC2 cells with higher expression of mesenchymal markers such as *SFRP2*, *CDH2* and *FN1*. Thus, this RMC tumour comprised epithelial-like RMC1 cells and mesenchymal-like RMC2 cells (Fig. 1j).

**Fig. 2:**
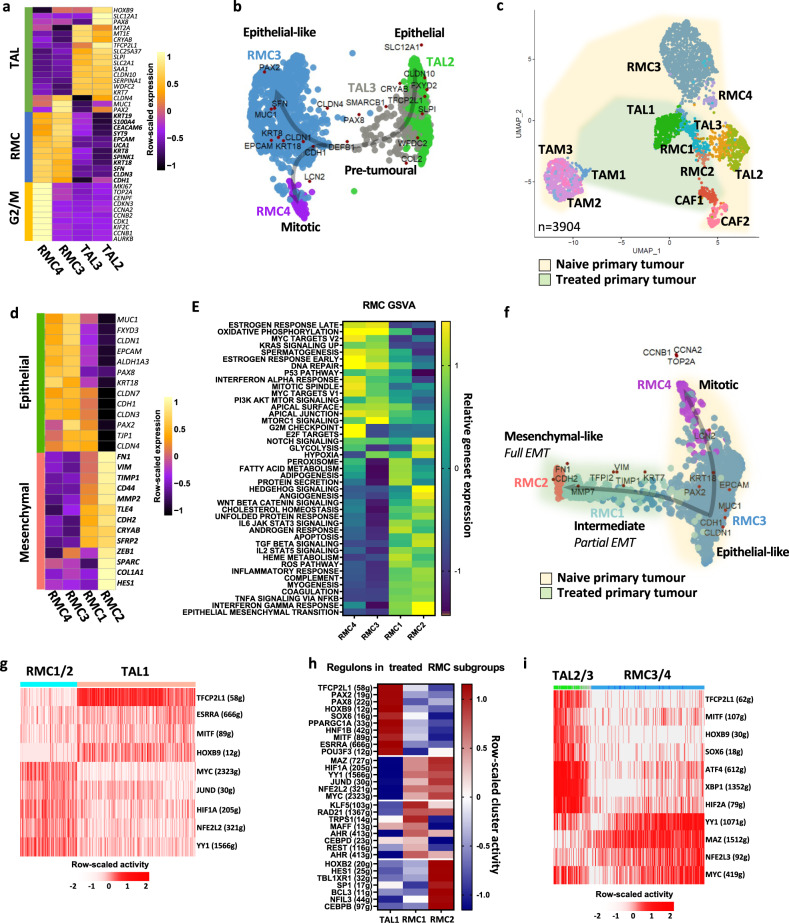
Intra-tumoural heterogeneity of RMC. **a** Pseudo-bulk heatmap of expression of top markers of RMC and TAL clusters. **b** SWNE trajectory analysis of the naive RMC clusters using a set of selected markers per cluster and assuming TAL2 cells as origin. **c** UMAP representing the normalized merge of selected TAL, RMC, CAF and TAM clusters from the treated tumour (green hue) and the naive tumour (yellow hue). **d** Pseudo-bulk heatmap showing a set of known EMT markers in all RMC clusters. Note that as RMC4 were cycling RMC3 cells, they were omitted from the analysis to avoid redundancy. **e** GSVA analysis showing ontologies of indicated RMC clusters. **f** SWNE trajectory analysis of normalized merged RMC clusters from treated and naive tumours using a set of differentially expressed EMT markers. **g** SCENIC analysis of the treated tumour showing top regulons of RMC1/2 and TAL1 cells. Note that in brackets are indicated the number of genes (g) per selected regulon. **h** SCENIC analysis of the treated tumour indicating activities of TAL regulons and RMC1- or RMC2-specific regulons. **i** SCENIC analysis of the naive tumour revealing top regulons of RMC3/4 and TAL2/3 cells.

We next analysed a naive RMC sample from a primary nephrectomy of a 16-year-old patient with regional lymph node and adrenal gland metastases (pT4N1M1) at presentation capturing a total of 3372 cells. Following surgery, the patient showed rapid progressive disease under adjuvant MVAC regimen. The patient was also primary resistant to durvalumab-tremelimumab immunotherapy and EZH2 inhibitor Tazemetostat leading to death within one year of diagnosis. Among 3372 captured cells, a large group of RMC cells was identified along with TAMs and other *CD45*-expressing immune cells (Natural killers, neutrophils and T-cells), *POSTN*-expressing CAFs, and an unexpected population of tumour-associated TAL2/3 cells (Fig. 1k–l). Both the RMC and TAL cells, that segregated into two closely located groups on the UMAP plot, expressed *EPCAM* as well as a cytokeratin signature (Fig. 1l–m). The TAL3 population could be distinguished from TAL2 cells by the lowered expression of the *SLC12A1*, *HOXB9* and *PAX8* renal identity markers (Fig. 2a and Supplementary Data 1b). The RMC3 and RMC4 populations were highly similar with the smaller RMC4 cluster displaying an additional G2/M phase cell cycle signature designating them as mitotic RMC3 cells (Fig. 2a). The SWNE trajectory representation of the TAL and RMC populations illustrated the progressive loss of TAL identity markers from the most differentiated TAL2 to TAL3 with some TAL3 cells closely related to the RMC group that retained an epithelial-like signature (Fig. 2b).

Aggregation of the batch corrected data from the two tumours (Fig. 2c), highlighted differences between the TAL and RMC subpopulations illustrated using a collection of epithelial, mesenchymal, endoplasmic reticulum (ER)-related stress genes (Fig. 2d, Fig. S1b). The RMC3/4 cells from the naive tumour had a marked epithelial character compared to intermediate RMC1 cells from the treated tumour, whereas RMC2 cells had the most mesenchymal phenotype (Fig. 2d). GSVA analyses revealed enrichment for cell cycle in RMC4 cells, OXPHOS and apical junction in RMC3 cells, and EMT and interferon gamma response in RMC1 and RMC2 cells (Fig. 2e). SWNE trajectory analyses highlighted the gradient of epithelial to mesenchymal phenotypes of the different populations (Fig. 2f).

We further performed multi-region tumour RNA-seq on a cohort of four patients, for which single region transcriptome sequencing was previously reported along with that of 7 additional cases (^6^, designated as the MDACC cohort; and Supplementary Data 2). Overall, we generated an additional 25 bulk RNA-seq from multiple regions of these primary tumours and the corresponding regional lymph nodes as well as 3 NATs and analysed intra- and inter-tumour heterogeneity using CIBERSORTx deconvolution to infer their RMC1-3 composition (Fig. S1c, d). For clarity, we did not include the cycling RMC4 signature. Primary tumour sections showed varying composition, some more enriched in the epithelial-like signature, others with epithelial-like and intermediate signatures, and a third group with all 3 signatures. In contrast, the lymph node metastases sections were strongly enriched in the mesenchymal-like signature. These data unravelled intra-tumour heterogeneity in RMC and the importance of tumour cells with a mesenchymal signature to metastatic progression.

We used SCENIC regulon analyses software to identify transcriptional regulatory networks underlying the above signatures^17^. Comparison of the TAL and RMC populations from the treated tumour revealed a transcriptional switch from high *HOXB9* and *TFCP2L1* activity in TAL1 cells, to high *MYC*, *HIF1A*, *YY1* and *NFE2L2* activity in RMC cells (Fig. 2g). These data were consistent with the known role of MYC in RMC transformation, whereas *TFCP2L1* is a previously described determinant of the distal portion of the nephron^18^. Top TAL regulons were progressively lost upon transformation into RMC1 and RMC2 populations exemplified by *TFCP2L1*, *PPARGC1A*, perhaps contributing to the OXPHOS signature^19^, and *HOXB9*, whereas others like *SOX9* were maintained (Fig. 2h).

Comparable observations were made between the TAL and RMC populations of the naive tumour with loss of *TFCP2L1* activity and gain of *MYC* and *NFE2L2/3* activity (Fig. 2i). Interestingly, while TAL2/3 cells displayed *TFCP2L1* activity they also showed a stress signature with prominent activity of *ATF4*, *XBP1* and *HIF2A*. Moreover, they further showed *YY1* and *MYC* activity, hallmarks of RMC cells. TAL1 cells were derived from NAT, whereas TAL2/3 cells were tightly associated with the RMC cells in the tumour sample and showed a stressed pre-tumoural phenotype with activation of several RMC regulons. Each RMC population displayed a characteristic regulon activity such as cell cycle (*BRCA1*, *E2F4/6*) in RMC4 cells^20,21^, epithelial-like (*OVOL2*, *ELF3*) in RMC3 cells^22,23^ and mesenchymal-like (*HES1*, *FOSL2*) in RMC2^24,25^. Notably, activity of the *PAX8* renal identity marker was strongly reduced in the RMC1 and RMC2 populations compared to RMC3 (Fig. S1e).

The role of *TFCP2L1* in driving expression of epithelial genes was reinforced by analyses of the Cancer Cell Line Encyclopaedia (CCLE) showing positive correlation between *TFCP2L1* (and also *OVOL2*) and *EPCAM* (Fig. S1f). Similarly, *TFCP2L1* correlated with epithelia markers and anti-correlated with mesenchymal markers (Fig. S1g). In the TCGA chromophobe renal cell carcinoma dataset, originating also from distal tubules, *TFCP2L1* and *MITF* expression correlated with that of *CDH1* (Fig. S1h).

The above data defined an EMT gradient of RMC cells defined by distinct transcriptional signatures also found in patient tumour samples. NAT-derived TAL1 cells were further distinguished from tumour-associated TAL2/3 cells that displayed a stressed, pre-tumoral phenotype in their transcriptional signatures and regulon activities.

### Tumour cell state of a patient derived RMC xenograft

We analysed a patient derived xenograft (IC-PDX-132) from an RMC tumour treated with 6 cycles of cisplatin, gemcitabine and bevacizumab that had undergone 4 passages of subcutaneous injections on immunocompromised mice. Around 10,000 cells were captured and the sequences aligned to a human-mouse hybrid genome. A large group of human RMC tumour cells were identified with high expression of *EPCAM* and the bulk RMC signature as well as a group of murine cells corresponding to CAFs and pericytes, TAMs and monocytes, and a smaller number of other immune cells (Fig. S2a–c and Supplementary Data 1c). A third group that we tagged ‘LQ’ (low-quality) comprised cells with high levels of mitochondrial genes and potential doublets, that were removed from the subsequent analyses.

Re-clustering the RMC cells revealed 4 subpopulations together with some mouse cells of undefined identity that were not further considered (Fig. S2d). The RMC8 cluster showed a strong cell cycle signature and regulon activity designating them as mitotic RMC cells, whereas RMC6 cells displayed high hypoxia and stress-associated regulons such as *ATF4* and *DDIT3* (Fig S2d–f)^26^. RMC5 and RMC7 on the other hand corresponded to epithelial-like and intermediate state cells respectively analogous to the RMC3 and RMC1 cells in the human tumours (Fig. S2e). No distinct highly mesenchymal population was observed, although the mitotic RMC8 cells showed the most dedifferentiated phenotype and highest expression of *FN1* and *CD44*. SCENIC analyses of these populations identified the key *MYC*, *YY1*, and *NFE2L2* regulons in the RMC cells as seen above in primary human tumours (Fig S2f).

These analyses revealed that the RMC PDX comprised principally epithelial-like, intermediate and mitotic RMC cells as well as a subpopulation of hypoxic cells consistent with the idea that angiogenesis could not fully irrigate the rapidly proliferating tumour.

### Characterization of the RMC microenvironment

In addition to TAL and RMC cells, scRNA-seq revealed prominent CAF and TAM populations in the RMC tumour microenvironment (TME). Analyses of CAFs from both tumours revealed two populations with either a myofibroblast myCAF signature (CAF1) predominant in the treated tumour or an inflamed iCAF signature (CAF2) in the naive tumour (Fig. S3a). Renal CAFs may arise from pericyte-like mesangial cells^27^. SWNE analyses incorporating NAT-derived mesangial (MES) cells supported the idea they gave rise to the two CAF populations.

Analyses of the TAM population identified TAM1 cells displaying a pro-inflammatory M1 signature (Fig. S3b). In contrast, TAM2 and TAM3 displayed an anti-inflammatory M2 signature with high expression of known M2 markers IL10 and MAF^28^, that was strongest in TAM3. SWNE trajectory analysis further confirmed the idea that the TAM2 signature represented an intermediate state between the most polarized TAM1 and TAM3 states.

We then applied the CAF and TAM signatures to the bulk-RNA-seq data from the patient tumour sections as described above. CAF2 cells were detected in all primary and metastases sections, whereas CAF1 were not present in all primary sections and lowly represented in metastases sections (Fig. S3a). Likewise, the TAM2 and TAM3 signatures were detected in a subset of primary and metastases sections, whereas the TAM1 signature was poorly represented in the majority of primary tumour sections, but was highly enriched in the lymph node metastases sections (Fig. S3b).

These analyses showed that the naive tumour and untreated primary patient sections displayed a pro-tumoural, immunosuppressive microenvironment with predominantly iCAFs and M2-type TAMs. However, the MVAC-treated microenvironment was characterized by M1-type TAMs and myCAFs.

### Cultured RMC cells recapitulate the EMT gradient

**Fig. 3:**
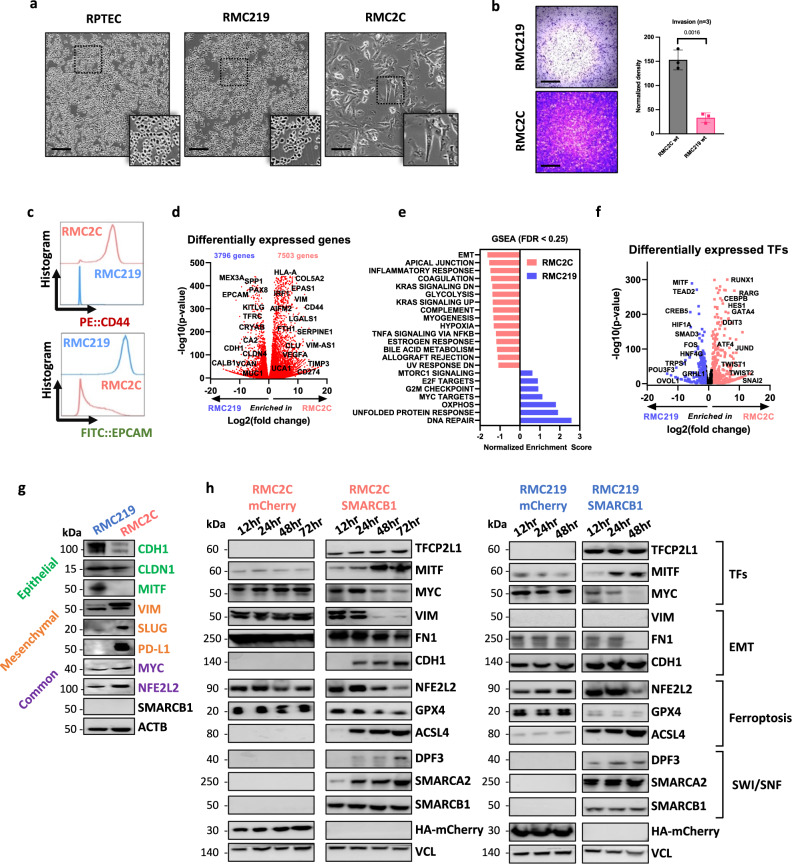
Cultured RMC cells recapitulate EMT cell states. **a** Phase-contrast microscopy at 20X magnification of normal kidney (RPTEC) and tumour cells (RMC219 and RMC2C) showing distinct morphologies of RMC lines. Scale bar: 250 µm. *n* = 3 independent biological replicates. **b**. Brightfield microscopy at ×4 magnification of Boyden chamber matrigel assays using RMC lines (left) and absolute quantification using absorbance of resuspended crystal violet (right). Scale bar: 1000 µm. Biological triplicates are plotted as means ± SD and one-sided unpaired *t* test analyses were calculated by Prism 5; *p* value is indicated. **c** Flow cytometry of membrane protein expression of EPCAM and CD44 in RMC lines. **d** Volcano plot depicting differentially expressed genes using normalized bulk RNA-seq of RMC lines. *P* values were derived using the Wald test and adjusted using Benjamini-Hochberg FDR correction. **e** GSEA using the Hallmarks genesets showing pathways enrichment in respective RMC lines. Note that only pathways with FDR < 0.25 are shown. **f** Volcano plot of differentially expressed 1681 FANTOM5-defined TFs using normalized bulk RNA-seq of RMC lines. *P* values were derived using the Wald test and adjusted using Benjamini-Hochberg FDR correction. **g** Immunoblots detecting the indicated proteins. *n* = 3 independent biological replicates. **h** Immunoblots showing expression of selected proteins upon re-expression of SMARCB1 in RMC2C (left) and RMC219 (right). *n* = 3 independent biological replicates. Source data are provided in the Source Data files.

To better define the mechanism by which SMARCB1 loss drives transition from the TFCP2L1-TAL epithelial program to the MYC-driven oncogenic program, we analysed RMC2C and RMC219 cells^6,29^. RMC219 cells displayed a regular rounded morphology similar to primary RPTEC renal epithelial cells (Fig. 3a). RMC2C cells were larger with a more mesenchymal morphology and were much more invasive than the RMC219 cells (Fig. 3a, b). Similarly, flow cytometry indicated that RMC219 cells were EPCAM high, whereas RMC2C cells were CD44 high (Fig. 3c), a marker of RCC aggressiveness^30^. A similar analysis of the UOK360 and UOK353^31^ lines by flow cytometry revealed intermediate phenotypes. UOK360 displayed higher EPCAM and lower CD44 than UOK353 and more resembled RMC219 cells (Fig. S4a, b). Note however that UOK360 expressed both CD44 and EPCAM discriminating them for the most epithelial RMC219 cells. UOK353 on the other hand, had lower EPCAM, but CD44 levels closer to the RMC2C cells. Moreover, we observed a progressive increase in invasive capacity along the EMT gradient from RMC219-UOK360-UOK353-RMC2C (Fig. S4c). Cultured RMC cells therefore formed an EMT gradient as observed in the scRNA-seq data on the human tumours.

We used RNA-seq to characterize the most epithelial and mesenchymal RMC219 and RMC2C lines identifying an extensive set of differentially expressed genes with preferential expression of epithelial markers in RMC219 cells and mesenchymal markers in RMC2C cells (Fig. 3d). GSEA revealed enrichment of EMT, inflammatory response and hypoxia in RMC2C cells, as seen in the RMC2 tumour population, and enrichment of cell cycle and DNA repair in RMC219 cells (Fig. 3e). Moreover, while OXPHOS was enriched in RMC219, glycolysis was enriched in RMC2C suggesting a metabolic switch upon EMT. *MITF* and *POU3F3*, previously reported determinants of nephron morphogenesis and TAL cell differentiation^32,33^, were preferentially expressed in RMC219 cells, whereas EMT-transcription factors like *TWIST1/2* and *SNAI2* were preferentially expressed in RMC2C cells (Fig. 3f). Immunoblot analyses confirmed higher expression of VIM, and SNAI2 in RMC2C and higher expression of CDH1 and MITF in RMC219 cells (Fig. 3g). Both cell lines however showed expression of NFE2L2 and MYC and lacked SMARCB1. These cell lines therefore reproduced epithelial-like and mesenchymal-like phenotypes analogous to those observed in human tumours.

### SMARCB1 re-expression in RMC cells represses the oncogenic program

We analysed expression of SWI/SNF subunits in RMC2C cells compared to other SMARCB1-deficient cell lines and HEK293T kidney cells. As expected SMARCB1 was absent from all tumour lines (Fig. S5a, b). The catalytic ATPase subunit SMARCA2 (BRM) was absent in all lines except VA-ES-BJ (epithelioid sarcoma), while SMARCA4 (BRG1) was detected in all lines except CHLA-06-ATRT (rhabdoid tumour). RMC2C cells showed the most important changes in SWI/SNF composition with absence of SMARCD3, ARID2 and lowest expression of DPF3, PBRM1, BRD7 and ARID1A. Although the bulk patient RNA-seq data also comprised signal from CAF and TAM cells, RMC-specific reductions in SMARCA2, and DPF3 expression could still be observed (Fig. S5c).

We engineered RMC2C and RMC219 cells to re-express SMARCB1, or mCherry as control, in a doxycycline (Dox)-dependent manner. SMARCB1 was maximally expressed in both RMC cell lines already 12 h after Dox addition (Fig. 3h). SMARCB1 expression in RMC2C cells was comparable to that seen in HEKT cells, seen in almost all cells of the population and was integrated into SWI/SNF and co-precipitated with BRG1 (Fig. S6a–c). The renewed presence of SMARCB1 induced rapid re-expression of SMARCA2, but slower re-expression of DPF3 (Fig. 3h). Similarly, the TAL-associated TFCP2L1, MITF and CDH1 were also induced, whereas MYC, NFE2L2 and EMT markers VIM and FN1 were down-regulated. Each line showed a similar response, but with faster kinetics in the epithelial RMC219 cells where the oncogenic program was more rapidly repressed and the epithelial program faster induced than in RM2C2 cells. SMARCB1 re-expression therefore reversed key transcriptional changes observed during TAL to RMC transformation.

**Fig. 4:**
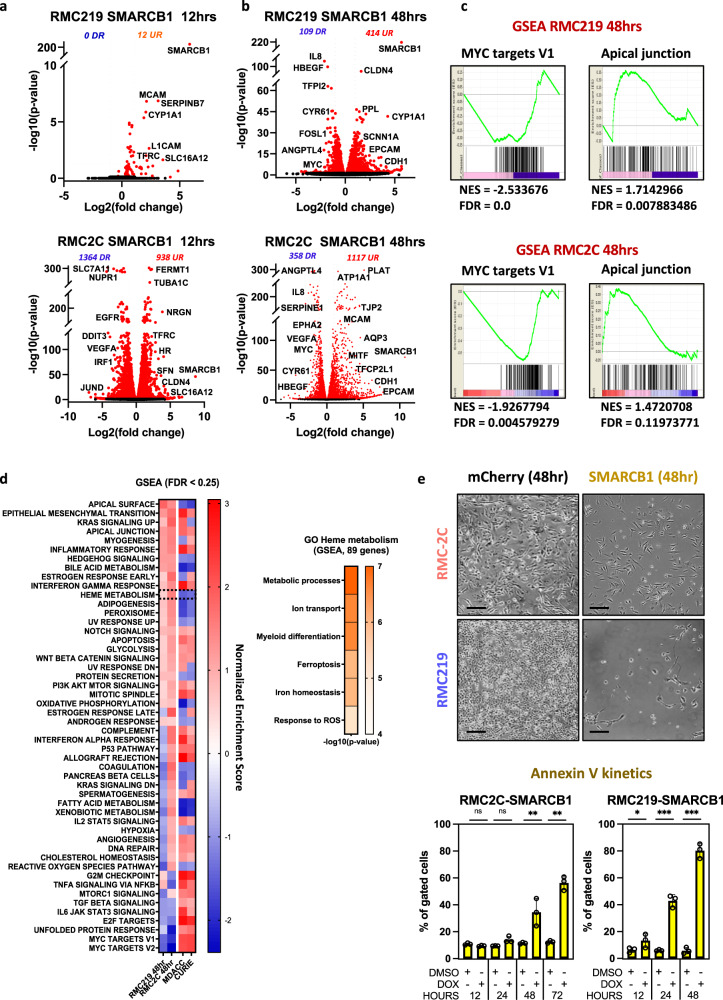
Tumour-suppressor function of SMARCB1. **a** Volcano plot revealing up- and down-regulated genes at 12 h after SMARCB1 re-expression in RMC lines. *P* values were derived using the Wald test and adjusted using Benjamini-Hochberg FDR correction. **b** Volcano plot revealing up- and down-regulated genes at 48 h after SMARCB1 re-expression in RMC lines. *P* values were derived using the Wald test and adjusted using Benjamini-Hochberg FDR correction. **c** GSEA showing top up- and down-regulated pathways upon SMARCB1 re-expression (48 h) with similar ontologies observed in both lines. **d** Integrative heatmap showing GSEA Hallmarks enrichments (left panel) in SMARCB1 re-expressing RMC lines and 2 cohorts of RMC primary tumours (MDACC: *n* = 11; Curie: *n* = 5) and Metascape ontology analysis of genes constituting the GSEA “Heme metabolism” term (right panel). FDR values were derived by GSEA using permutation and Benjamini-Hochberg correction. **e** Phase-contrast microscopy at ×10 magnification of RMC lines 48 h after re-expression of either SMARCB1 or mCHERRY control. Scale bar: 500 µm. (upper panel) Quantification of cell death in RMC lines at selected time-points upon SMARCB1 re-expression, as assessed by flow cytometry (lower panel). Note that the % of cells staining positive for either ANXA5 or propidium iodide were tagged as “dead”. The remaining unstained cells were tagged “viable”. Biological triplicates are plotted as means ± SD and one-sided unpaired *t* test analyses were performed by Prism 5 by comparing matched time-points: *p* values: ns= *p* > 0.05; *=*p* < 0.05; **=*p* < 0.01; ***=*p* < 0.001, RMC2C: *p* values 0.074, 0.082, 0.008 0.00006. RMC219: *p* values 0.046, 0.00008, 0.00001. Source data are provided as a Source Data files.

To globally assess gene expression upon SMARCB1 re-expression, we performed RNA-seq in each cell line 12 and 48 h after Dox-treatment. In RMC2C cells, a rapid transcriptional response was seen with 938 down-regulated and 1364 up-regulated genes after 12 h compared to RMC219 cells where only 12 genes were up-regulated over the same period (Fig. 4a, Supplementary Data 3). After 48 h, a larger number of up and down-regulated genes were observed in both cell lines (Fig. 4b and Fig. S6d). Despite the differences in kinetics and numbers of affected genes, GSEA analyses revealed that in both lines, genes down-regulated were involved in oncogenic functions such as cell cycle and proliferation, designated by the GSEA terms MYC or E2F-targets in agreement with accumulation of G1/S phase RMC2C cells 12 and 48 h after Dox treatment (Fig. S6e). Up-regulated genes were designated by epithelial-like program terms such as cell adhesion, apical junction and apical surface (Fig. 4c). Comparison with bulk-RNA-seq from RMC patients relative to their NAT from both MDACC and Institut Curie cohorts showed the opposite profile with genes up-regulated in the SMARCB1-deficient tumours enriched in proliferation, cell cycle and JAK-STAT3 pathway, whereas those down-regulated associated with apical surface (Fig. 4d). Similarly, while OXPHOS was increased upon SMARCB1 expression in cell lines, it was reduced in RMC tumours. RMC cell lines hence reproduce phenotypes and transcriptional signatures seen in RMC tumours whose key features were reversed by SMARCB1 re-expression.

### SMARCB1 re-expression in RMC cells induces ferroptotic cell death

**Fig. 5:**
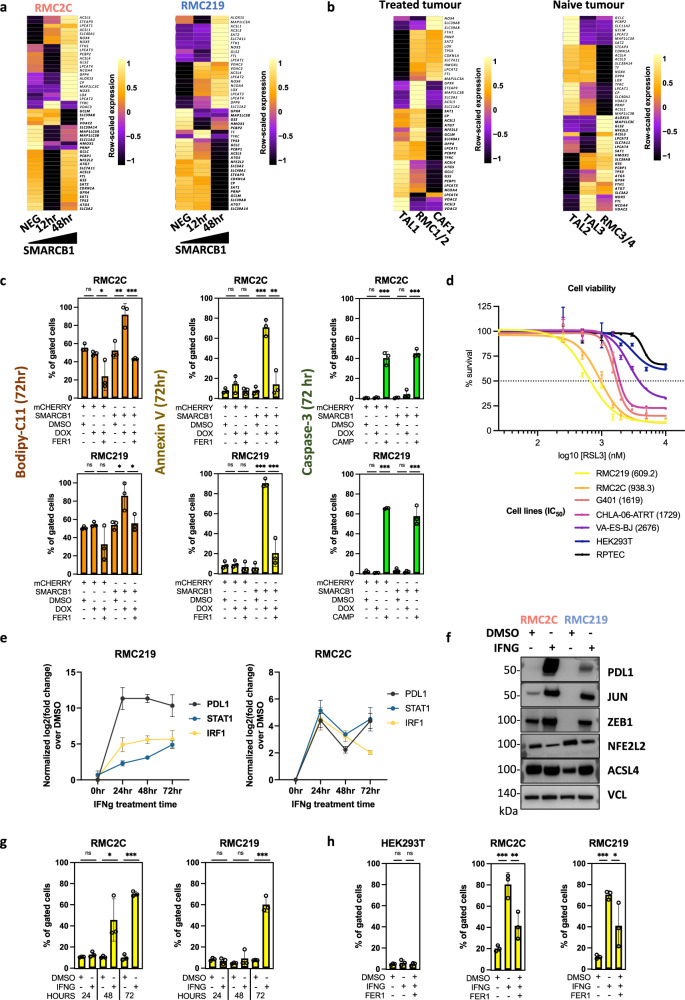
SMARCB1 regulates ferroptosis. **a** Heatmap showing the KEGG ferroptosis gene signature in SMARCB1 re-expressing RMC2C (left) and RMC219 (right) cells. **b** Heatmap showing expression of the ferroptosis gene signature in RMC and TAL clusters. **c** Flow cytometry quantification of Bodipy-C11, ANXA5 and cleaved CASP3 at 72 h in SMARCB1 or mCHERRY expressing cells and using either Ferrostatin-1 (Fer1) or camptothecin (CAMP) as controls. Biological triplicates are plotted as means ± SD and one-sided unpaired *t* test analyses were performed by Prism 5, ns= *p* > 0.05; *=*p* < 0.05; **=*p* < 0.01; ***=*p* < 0.001 and ****=*p* < 0.0001. *P* values: upper left panel: 0.076, 0.027 0.005, 0.001; lower left: 0.05 0.06, 0.01, 0.02; upper centre panel: 0.12, 0.13, 0.0001 0.001; lower centre panel: 0.37, 0.21 0.000007 0.0004; upper right panel: 0.16, 0.0002, 0.09, 0.0001; lower right panel: 0.09, 2.27 E–09, 0.18 0. 0002. **d**. Cell viability (IC50) upon increasing concentrations of RSL3, a class II ferroptosis inducer. Biological triplicates are plotted as means ± SEM. **e** Gene expression changes of known IFNg downstream targets upon treatment of RMC lines with 10 ng/mL recombinant human IFNg. Biological triplicates are plotted as means ± SEM. **f** Immunoblots showing expression of selected EMT and ferroptosis markers in RMC lines treated either with IFNg or DMSO vehicle control. *n* = 3 independent biological replicates. Molecular mass markers in kDa are indicated. **g** Cell death quantified by flow cytometry using annexin-V in RMC lines. Biological triplicates are plotted as means ± SD and one-sided unpaired *t* test analyses were performed by Prism 5, ns=*p* > 0.05; *=*p* < 0.05; **=*p* < 0.01; ***=*p* < 0.001. *P* values: left panel: 0,09 0,01 3,72E-06; right panel: 0.22, 0.17, 0.0001. **h** Flow cytometry-based quantification of cell death at 72 h upon treatment with IFNg alone, IFNg with Fer1 or DMSO in RMC lines and normal kidney cells as control. Represented values are the mean of 3 biological replicates as means ± SD and unpaired *t* test analyses were performed with Prism5 by comparing conditions to matched DMSO. *P* values: ns=*p* > 0.05; *=*p* < 0.05; **=*p* < 0.01; ***=*p* < 0.001 and ****=*p* < 0.0001. *P* values: left panel: 0.21 0.23; centre panel: 0.0004 0.008; right panel 9.98 E–06, 0.037. Source data are provided as a Source Data files.

SMARCB1 re-expression induced cell death with a 10–20-fold increase in the number of Annexin V-expressing cells (Fig. 4e). RMC219 cells responded rapidly with many dead cells detected by 24 h after Dox addition, whereas death of RMC2C cells was evident at 48 h, but required a longer time to reach higher levels (Fig. 4e). To understand the mechanism of cell death, we examined the gene expression changes and noted that Heme metabolism was amongst the pathways strongly up-regulated upon SMARCB1 re-expression and down-regulated in RMC patients (Fig. 4d). The Heme metabolism GSEA term covers iron homoeostasis, response to reactive oxygen species and ferroptosis (Fig. 4d, right panel). Following SMARCB1 re-expression, key anti-ferroptosis genes such as *NFE2L2*, *NUPR1* and their target *GPX4*, a well-characterized inhibitor of lipid peroxidation^34^ were down-regulated in both lines (Figs. 5a and 3h). On the other hand, Transferrin (*TF*) and transferrin receptor (*TFRC*) regulating iron uptake were both rapidly induced in RMC219 and RMC2C cells (Figs. 4a, b, 5a and Fig. S6c). Following these acute events, at 48 h we observed increased expression of a subset of genes involved in lipid peroxidation namely *DPP4*, *LOX*, *LPCAT* paralogs and *ACSL4* (Figs. 5a and 3h). These data suggested that SMARCB1 re-expression induced an acute increase in iron uptake followed by increased lipid peroxidation and ferroptosis.

Complementary observations were made from our scRNA-seq dataset where SCENIC showed that RMC tumour cells were characterized by the activation of the NFE2L2/3 regulon a major regulator of ferroptosis (Figs. 2d, i)^35,36^. Consequently, expression of *NFE2L2*, *GPX4* and other anti-ferroptosis genes was upregulated in RMC cells from the MVAC-treated tumour compared to TAL cells, whereas many pro-ferroptosis genes were higher expressed in TAL cells (Fig. 5b). Similarly, in the naive tumour, *GPX4* and anti-ferroptosis genes were upregulated in RMC compared to TAL cells (Fig. 5b). RMC tumours further showed staining with 4-Hydroxynonenal (4-HNE) antibodies compared to the surrounding stromal cells confirming their propensity to undergo lipid peroxidation (Fig. S7a). Moreover, in agreement with their pre-tumoural phenotype, the RMC-associated TAL3 cells showed up-regulated expression of anti-ferroptosis genes and down-regulated expression of the pro-ferroptosis genes compared to the TAL2 cells. Activation of the *MYC* and *NFE2L2/3* regulons in these cells was therefore accompanied by activation of the ferroptosis resistance program.

We next assessed if SMARCB1 re-expression and increased expression of the lipid peroxidation genes translated into an elevation of lipid ROS assessed using BODIPY-C11-based flow cytometry (Fig. 5c and Fig. S8). SMARCB1 re-expression induced a strong increase of lipid ROS in both lines not seen in mCherry control lines. High lipid ROS was associated with increased AnnexinV-positive cells. Importantly, the increase in lipid ROS and in Annexin-V positive cells were both impaired by ferrostatin-1, a selective ferroptosis inhibitor (Fig. 5c). In contrast, SMARCB1 expression did not induce the activated Caspase 3 apoptosis marker unlike Campothecin treatment. To further confirm ferroptotic cell death, we treated RMC cells with the GPX4 inhibitor RSL3. The RMC cells had IC50 values 2–4 times lower than other RT cell lines and more than 100-fold lower than control RPTEC or HEKT cells (Fig. 5d). We additionally assessed the ability of the pan-caspase inhibitor zVAD-fmk or the necroptosis inhibitor necrostatin-1 (nec1) to inhibit SMARCB1 or RSL3-induced cell death. Reduced cell viability after Dox-induced SMARCB1 expression was rescued by ferrostatin-1 and by nec1, consistent with the previously reported ability of higher concentrations of nec1 to rescue ferroptosis in other tumour cell lines^37,38^, but not by zVAD-fmk (Fig. S7b). Similarly, cell viability in presence of RSL3 was also rescued by ferrostatin-1 and Nec1, but not zVAD-fmk (Fig. S7b). Flow cytometry confirmed that RSL3-induced cell death was rescued by high but not low concentrations of Nec1 (Fig. S7c). As ferristatin does not rescue other forms of death^34^, these data support the observation that SMARCB1 expression induced ferroptotic cell death. Moreover, further evidence for ferroptosis came from immunofluorescence (Fig. S6c) showing not only that TFRC was rapidly induced by SMARCB1 re-expression, but also that while it was located in the cytoplasm in most RMC2C2 cells at 24 h, there were already some small rounded dying cells where TFRC was relocated to the plasma membrane, hallmarks of ferroptosis^39^. TFRC located to the plasma membrane in almost all RMC219 cells at the same stage consistent with the observation that these cells undergo very rapid ferroptosis. These results confirmed that RMC cells were highly sensitive to GPX4 inhibition and that cell death was due to ferroptosis.

IFNg, secreted by the immune microenvironment in tumours in situ, induces tumour cell dedifferentiation and ferroptotic cell death in melanoma^40,41^. IFNg treatment of RMC219 and RMC2C resulted in durable expression of PDL1, STAT1 and IRF1 and of mesenchymal markers JUN and ZEB1, induced in RMC219 cells and up-regulated in RMC2C cells (Fig. 5e, f). In contrast, NFE2L2 expression was reduced. IFNg treatment induced death of RMC2C cells between 48 and 72 h, whereas death of RMC219 cells required 72 h (Fig. 5g). Importantly, treatment with ferrostatin 1 diminished the IFNg-induced cell death showing that it involved ferroptosis (Fig. 5h), while as control no induced cell death was seen with HEK293T.

These results revealed that TAL cells were characterized by a ferroptosis sensitivity program that was progressively replaced in pre-tumoural TAL3 cells, in the RMC tumour populations and in RMC cell lines by a NFE2L2 and GPX4-high ferroptosis resistance program. This process was reversed by SMARCB1 re-expression that down-regulated NFE2L2 and GPX4 or by IFNg treatment leading to cell death by ferroptosis.

### SMARCB1 re-expression promotes genomic SWI/SNF re-localization to enhancers with TFCP2L1 motifs

To investigate the consequences of SMARCB1 re-expression on SWI/SNF localization and the epigenome of RMC2C cells, we performed BRG1 and H3K27ac ChIP-seq 48 h after Dox treatment of SMARCB1 or control mCherry expressing cells. RMC219 cells could not be used due to the rapid cell death upon SMARCB1 expression. SMARCB1 re-expression increased the overall number of H3K27ac peaks, but had little impact on their relative genomic distribution with similar fractions of sites at transcription start sites (TSS) and other genomic regions (Fig. S9a, b). However, comparison of read density at more than 46000 non-redundant H3K27ac sites revealed a gain of sites located distal to the TSS following SMARCB1 re-expression (cluster G2, Fig. S9c), whereas only a minor change was seen at the TSS. A fraction of gained peaks were extended regions reminiscent of super-enhancers (SE) known to regulate genes involved in critical aspects of lineage identity or oncogenic transformation^42,43^. While a large number of H3K27ac-marked SEs and their associated genes were shared between the mCherry and SMARCB1 expressing cells, 240 SE-associated genes were specific to the mCherry line and associated with a variety of functions notably DNA repair and cell cycle (Fig. S9f). More strikingly, 330 SE-associated genes specific to SMARCB1 expressing cells were associated with kidney epithelium development and differentiation as well as cell polarity and junction (Fig. S9g).

**Fig. 6:**
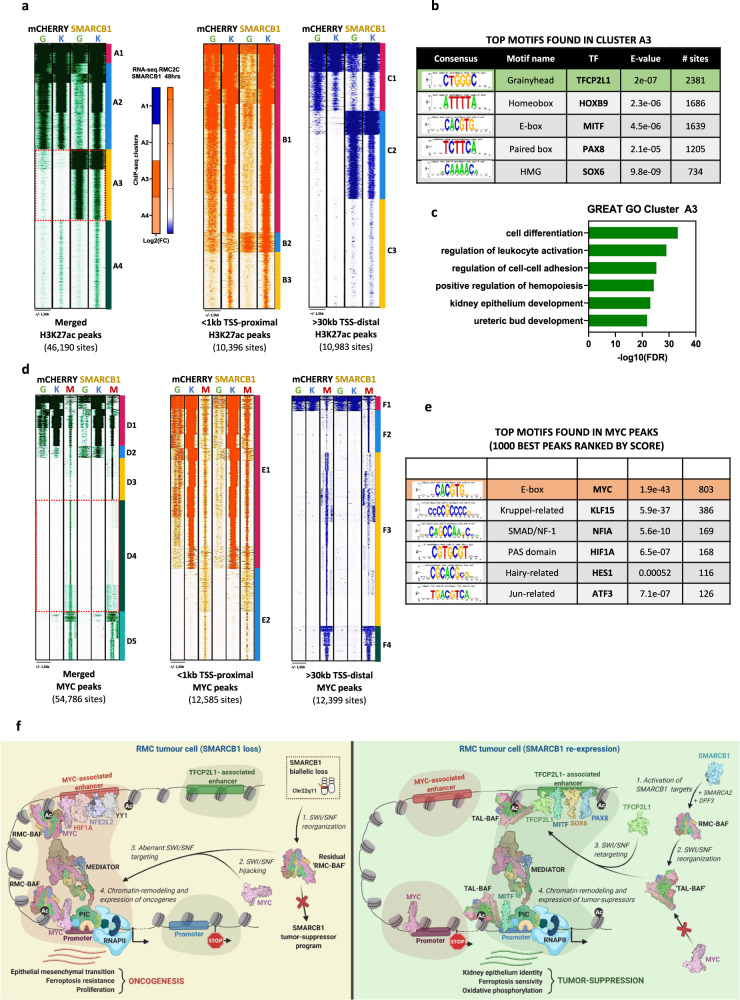
SMARCB1 retargets SWI/SNF complexes to enhancers bearing TFCP2L1 motifs. **a** Read density maps showing genome localization BRG1 (G) and H3K27ac (K) in RMC2C cells expressing either SMARCB1 or mCHERRY using as a reference all merged H3K27ac sites (1st panel), all TSS-proximal H3K27ac sites (3rd panel) and all TSS-distal H3K27ac sites (4th panel). Expression changes for genes associated with BRG1/H3K27ac- clusters following SMARCB1 re-expression are shown in the 2nd panel. **b** RSAT-based motif enrichment analysis using A3 sites ranked by number of sites. **c** Ontology analysis of genes associated with A3 as annotated by GREAT. **d** Read density maps showing genome localization of BRG1 (G), H3K27ac (K) and MYC (M) in RMC2C cells expressing either SMARCB1 or mCHERRY using as a reference all merged MYC sites (1st panel), all TSS-proximal MYC sites (2nd panel) and all TSS-distal MYC sites (3rd panel). **e** RSAT-based motif enrichment analysis using one thousand best MYC peaks ranked (by peak score). **f** Working model of the oncogenic and SMARCB1 tumour-suppressor events in RMC. Created with BioRender.com.

SMARCB1 re-expression also modified BRG1 genomic occupancy with a loss mainly at the TSS (H4, Fig. S9d), but a gain at distal sites (H8, Fig. S9d). Integration of BRG1 and H3K27ac read density profiles at more than 40,000 non-redundant co-occupied sites identified those with concomitant gain of H3K27ac and BRG1 following SMARCB1 re-expression (A3, Fig. 6a) predominantly located distal to the TSS (C2). In contrast, cluster A2 defined sites with reduced BRG1 predominantly located at the TSS (A2/B1) with a smaller set at distal sites (C1). Correlation with RNA-seq data indicated that genes associated with cluster A3/C2 showed increased expression following SMARCB1 re-expression (Fig. 6a). RSAT analyses revealed a strong enrichment for TFCP2L1, HOXB9, and MITF binding motifs at the distal gained A2/C3 sites (Fig. 6b). Moreover, ontology analyses of the nearest genes to the A3/C3 sites showed enrichment in differentiation, cell adhesion and kidney epithelium development (Fig. 6c). SMARCB1-dependent BRG1 recruitment and H3K27ac modification was exemplified at intragenic regulatory elements at the MITF locus, at a putative regulatory element at the 3’ end of the TFCP2L1 gene, or at intragenic regulatory regions of the SYT13 and DOCK1 genes, that were all re-activated following SMARCB1 re-expression (Fig. S9h). SMARCB1 re-expression therefore led not only to re-expression of TFCP2L1 and MITF, but also re-localization of BRG1 to putative H3K27ac marked distal enhancers and super-enhancers associated with the epithelial gene expression program.

We additionally performed Cut&Tag experiments to profile BRG1 and SMARCB1 genomic localization 24 h after Dox treatment. While no SMARCB1 signal was seen in control mCherry cells, strong SMARCB1 occupancy was seen following its Dox-induced expression (Fig. S10a). At a subset of sites, low BRG1 binding and H3K27ac was seen in absence of SMARCB1 (cluster A1), whereas at the remainder BRG1 and H3K27ac were seen only in presence of SMARCB1 (cluster A2). When SMARCB1 occupancy was examined at the 10983 distal sites observed at 48 h (Fig. 6a), de novo recruitment of SMARCB1, BRG1 and marking by H3K27ac was observed at 24 h (Fig. S10b, clusters B2 and B3). Rapid de novo SMARCB1 recruitment along with BRG1 and H3K27ac was exemplified at the MITF, TFCP2L1, SYT13 and DOCK 1 loci as shown above (Fig. S9h). Moreover, in accordance with the strong enrichment for TFCP2L1 binding motifs at these sites (Fig. 6b), TFCP2L1 co-precipitated with BRG1 from extracts of Dox-treated cells (Fig. S10c). Together these results showed that upon its re-expression, SMARCB1 integrated the SWI/SNF complex that interacted with TFCP2L1 and was rapidly recruited to the H3K27ac-marked regulatory elements associated with epithelial genes.

As mentioned above, TFRC was rapidly induced 12 h after SMARCB1 re-expression. The *TFRC* promoter was strongly marked by H2K27ac in both the mCherry control and 24 h after SMARCB1 re-expression (Fig. S10d). Moreover, BRG1 and SMARCB1 were recruited at 24 h. TFRC therefore behaved as an ‘immediate-early’ gene whose promoter was pre-marked with H3K27ac, but whose activation was associated with rapid BRG1 and SMARCB1 recruitment. This contrasts with epithelial program genes whose activation was slower and where both BRG1/SMARCB1 recruitment and H3K27ac modification occurred de novo.

### SMARCB1 re-expression remodels MYC genomic binding

It has been reported that SMARCB1 interacts directly with MYC to antagonize its DNA binding and genomic occupancy in RT cells^44,45^. To address this in RMC cells, we performed MYC ChIP-seq in SMARCB1-expressing and mCherry control cells 48 h after Dox addition. We identified 54,786 non-redundant MYC sites, a much larger number than previously observed^44^. All MYC-bound sites in G401 RT cells, that were predominantly located close to the TSS, were occupied also in RMC2C cells (Fig. S11a, b). For example, MYC sites commonly bound in G401, RMC2C2 and in the Hela ENCODE data sets were observed at the *NCL* and *CDK4* loci (Fig. S11b).

In keeping with reduced MYC expression, around 50% fewer peaks were observed in SMARCB1 expressing cells where its occupancy was remodelled with a relative re-localization to the TSS that increased from 24 to 41% of the detected peaks (Fig. S9a, b). Read density profiles at the non-redundant MYC sites identified those with gained (I2/I8, Fig. S9e) or diminished (I3/I9) occupancy located at both TSS proximal and distal regions. Notably, integration with BRG1 and H3K27ac datasets revealed that MYC occupancy was increased at TSS proximal sites marked by H3K27ac, but characterized by diminished BRG1 occupancy (D1/E1, Fig. 6d). In contrast, a large set of distal located sites were lost upon SMARCB1 re-expression (D4/F3) with a smaller number showing increased occupancy (D3/F2). Global analyses confirmed that BRG1 flanking a subset of MYC bound sites in the mCherry control cells was diminished following SMARCB1 re-expression, whereas H3K27ac was unchanged (Fig. S9c). RSAT analysis of the top 1000 MYC peaks confirmed a strong enrichment of the cognate E-box motif (Fig. 6e). MYC-binding motifs were also strongly enriched at the D1-D4 sub-clusters, together with MAZ at D1 sites, whose activity was associated with TAL transformation (Fig. 2g–i and S11d).

As shown above, the term ‘MYC targets’ was a prominent hallmark of genes down-regulated by SMARCB1 re-expression. We determined the % of genes in the GSEA hallmark gene sets overlapping with those associated with each MYC sub-cluster. Genes associated with D1 sites were strongly enriched in MYC targets, mitotic spindle, mTOR, E2F. DNA repair and G2M hallmark signatures (Fig. S12a). Genes associated with D4 also displayed a similar, yet lower, enrichment in many of these pathways. Correspondingly, genes associated with D1 and D4 showed global down-regulation (Fig. S12b), whereas those associated with D2 and D3 showed up-regulated expression. Thus, many genes associated with oncogenic transformation and down-regulated by SMARCB1 re-expression were associated with a gain of promoter-proximal MYC, but strongly reduced BRG1 binding.

A similar analysis of BRG1 sub-clusters, showed genes associated with A2 were strongly enriched in the above oncogenic-associated hallmarks (Fig. S12c). In contrast, A3 sites with strongly gained BRG1 binding were enriched in genes associated with apical junction/surface and kidney morphogenesis hallmarks, consistent with re-activation of an epithelium program. We used ROSE to identify MYC-H3K27ac-marked or BRG1-H3K27ac-marked SEs in control and SMARCB1-expressing cells (Fig. S12d, e). The ontology of the SE-associated genes was consistent with a switch from MYC/BRG1 driving proliferation and oncogenesis in absence of SMARCB1 to TFCP2L1/BRG1 driving an epithelium program in presence of SMARCB1.

To better understand the paradoxical observation that MYC binding increases at down-regulated oncogenic genes, we looked more closely at the large set of diminished D4 sites associated with similar ontology terms to D1. Re-clustering of D4 identified a small number (J1, Fig. S12f) of promoter-proximal sites associated with H3K27ac and a large majority of distal sites (J2, Fig. S12f). Strikingly, a large number of genes were commonly associated with both clusters (Fig. S12g). Hence many genes of the oncogenic program had both promoter-proximal and distal MYC sites showing increased and decreased occupancy, respectively. Importantly, the D4 sites were enriched in binding motifs for HIF1A and SNAI1 in agreement with coordinate activation of MYC, HIF1A and EMT programs in RMC. Loss of MYC at the D4 sites upon SMARCB1 expression was therefore consistent with their role in driving transformation.

Overall, these results showed that SMARCB1 re-expression did not repress MYC genomic occupancy, but rather remodelled its binding profile in a manner suggesting that altered enhancer-promoter communications and loss of promoter-proximal BRG1 binding underlie reduced expression of the proliferation/oncogenic program.

## Discussion

### Oncogenic transformation of TAL cells into RMC

Here we integrate transcriptomic data from RMC patients with gain and loss of SMARCB1 function in cell-based models to decipher the mechanism of a transcriptional switch driving oncogenic transformation and ferroptosis resistance of TAL epithelial cells.

ScRNA-seq analyses of RMC cells compared to NAT identified TAL cells as RMC cell-of-origin. TAL cells were marked by strong activity of *TFCP2L1*, *HOXB9* and *MITF* transcription factors associated with the epithelial expression program. TAL transformation was characterized by loss of expression and activity of these factors, but gain of *MYC* and *NFE2L2* that drive proliferation and ferroptosis resistance. Further evidence for this series of oncogenic events came from the fortuitous capture of tumour-associated TAL2/3 cells that displayed a pre-transformed state retaining *TFCP2L1* activity, while at the same time showing *MYC* and *YY1* activity accompanied by a hypoxia and stress signature.

TAL transformation generated an epithelial-mesenchymal gradient of RMC tumour cells that was reproduced by RMC219, UOK360, UOK353 and RMC2C cell lines. Mesenchymal-like cells were observed in the treated tumour and the mesenchymal transcriptional signature was present in primary tumours from naive patients and was predominant in the lymph nodes. Thus, de-differentiation into this mesenchymal state is not specific to drug-treated tumours, but appears to be an intrinsic feature of RMC tumours that likely contributes to their metastatic spread.

SMARCB1 re-expression in RMC2C cells provided experimental mechanistic support for the above model of TAL-RMC transformation. SMARCB1 expression reactivated *TFCP2L1*, *HOXB9* and *MITF* expression and promoted BRG1 re-localization to enhancers and super-enhancers driving expression of an epithelial expression program that were de novo marked by H3K27ac and enriched in binding motifs for these factors (Fig. 6f). The lack of ChIP-grade TFCP2L1 and MITF antibodies did not allow us to directly confirm their presence at these enhancers. However, we previously showed that MITF interacts with SWI/SNF and actively recruits BRG1 to melanoma-cell promoters and enhancers^46^ and here we showed that TFCP2L1 also co-precipitated with SWI/SNF. In contrast, SMARCB1 re-expression led to reduced levels of MYC and NFE2L2. Genomic profiling revealed a remodelling of MYC genomic binding with sites showing both gained or reduced occupancy. Paradoxically, while MYC binding increased at the proximal promoters of genes involved in oncogenesis, it was lost at sites distal to these genes. Although there are clear limitations in assigning distal binding sites to regulation of a given gene, a large set of genes showed increased MYC binding at the promoter and diminished binding at distal sites suggesting the importance of enhancer-promoter communication in their activation. More importantly however, BRG1 occupancy was strongly reduced at these promoters showing that MYC cooperated with SWI/SNF lacking SMARCB1 to activate the oncogenic program and that BRG1 eviction and not MYC loss reversed the oncogenic process.

Integrating patient and in cellulo-derived data converged to show that pre-tumoral TAL2/3 cells displayed a hypoxia/stress state activating *MYC* and *NFE2L2* to drive ferroptosis resistance allowing survival under conditions favourable to SMARCB1 loss (Fig. 6f). Subsequently, SMARCB1 loss led to BRG1 recruitment at promoters of MYC occupied oncogenic genes and inhibition of the *TFCP2L1/HOXB9/MITF*-driven TAL epithelial program. In RMC cells, SWI/SNF lacking SMARCB1 cooperates with MYC to drive the oncogenic program, whereas SMARCB1-containing SWI/SNF is evicted from MYC-driven oncogenic promoters and re-located to enhancers driving the TAL epithelial program.

### Distinct cells-of-origin and oncogenic mechanisms in RMC and RT

The above observations highlight major differences with previous studies on RT cells. In G401 RT cells, SMARCB1 antagonized MYC DNA binding and chromatin occupancy^44^. In contrast, in RMC cells, antagonism translated not as a loss of MYC binding, but eviction of SMARCB1-containing SWI/SNF from MYC occupied promoters and reduced oncogenic gene expression. It has been reported that BRD9-containing non-canonical (nc)BAF plays a critical role in driving the oncogenic state in SMARCB1-deficient RT^47,48^. NcBAF strongly colocalized with CTCF although other enriched transcription factor motifs were also identified. However, MYC was not amongst the strongly enriched motifs in either study. This leads to the paradoxical observation that despite the essential role of BRD9/ncBAF in driving the transformed state, it is not enriched at MYC-bound sites, whereas depletion studies in RT cells^44^ and our current data revealed MYC as the essential oncogenic driver. Given the strong association of ncBAF with CTCF and not MYC, it is unlikely that the BRG1 seen at the MYC promoters in RMC cells corresponds to ncBAF. Moreover, CTCF motifs were not enriched at the promoter sites where BRG1 was evicted, but were present at distal sites, where no BRG1 was detected. Thus, the role of ncBAF in RMC remains to be determined.

In RT cells, SMARCB1 re-expression led to SWI/SNF re-localization to what have been described as lineage-specific enhancers^49,50^. However, the transcription factor motifs at SMARCB1-bound enhancers were not always informative as to the nature of the cell of origin. The lack of a clearly defined cell(s) of origin, and their intrinsic biology has hampered a detailed understanding of the transformation process. Mechanistic studies were often limited to SMARCB1 re-expression in RT cell lines with little supporting patient data. An exception is atypical teratoid RT (AT/RT) where the epigenetic profiles of the AT/RT tumours were compared with other types of brain tumours or normal brain^51^. Nevertheless, the validity of these comparisons is limited since more recent data provide strong evidence that RT arise following arrest of neural crest cell differentiation into mesenchyme, in particular Schwann cells^52^. Many of the above limitations have been overcome in our study, where patient-derived and functional in cellulo data converged to define the transcriptional program of the TAL cell of origin and to decipher the mechanistic details of a reversible transcriptional switch driving their transformation into epithelial- and mesenchymal-type RMC states. We thus highlight the fundamental difference between RMC arising from mesoderm-derived differentiated epithelial TAL cells and RT derived from differentiating neural crest cells.

### A link between RMC ferroptosis and sickle cell trait

A key finding of our study is activation of a ferroptosis resistance pathway in RMC cells. Analyses of gene expression signatures in scRNA-seq, patient cohort RNA-seq and the RMC cell lines defined how the ferroptosis sensitivity signature in TAL cells is replaced by a ferroptosis resistance signature in RMC cells. This process is reversed in RMC cells upon SMARCB1 re-expression leading to their ferroptotic cell death unlike other RT cells where SMARCB1 re-expression leads to cell cycle arrest or apoptosis^49,50,53^. Indeed, RMC cells are more sensitive to GPX4 inhibition than RT lines. Ferroptosis is therefore a specific vulnerability of RMC tumours.

The above observations link the RMC oncogenic process with sickle cell trait. The kidney medulla is amongst the most hypoxic micro-environments in the organism^54^. Due to its central role in urine concentration, the loop of Henle is characterized by increasing osmolarity and hypoxia that are highest in the TAL region. Msaouel et al. proposed a model where the high interstitial NaCl concentration induces DNA double strand breaks (DSB), whereas microcirculatory ischaemia induced by red blood cell (RBC) sickling reduces this osmolarity reactivating DSB repair in a chronic hypoxic environment by NEHJ favoring translocations and deletions, particularly in fragile regions such as chromosome 22q where the *SMARCB1* locus is located^55^.

Our observations enrich this model with iron release by RBC sickling favouring ferroptosis of TAL cells and their renewal to maintain the homoeostasis of the epithelium^56,57^. Early initiation of ferroptosis resistance observed in the pre-tumoural TAL cells would thus promote their survival under the high NaCl and hypoxic conditions driving error-prone DSB repair. The increased extracellular iron concentration due to the fragility of the sickled RBCs acts as a selective pressure for survival of ferroptosis resistant TAL cells in an environment propitious to the mutagenic events associated with RMC development. This unique set of circumstances may explain why RMC is the only SMARCB1-deficient tumour arising from epithelial cells, compared to RTs arising from a developmental block of neural crest differentiation.

## Methods

### Ethical approval

Tumour sample collection for further research analysis was approved by ethical Committees of Strasbourg University Hospital and Curie Institute and all patients provided an informed written consent for the use of material for further research. Animal care and use for this study were performed in accordance with the recommendations of the European Community (2010/63/UE) for the care and use of laboratory animals and carried out in accordance with the principles of the Declaration of Helsinki and with GDPR regulations. The experiments were approved by the Curie Institute animal ethical committee CEEA-IC #118 (Authorization APAFIS#11206-2017090816044613-v2 given by National Authority) and performed in accordance with the internal, national and European guidelines of Animal Care and Use. The establishment of PDX received approval by the Institut Curie institutional review board OBS170323 CPP ref 3272; n de dossier (2015- A00464-45). Written institutional informed consent was obtained from the patient.

### Tumour samples

The two RMC samples subjected to scRNAseq were collected from Strasbourg University Hospital and Curie Institute, according to institutional guidelines. One tumour came from a 21-year-old female patient on a post-treatment primary nephrectomy from an RMC patient with lung metastases at diagnosis, whereas the second came from a 16-year-old male patient with regional lymph node and adrenal gland metastases (pT4N1M1). Bulk RNAseq came from 11 patients recently reported^6^ and we generated an additional dataset of multi-region RNAseq of a cohort of 4 of the RMC patients, including multiple sections and lymph nodes metastasis (Supplementary Data 2).

### Human single-cell sample preparation and RNA-seq

Following the treated tumour resection, samples from the tumour and adjacent non-malignant normal adjacent tissue were each conserved at 4 °C in 1 mL of MACS Tissue Storage Solution (Miltenyi Biotech). Single cell suspensions were prepared using gentleMACS^TM^ dissociator and human tumour dissociation kit (Miltenyi Biotech) following manufacturer’s instructions. Samples were applied to a MACS SmartStrainer 70 µm (Miltenyi Biotech) placed on a 15 mL Falcon tube and 10 mL DMEM were used to wash C tube and SmartStrainer. Following centrifugation at 300 g and 4 °C for 10 min, cells were sorted using CD45 (TIL) Microbeads (Miltenyi Biotech). CD45+ and CD45- fractions were centrifuged (300 g, 10 min, 4 °C) and dead cells were removed using Dead cell removal kit (Miltenyi Biotech). CD45- and CD45+ were mixed in 1 to 4 ratios. Cell viability and concentration were assessed before 3′-mRNA single-cell libraries were prepared using the Chromium (10x Genomics) following the manufacturer’s instructions. Libraries were sequenced 2x100bp on HiSeq4000 sequencer.

Folowing resection of the naive tumour, the sample was cut in small pieces then dissociated 30 min at 37 ^°^C in CO2-independent medium (Gibco) + 0,4 g/l of human albumin (Vialebex) with Liberase TL (Roche) 150 ug/ml and DNase 1 (Sigma) 150 μg/ml. Dissociated cells were then filtered with a 40 mm cell strainer, then washed and resuspended with CO2-independent medium + 0,4 g/l of human albumin. A fraction of the cell suspension was used to enrich tumour cells using Tumour isolation kit (Miltenyi Biotech, cat#130-108-339). Cells were then resuspended at 800 cells/ul in PBS + BSA 0,04%. Tissues were processed within 1 h after tumour resection, and sorted cells were loaded in a 10x Chromium instrument within 6 h.

### Patient-derived xenograft sample preparation

Renal medullary carcinoma (RMC) patient derived xenograft (IC-pPDX-132) was established from a resected RMC tumour treated with 6 cycles of cisplatin, gemcitabine and bevacizumab. The undissociated tumour was engrafted in the subscapular fat pad of NSG (NOD.Cg-Prkdc^scid^ IL2^rgtm1Wjl^/SzJ) mice. A PDX tumour fragment was then serially transplanted using the same procedure into Swiss Nude (Crl:NU(Ico)-Foxn1^nu^) mice until passage 4 which was used for the single cell RNA-seq experiments. No PDX tumour was allowed to grow beyond the 1000 mm3 size limitation. Mice were maintained in IVC cages in a semi pathogen-free facility under standard housing conditions with continuous access to food and water. Curie Institute animal facilities comply with all appropriate standards (cages, space per animal, temperature (22 °C), light, 12-hour light/dark cycle, 50% humidity, continuous access to food and water), and all cages are enriched with nesting materials.

### scRNA-seq analysis of human primary RMC tumours

After sequencing, raw reads were processed using CellRanger (v 3.1) to align on the hg19 human genome, remove unexpressed genes and quantify barcodes and UMIs. Data were then analysed in R (v4.0.2). For the treated tumour, tumour and NAT samples were aggregated with the cellranger ‘aggr’ command. The resulting aggregation was analysed with Seurat v3.2.0 following the recommended workflow. Cells were filtered for feature count ranging from 120 to 2000 and percentage of mitochondrial reads <15%. Counts were normalized with the “LogNormalize” method and data scaled to remove unwanted sources of variation (UMI count and mitochondrial reads). The number of principal components was determined from the Jackstraw plots. Clustering was performed on variable features using the 25 most significant principal components and a resolution of 1.15. For the naive tumour, the same Seurat pipeline was performed using feature counts from 200 to 6000, mitochondrial read fraction <20% and a resolution of 1.0 using the 20 most significant principal component for the clustering. Aggregate analyses of tumours 1 and 2 was performed by merging the two R objects and using the Seurat sctransform with batch correction function to normalize and scale data reducing the impact of technical factors.

### scRNA-seq analysis of patient-derived RMC xenograft

For the IC-pPDX-132 sample raw reads were aligned on an hg19-mm10 hybrid genome. Cells were filtered based on feature counts ranging from 200 to 7000 and global clustering performed with a resolution of 0.3 using the 20 most significant components. Human and Mouse cells were re-clustered separately by first filtering cells with mitochondrial read fraction >20% and then using a resolution of 0.4 with 25 principal components.

### Functional analysis using scRNA-seq data

Regulome analyses of active transcription factors were performed using the SCENIC v1.1.2.2 package^17^. Transcription factor activities were visualized on the UMAP using AUCell v1.8.0 or as heatmaps using the R-package ‘pheatmap’. RMC correlations with the different renal tubule clusters were computed by Clustifyr v1.0.0^58^ using cluster marker signatures for RMC (*TIMP1, FN1, CTHRC1, DCBLD2, COL1A2, COL1A1, ARL4C, COL6A2, LGALS1, CD44, VIM, CLU, MMP7, SERPINA1, WFDC2, SFRP2, MUC1, KRT18, KRT7, EPCAM, CDH1, CLDN4, CLDN10, DEFB1*), RMC1 (*WFDC2, FXYD2, SLPI, CLDN4, KRT7, KLF6, GSTP1, EEF1A1, CLDN3, TM4SF1*) and RMC2 (*FN1, COL1A2, COL1A1, TIMP1, CD44, CTHRC1, RARRES3, BGN, TFPI2, COL6A2*). Trajectory analyses were plotted and visualized using Similarity Weighted Nonnegative Embedding (SWNE)^59^. Gene set variation analysis were performed using the r-package GSVA^60^.

For the “bulk RMC signature”, the upregulated genes from the differential analysis of the MDACC RMC cohort (11 tumours versus 6 NAT) were selected using log2FC > 2 and FDR < 0.01^6^. For all signatures, gene sets were retrieved from either Hallmarks MSigDB or KEGG pathways. Gene signatures were computed and visualized on UMAPs using the R package VISION v2.1.0 (https://github.com/YosefLab/VISION).

### Anti-4 Hydroxynonenal staining of RMC tumours

Sections from 2 independent RMC tumours and as control a colorectal cancer were fixed in 10% neutral-buffered formalin, paraffin embedded, sectioned, and stained with hematoxylin and eosin. 4-μm tissue sections were processed on VENTANA-Benchmark-XT, with incubation at room temperature in an antigen retrieval process (EDTA citrate buffer, pH 8,3, CC1 buffer, 8 mn), then incubated with 4HNJ-2 (Anti-4 Hydroxynonenal antibody, mouse monoclonal, clone HNEJ-2, Abcam; dilution: 1/5000 during 32 mn), revealed with ‘Ultra View’ Universal DAB Detection kit and counterstained with Hematoxylin solution (Ventana Roche Systems).

### Cell culture, establishment of RMC lines stably expressing SMARCB1

RMC219 cells were grown in HAM-F12/D-MEM (1:1) medium supplemented with 10% foetal calf serum (FCS), Glutamine 2 mM, AANE and PS. UOK360 and UOK353 cells were grown in D-MEM medium supplemented with 10% foetal calf serum (FCS) and Glutamine 2 mM. RMC2C cells were grown in MEM medium with 10% FCS, AANE, 50 ng/mL EGF and PS. Cell lines were provided by colleagues and are not commercially available. Authentication performed by immunoblot showing absence of SMARCB1 expression and by RNA-seq. HEK 293 T cells were obtained from ATCC. All cell lines were regularly tested as negative for Mycoplasma infection using the Venor™ GeM Mycoplasma Detection Kit, and used at less than 10 passages. RMC cells infected with lentiviral constructs were grown in respective media replacing normal FCS with tetracyclin-free FCS (Dutscher) and supplemented with G418 (300ug/mL). SMARCB1 expression was induced by treatment with either DMSO or 2 µM of doxycycline.

Lentiviral pInducer20 vector was obtained from Addgene and the cDNA of either SMARCB1 or mCherry was cloned into the vector by Gateway. We then used pInducer20-mCherry or -SMARCB1 containing lentiviruses to infect 1×10^6^ RMC2C or RMC219 cells. Cells were selected using 500ug/mL G418 for a week and then maintained under these conditions.

### In vitro treatments

For ferroptosis, cells were either treated with DMSO or 2uM doxycycline alone or co-treated with 2uM doxycycline and 1uM ferrostatin-1 (SelleckChem, #S7243), zVAD-fmk (MedChemExpress, #HY-16658B) or necrostatin-1 (MedChemExpress, #HY-15760) for the indicated times. For the Caspase-3 assays, cells were either treated with 5uM camptothecin (SelleckChem, #S1288) for 4 h, DMSO or 2uM doxycycline for the indicated times. For the IFNg experiments, cells were either treated with DMSO or 10 ng/mL of IFNg (Peprotech, 300-02).

### Cell death, caspase-3 and lipid peroxidation analyses by flow cytometry

Cells were harvested at the indicated times and co-stained with Annexin-V-FITC and propidium iodide following manufacturer instructions (BioLegend, #640914). To assess active Caspase-3, cells were fixed and permeabilized before incubation with the FITC-conjugated caspase-3 antibody (dilution 1/6) following manufacturer’s instructions (BD Biosciences # 550914) for subsequent flow cytometry analysis. To assess membrane lipid perodixation, cells were stained using 10uM of Bodipy 581/591 C11 (ThermoFisher, #D3861) following manufacturer’s instructions. To assess senescence, cells were treated with 100 nM bafilomycin A1 (Sigma, #19-148) for 1 h followed by 2 mM C12FDG (Invitrogen, #D2893) for 2 h before being washed and harvested for flow cytometry analyses. All assays were analysed on a LSRII Fortessa (BD Biosciences) and data were analysed using Flowjo v6.8.

### Immunofluorescence

Cells grown on glass slides in 24-well plates, were fixed with 4% paraformaldheyde for 15 min. After two washes with PBS buffer, they were permeabilized in PBS+triton X-100 0,1% for 5 min and blocked with PBS + 10% FCS inactivated for 20 min. Primary antibodies were incubated overnight at 4 °C and after three washes with PBS+Triton 0,1%, cells were stained for 1 h at room temperature with AlexaFluor-488 conjugated secondary antibodies (Invitrogen goat anti mouse **#** A11001 and goat anti-rabbit **#** A32731) diluted 1/500 in PBS + 10% FCS. After three washes with PBS+Triton 0,1%, cells were stained with DAPI (final concentration 1 ug/ml) and mounted on microscopy slides. Images were captured with a confocal (Leica DMI6000) microscope.

### Cell viability assay by fluorescence screening

In total, 5 × 10^3^ of indicated cell types were seeded on 96-well plates in four technical replicates on day 1. The next day, cells were treated either with DMSO control or with an increasing concentration of RSL3 (SelleckChem, 8155) ranging from 0 to 10 µM. At day 3, cells were washed with PBS and stained using PrestoBlue (Invitrogen, A13261) according to manufacturer instructions before fluorescence was quantified on a multi-modal spectrometer (Berthold Mithras, LB940). IC50 values were calculated using the fraction of DMSO control.

### Immunostaining quantification by flow cytometry

Wildtype RMC219 and RMC2C cells were harvested and 1 × 10^6^ cells were resuspended in buffer A (PBS 1X, EDTA 2 mM, inactivated FCS 1%) and 5uL of Human TruStain FcX (Biolegend, 422301) was added for 10 min at room temperature. Following blocking, cells were stained for 1 h with 5 µL of conjugated EPCAM-FITC (Biolegend, 324203) and conjugated CD44-PE (Biolegend, 103023). Following two PBS washes, cells were resuspended in buffer A before flow cytometry on a LSRII Fortessa (BD Biosciences) and analysis using Flowjo v6.8.

### Boyden Chamber Invasion assays

Before seeding, 100ul of diluted Matrigel (1:20, 356234, Corning) was added in each insert (24-well 8um inserts, Corning) and left to dry for 2 h at 37 °C before being washed twice with PBS. Subsequently, RMC cells were harvested and 2 × 10^5^ cells and seeded in the Boyden chambers in corresponding media without serum. 24 h later, migrated cells were fixed using PFA 4% for 10 min before being stained using Crystal violet for 10 min. Excess stain was washed 3 times in PBS before images were captured on phase contrast microscope. Quantification of migrated cells was done by resuspension of staining using 100 mM acetic acid for 15 min before luminescence was measured on a BioTek Luminescence microplate reader (using Gen5 software).

### RNA preparation and quantitative PCR

RNA isolation was performed according to standard procedures (Macherey Nagel RNA Plus kit). RT-qPCR was carried out with SYBR Green I (Roche) and SuperScript IV Reverse Transcriptase (Invitrogen) and monitored using a LightCycler 480 (Roche). The mean of ACTB, TBP, RPL13A and GAPDH gene expressions was used to normalize the results. Primer sequences for each cDNA were designed using Primer3 Software and are available in Supplementary Table 1.

### Public data correlation analysis using TGCA and CCLE database

Spearman correlation for all selected genes were retrieved from co-expression studies using the Cancer Cell Line Encyclopaedia (Broad, 2019) and the TCGA chromophobe renal cell carcinoma (KICH) databases. All transcription factors were extracted using the “Full Human TFs” list from^61^. Scatter plots were made using Prism5. For the correlation with TFCP2L1, the epithelial and mesenchymal genes were retrieved from Watanabe et al.^23^.

### Bulk RNA sequencing

RMC cell lines were analysed by RNA-seq under the different indicated conditions. After sequencing raw reads were pre-processed in order to remove adapter and low-quality sequences (Phred quality score below 20) using cutadapt version 1.10. and reads shorter than 40 bases were discarded. Reads were maping to rRNA sequences using bowtie version 2.2.8, were also removed. Reads were mapped onto the hg19 assembly of Homo sapiens genome using STAR version 2.5.3a. Gene expression quantification was performed from uniquely aligned reads using htseq-count version 0.6.1p1, with annotations from Ensembl version 75 and “union” mode. Only non-ambiguously assigned reads were retained for further analyses. Read counts were normalized across samples with the median-of-ratios method. Comparisons of interest were performed using the Wald test for differential expression and implemented in the Bioconductor package DESeq2 version 1.16.1. Genes with high Cook’s distance were filtered out and independent filtering based on the mean of normalized counts was performed. P-values were adjusted for multiple testing using the Benjamini and Hochberg method. Deregulated genes were defined as genes with log2(foldchange) >1 or <−1 and adjusted *p* value < 0.05.

### Analysis of bulk RNA-seq of patient samples

For RMC cohorts, raw counts were retrieved in excel format and normalized first by sequencing depth using DESeq2 sizefactors and then divided by median of gene length. Samples were clustered using the hclust function with “ward.D2” linkage function and visualized as heatmaps using pheatmap package v1.0.12. The deconvolution of immune and stromal cells was done using MCP-counter v1.2.0^62^. Sample compositions were also estimated by deconvolution from our single-cell data using the CIBERSORTx algorithm^63^. Volcano plots were generated with ggplot2 v3.3.2. Gene set enrichment analyses were done with the GSEA software v3.0 using the hallmark gene sets of Molecular Signature Database v6.2. Gene Ontology analysis was done using DAVID (http://david.abcc.ncifcrf.gov/). Gene list intersections and Venn diagrams were performed by Venny.

### Protein extraction and Western blotting

Whole cell extracts were prepared by the standard freeze-thaw technique using LSDB 500 buffer (500 mM KCl, 25 mM Tris at pH 7.9, 10% glycerol (v/v), 0.05% NP-40 (v/v), 16 mM DTT, and protease inhibitor cocktail). Cell lysates were subjected to SDS–polyacrylamide gel electrophoresis (SDS-PAGE) and proteins were transferred onto a nitrocellulose membrane. Membranes were incubated with primary antibodies in 5% dry fat milk and 0.01% Tween-20 overnight at 4 °C. The membrane was then incubated with HRP-conjugated secondary antibody (Jackson ImmunoResearch; Goat against Mouse: 115-036-71; Goat against Rabbit: 111-035-144 dilution 1.2000) for 1 h at room temperature, and visualized using the ECL detection system (GE Healthcare). The references of all antibodies are available in Supplementary Table 2.

### Chromatin immunoprecipitation and sequencing (ChIP-seq)

BRG1 ChIP experiments were performed on native MNase-digested chromatin. Between 10 to 20 × 10^8^ freshly harvested RMC2C cells bearing either SMARCB1 or mCHERRY and treated 2uM doxycycline for 48 h were resuspended in 1.5 ml ice-cold hypotonic buffer (0.3 M Sucrose, 60 mM KCl, 15 mM NaCl, 5 mM MgCl2, 0.1 mM EDTA, 15 mM Tris–HCl pH 7.5, 0.5 mM DTT, 0.1 mM PMSF, PIC) and cytoplasmic fraction was released by incubation with 1.5 ml of lysis-buffer (0.3 M sucrose, 60 mM KCl, 15 mM NaCl, 5 mM MgCl2, 0.1 mM EDTA, 15 mM Tris–HCl pH 7.5, 0.5 mM DTT, 0.1 mM PMSF, PIC, 0.5% (vol/vol) IGEPAL CA-630) for 10 min on ice. The suspension was layered onto a sucrose cushion (1.2 M sucrose, 60 mM KCl, 15 mM NaCl, 5 mM MgCl2, 0.1 mM EDTA, 15 mM Tris–HCl [pH 7.5], 0.5 mM DTT, 0.1 mM PMSF, PIC) and centrifuged for 30 min 4 °C at 5000 g in a swing rotor. The nuclear pellet was resuspended in digestion buffer (0.32Msucrose, 50 mM Tris–HCl [pH 7.5], 4 mM MgCl2, 1 mM CaCl2, 0.1 mM PMSF) and incubated with 10ul of Micrococcal Nuclease (NEB) for 7 min at 37˚C. The reaction was stopped by addition of 20ul of EDTA 0,5 M and suspension chilled on ice for 10 min. The suspension was cleared by centrifugation at 8000 g (4˚C) for 10 min and supernatant (chromatin) was used for further purposes. Chromatin was digested to around 80% of mono-nucleosomes as judged by extraction of the DNA and agarose gel electrophoresis. H3K27ac and MYC ChIP experiments were performed on 0.4% PFA-fixed chromatin isolated from RMC2C cells bearing either SMARCB1 or mCHERRY and treated 2uM doxycycline for 48 h according to standard protocols^64^. ChIP-seq libraries were prepared using MicroPlex Library Preparation kit v2 and sequenced on the Illumina Hi-seq 4000 as single-end 50-base reads^65^. Sequenced reads were mapped to the Homo sapiens genome assembly hg19 using Bowtie with the following arguments: -m 1 --strata --best -y -S -l 40 -p 2. Cut&Tag was performed using the Active Motif CUT&Tag-IT kit following the manufacturer’s instructions.

### ChIP-seq analysis

After sequencing, peak detection was performed using the MACS software (Zhang et al., 2008). Peaks were annotated with Homer using the GTF from ENSEMBL v75. Global clustering analysis and quantitative comparisons were performed using seqMINER^66^. Super-enhancers were called with the python package Ranking Of Super Enhancers (ROSE) https://github.com/stjude/ROSE.

De novo motif discovery on FASTA sequences corresponding to windowed peaks was performed using MEME suite (meme-suite.org). Motif correlation matrix was calculated with in-house algorithms using JASPAR database as described in^67^. Motif discovery from ChIP-seq peaks was performed using the RSAT peak-motifs algorithm (http://rsat.sb-roscoff.fr/peak-motifs_form.cgi).

Motif analysis Searching of known TF motifs from the Jaspar 2014 motif database at BRG1-bound sites was made using FIMO^68^ within regions of 200 bp around peak summits, FIMO results were then processed by a custom Perl script which computed the frequency of occurrence of each motif. To assess the enrichment of motifs within the regions of interest, the same analysis was done 100 times on randomly selected regions of the same length as the BRG1 bound regions and the results used to compute an expected distribution of motif occurrence. The significance of the motif occurrence at the BRG1-occupied regions was estimated through the computation of a Z-score (z) with z = (x − μ)/σ, where: − x is the observed value (number of motif occurrence), − μ is the mean of the number of occurrences (computed on randomly selected data), − σ is the standard deviation of the number of occurrences of motifs (computed on randomly selected data). The source code is accessible at https://github.com/slegras/motif-search-significance.git.

### Statistics

All experiments were performed in biological triplicates, unless stated otherwise in the figure legends. All tests used for statistical significance were calculated using Prism5 and indicated in the figure legends along with *p* values (****p < 0.0001, ***p < 0.001, **p < 0.01, *p < 0.05, ns: *p* > 0.05).

### Reporting summary

Further information on research design is available in the Nature Portfolio Reporting Summary linked to this article.

## Supplementary information

Supplementary Information

Description of Additional Supplementary Files

Supplementary Data 1

Supplementary Data 2

Supplementary Data 3

Reporting Summary

## Data Availability

All data used in this study are available in the main article or as supplementary information to the manuscript. Source data are provided as a Source Data file. The sequencing data used in this study are publicly available in the GEO database under accession number GSE181001. Source data are provided with this paper.

## Source data

Source Data
