# Supplementary material for: SMARCB1 regulates a TFCP2L1-MYC transcriptional switch promoting renal medullary carcinoma transformation and ferroptosis resistance
Source: Nat Commun. 2023 May 26;14:3034. doi: 10.1038/s41467-023-38472-y (PMC10220073; doi:10.1038/s41467-023-38472-y)
Supplement: Supplementary file 1 — Supplementary Information [file 41467_2023_38472_MOESM1_ESM.pdf]

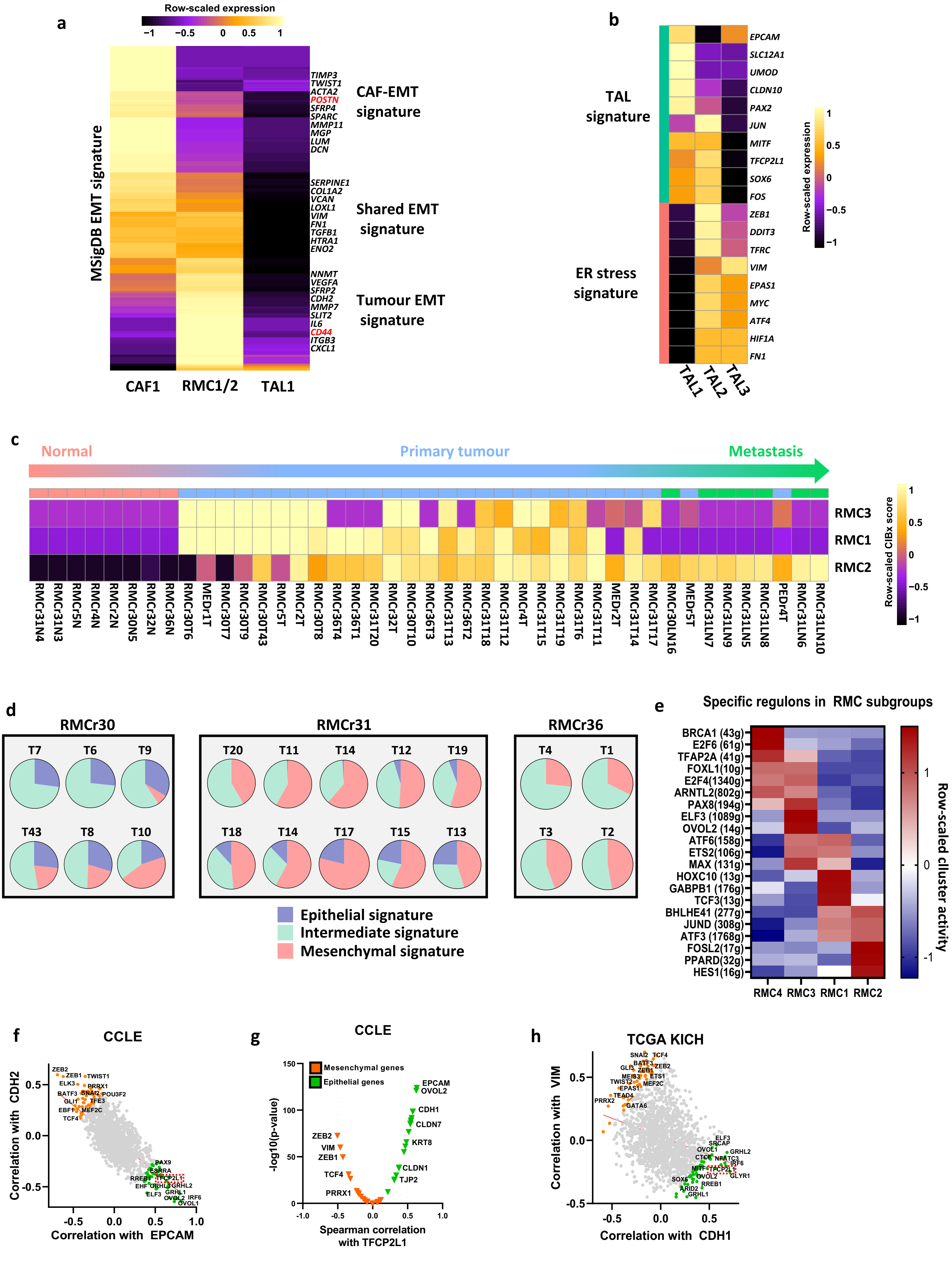

Vokshi et al., Suppl. Fig. 1

Supplementary Figure 1. a. Pseudo-bulk heatmap showing expression of the MSigDB Hallmark EMT signature (183 genes) in RMC1/2 and CAF1 cells from the treated tumour. **b.** Pseudo-bulk heatmap showing heterogeneous expression of selected TAL identity markers, mesenchymal and ER stress genes in all TAL clusters. **c.** Deconvolution of RMC specific signatures as calculated by CIBERSORTx on bulk RNA-seq from sections of RMC primary tumours, lymph node metastasis and normal adjacent tissues. **d.** Pie charts representing intratumoural heterogeneity of RMC signatures using multi-region RNA sequencing of primary RMC tumours (n=3). Note that relative proportions (in %) were inferred by CIBERSORTx using our scRNA-seq normalized merge**. e.** SCENIC analysis of normalized merge of treated and naive RMC samples revealing specific regulons of all RMC clusters. **f.** Pearson correlation analysis of 1683 human transcription factors with selected genes in CCLE database. **g.** Pearson correlation analysis of TFCP2L1 expression with a set of epithelial and mesenchymal genes in CCLE database. P-values were calculated using a two-sided t-test corrected with Benjamin-Hochberg FDR adjustment. **h.** Pearson correlation analysis of 1683 human transcription factors with selected genes in TGCA KICH RCC samples. Note the positive correlation of TFCP2L1 expression level with epithelial markers along with other Grainyhead family members, OVOL1/2 and MITF.

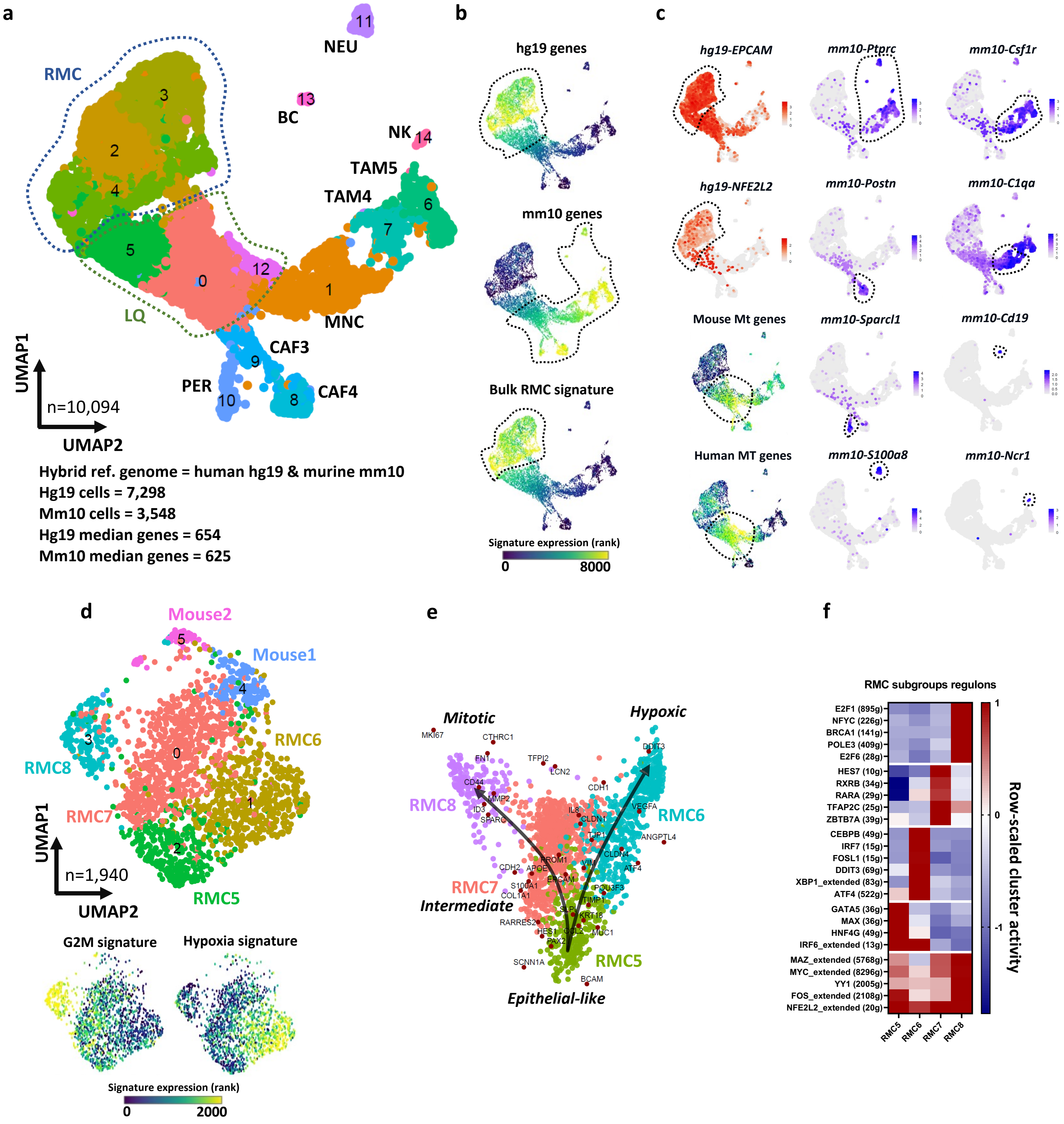

Vokshi et al., Suppl. Fig. 2

Supplementary Figure 2. a. UMAP plot of scRNA-seq from the RMC PDX (IC-pPDX-132) representing cell clusters as calculated by Seurat at a resolution of 0.3. Clusters were identified using hallmark genes shown in Fig. 3b-c. *Abbreviations:* RMC: Renal medullary carcinoma cells; LQ: low quality cells; PER: pericytes; CAF3/4: cancer-associated fibroblasts; MNC: monocytes; TAM4/5: tumour-associated macrophages; NK: natural killers; BC: B-cells; NEU: neutrophils. **b.** UMAP projection of selected gene signatures. Human and murine signatures were established using differential gene nomenclature. **c.** UMAP projection of marker genes and mitochondrial gene signatures. Human and murine mitochondrial gene signatures were established using differential gene nomenclature. **d**. UMAP representing PDX RMC subclusters as identified by Seurat using a resolution of 1 (upper panel), Average expression of selected MSigDB gene signatures (lower panel). **e.** SWNE trajectory analysis of RMC cells using markers of each cluster. **f.** SCENIC analysis of RMC subclusters.

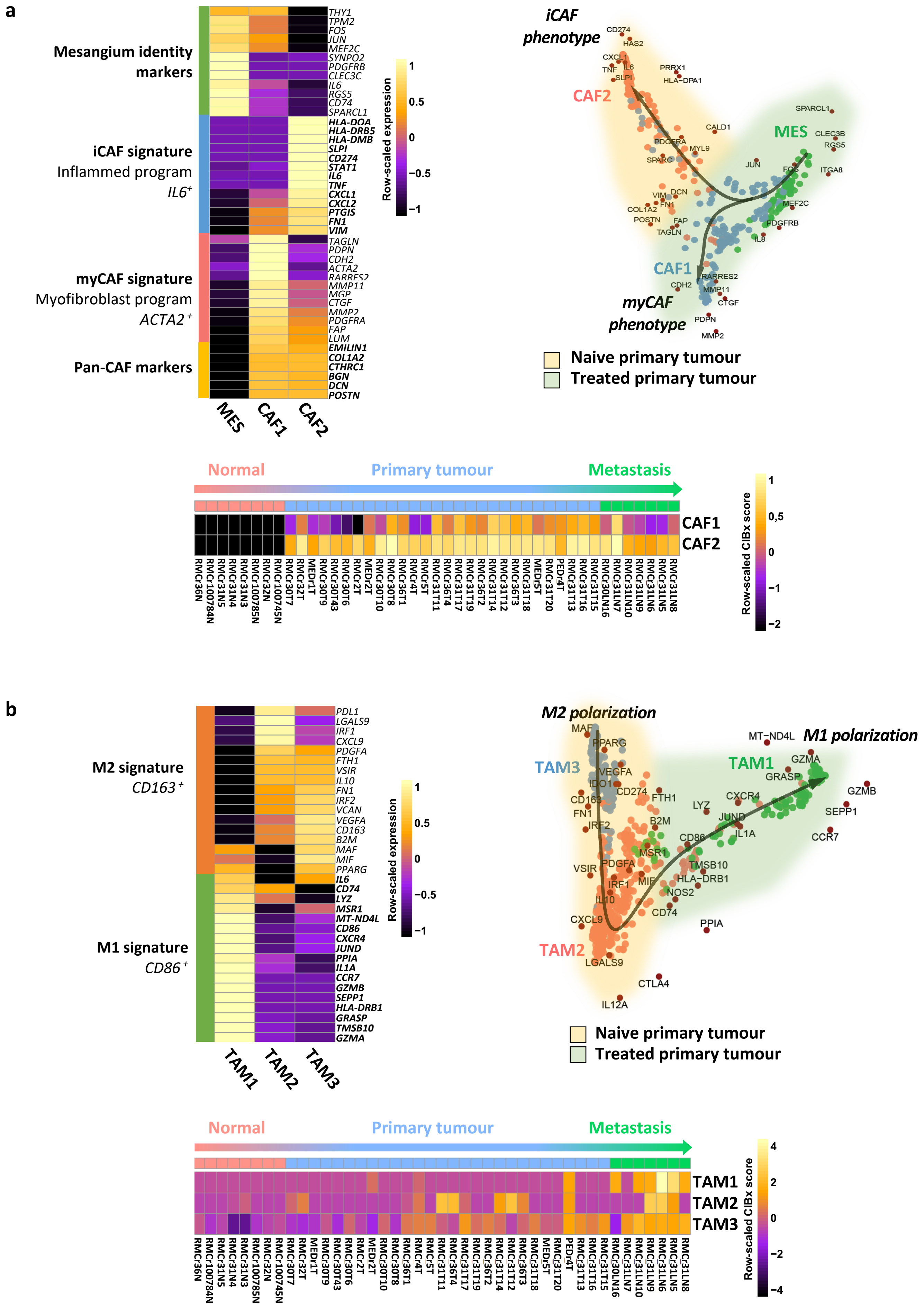

Vokshi et al., Suppl. Fig. 3

Supplementary Figure 3. a. Pseudo-bulk heatmap of iCAF and myCAF signature genes as well as MES and CAF identity markers in CAF clusters and their putative MES cell-of-origin. SWNE trajectory analysis using a set of selected CAF and MES markers revealing distinct CAF phenotypes. Arrow indicates the putative trajectory of CAF activation from MES cells. Deconvolution of CAF specific signatures on bulk RMC RNA-seq. **b.** Pseudo-bulk heatmap of macrophages M1 and M2 gene signatures in TAM clusters from the treated and naive tumours. SWNE trajectory analysis of TAM clusters using a set of selected M1/M2 polarization markers revealing distinct TAM phenotypes. Arrow indicates the putative trajectory in the treated RMC sample. Deconvolution of TAM specific signatures on bulk RNA-seq from sections of RMC.

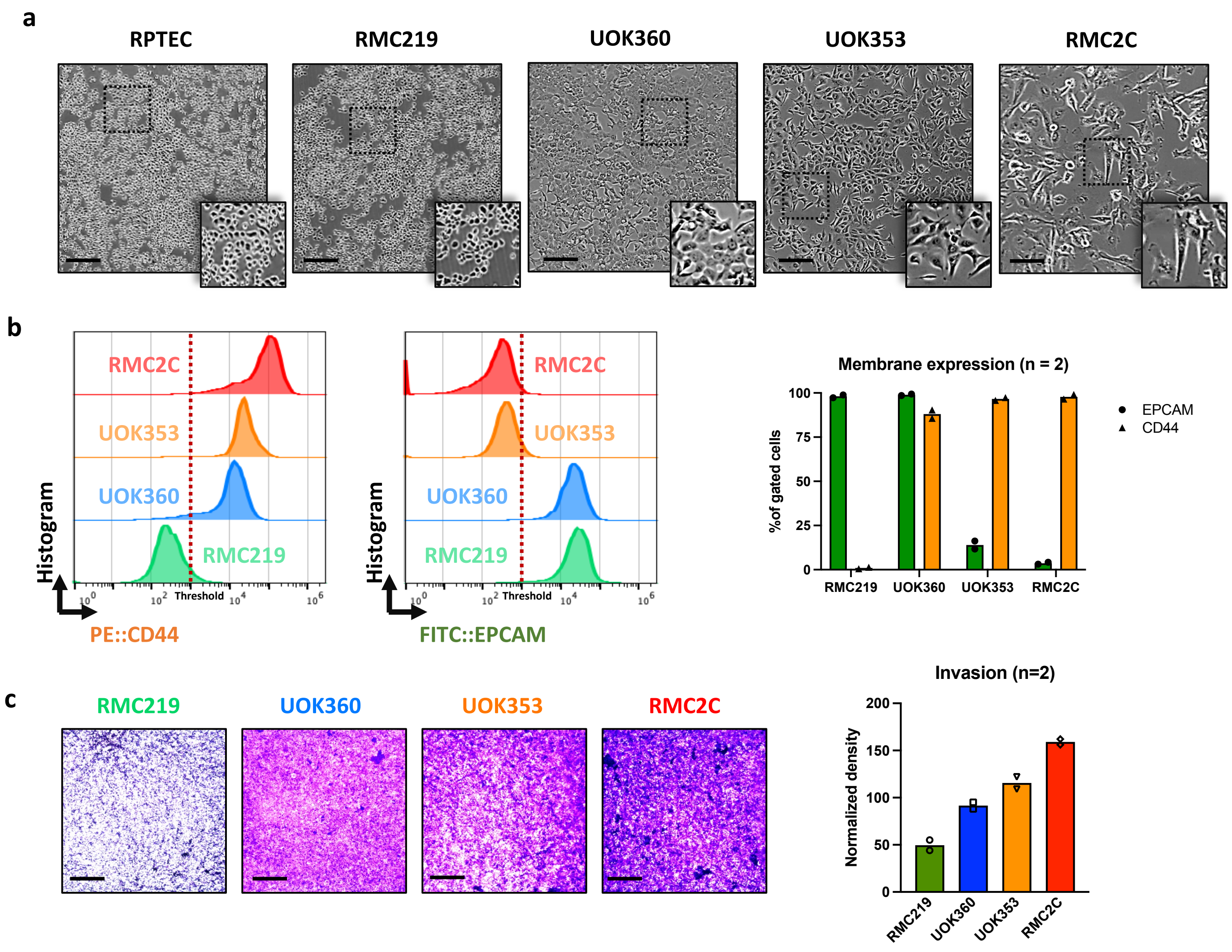

Vokshi et al., Suppl. Fig.4

Supplementary Figure 4. a. Phase-contrast microscopy at 20X magnification of normal kidney (RPTEC) and the indicated tumour cells. Scale bars: 250µm. n=3 independent biological replicates. **b.** Flow cytometry of membrane protein expression of EPCAM and CD44 in RMC lines. n=2 independent biological replicates. **c.** Brightfield microscopy at 4X magnification of Boyden chamber matrigel assays using RMC lines (left) and absolute quantification using absorbance of resuspended crystal violet (right). n=2 independent biological replicates. Scale bars: 1000µm. Source data are provided as a Source Data files 1 and 2.

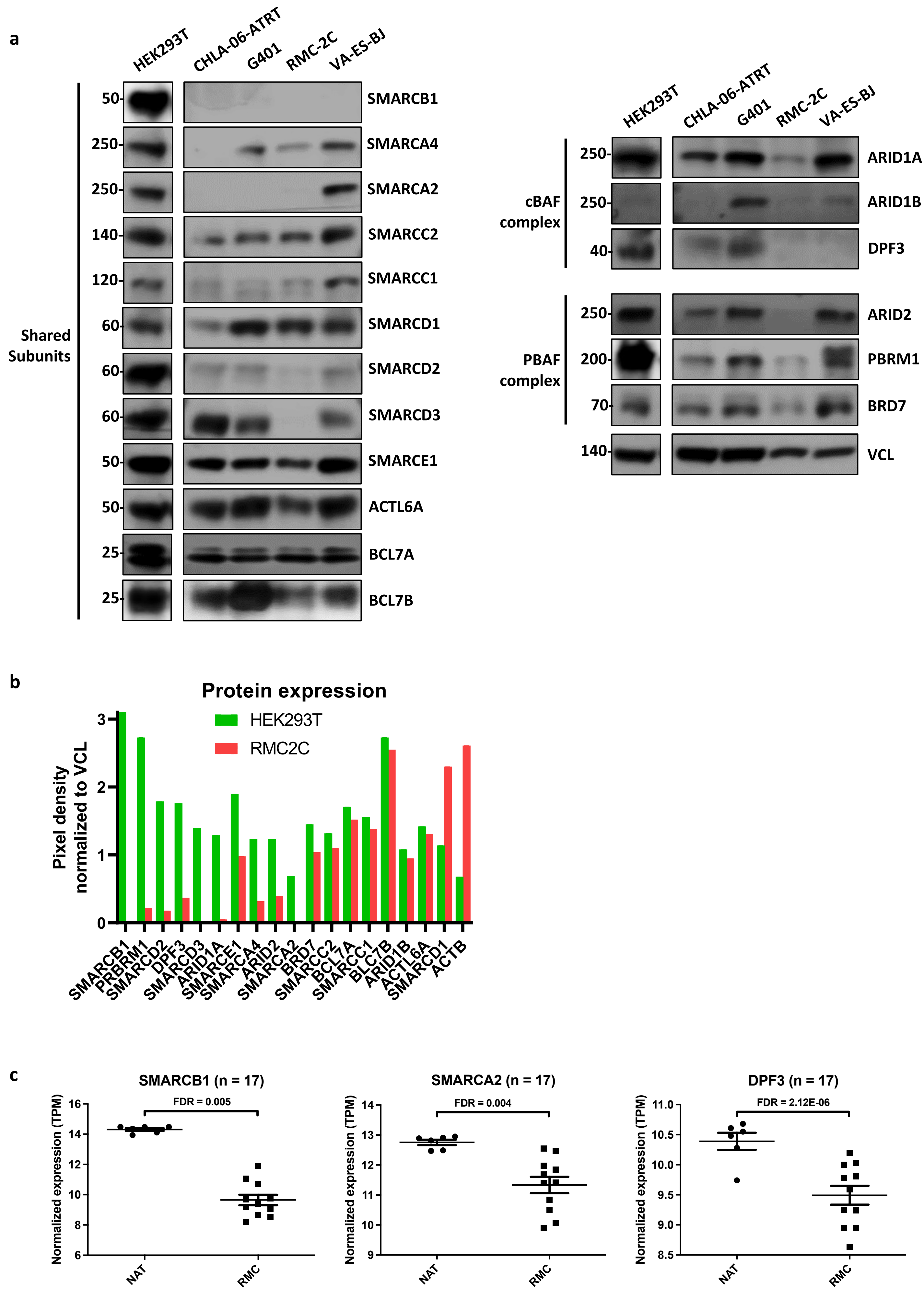

Vokshi et al., Suppl. Fig. 5

Supplementary Figure 5. a. Immunoblots revealing expression of SWI/SNF subunits in RMC2C cells, 3 additional SMARCB1-deficient lines and HEK293T cells. HEK293T: immortalized human embryonic kidney cells; CHLA-06-ATRT: atypical teratoid/rhabdoid tumour cell line; G401: malignant rhabdoid tumour cell line; VA-ES-BJ: epithelioid sarcoma cell line. Loading normalisation: VCL. Molecular mass markers in kDa are indicated. **b**. Quantification of expression of the indicated subunits based on scanning of the immunoblots in panel a and corrected for VCL levels. **c.** Scatter plots showing the expression of a selection of SWI/SNF genes in RMC and normal adjacent tissue (NAT). n=17 biological samples: 11 tumor and 6 normal adjacent tissues. Data are shown as means ± SEM and p-values were derived using the Wald test and adjusted with the Benjamini-Hochberg FDR procedure. Source data are provided as a Source Data file 2.

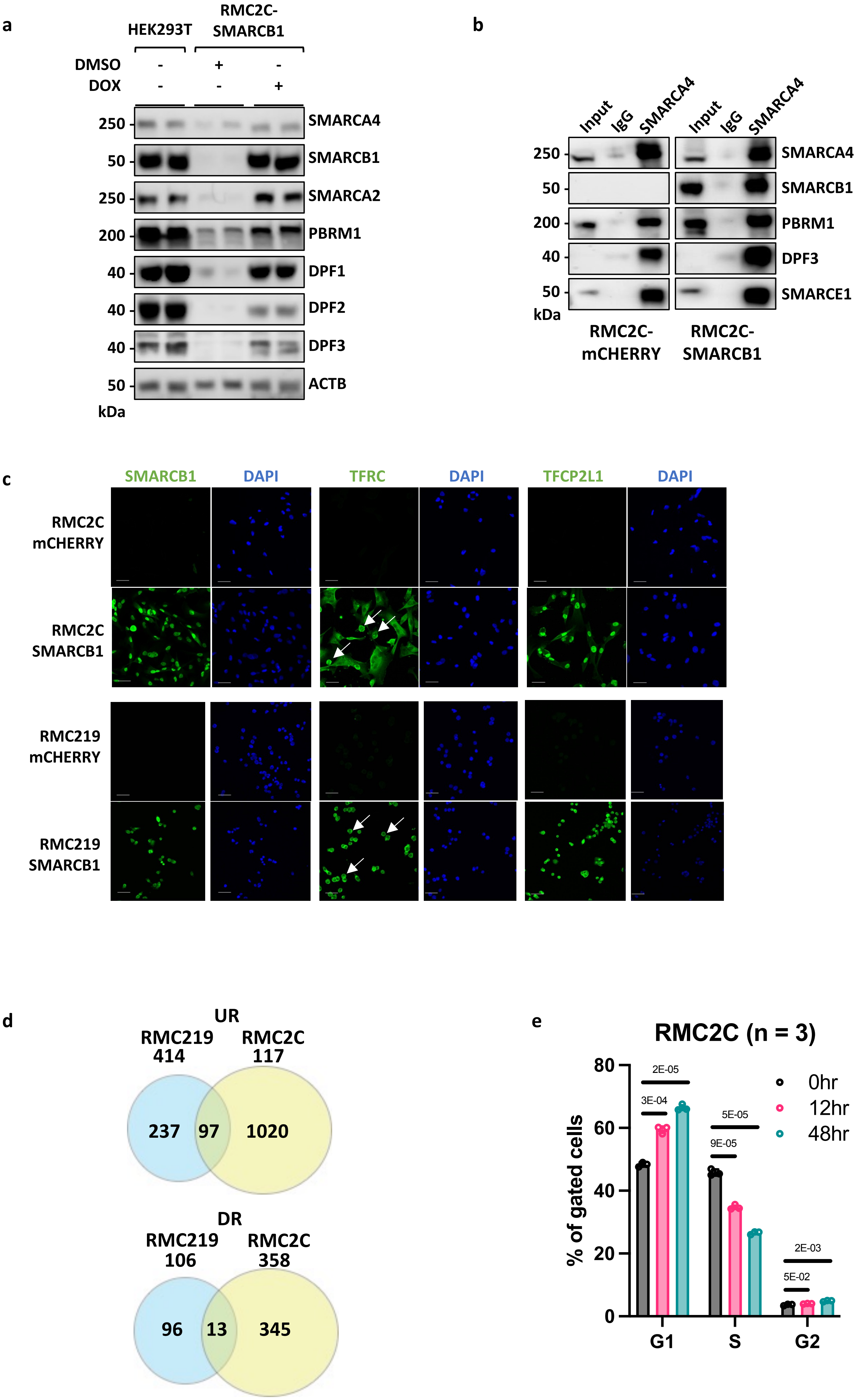

Vokshi et al., Suppl. Fig. 6

Supplementary Figure 6. a. Immunoblots showing re-expression of SMARCB1 and expression of selected SWI/SNF subunits in RMC2C cells with or without Dox compared with HEK293T cells. n=3 independent biological replicates. Molecular mass markers in kDa are indicated. **b**. Co-precipitation of re-expressed SMARCB1 with SWI/SNF subunits in RMC2C cells. n=3 independent biological replicates. Molecular mass markers in kDa are indicated. **c**. Immunostaining of RM2C2 and RMC219 cells 24 hours after Dox treatment with the indicated antibodies. Arrows indicate cells where TFRC is localized at the plasma membrane. Captured on a confocal microscope at 40X magnification, scale bars: 100µm. n=3 independent biological replicates. **d**. Venn diagrams showing overlap between genes up and down regulated in RMC219 and RMC2C cells 48 hours after Dox treatment. **e.** Cell cycle analyses of RMC2C cells at the indicated times after Dox treatment illustrating accumulation of cells at G1/S and G2/M and reduction in S-phase cells. n= 3 independent biological replicates. Data are shown as means ± SEM and p-values as indicated on the graph were calculated with Prism5 using two-sided unpaired Student t-tests. Source data are provided as a Source Data files 1 and 2.

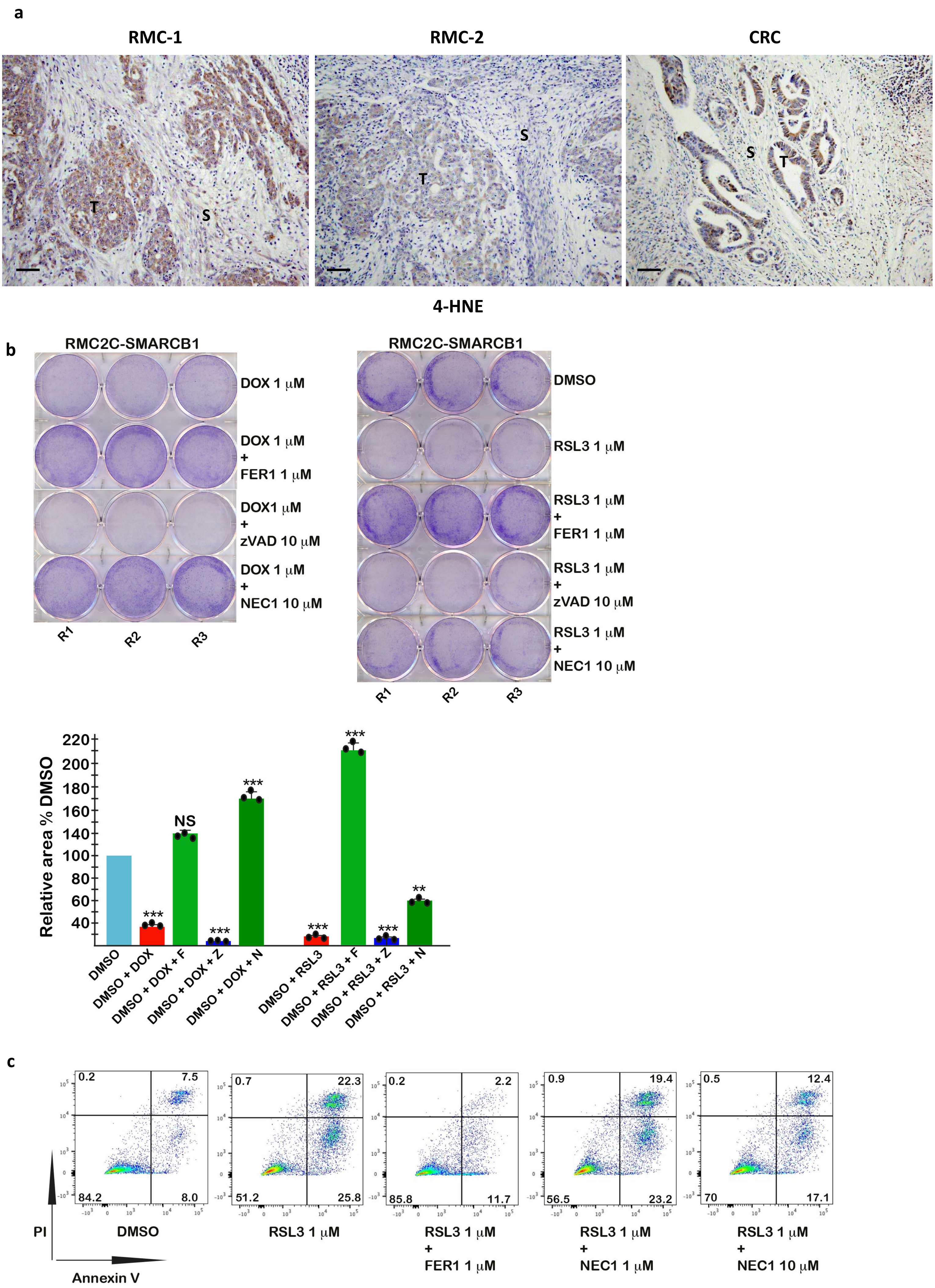

Vokshi et al., Suppl. Fig. 7

Supplementary Figure 7. a. Immunohistochemistry revealing Anti-4 Hydroxynonenal staining of sections from 2 independent RMC tumours and a colorectal tumour as positive control. T = tumour; S = stroma; all images 20X magnification. Scale bars: 100µm. **b.** Cell viability assays in presence of Dox or RSL3 and the indicated inhibitors. 1.5 x10^3 cells were plated and treated as indicated for 72 hours before staining with Crystal Violet and quantification by calculating the area fraction covered by using the FIJI software. The same threshold value was applied to every well and the “relative area” expressed as a fraction of the DMSO control set to 100%. n=3 independent biological replicates. R1-R3 indicate biological replicates, data are shown as means ± SEM and p-values were calculated using a two-way Anova test, ** indicates p<0.01 *** indicates p<0.001, NS non-significant. P values: 0.001, 0.21, 3.74E-13, 7.6E-06, 0.21, 2.2E-05, 8.0E-11, 1.4E-05, 0.004. **c**. Flow cytometry of RSL3-treated cells in presence of the indicated inhibitors. Source data are provided as a Source Data file 1.

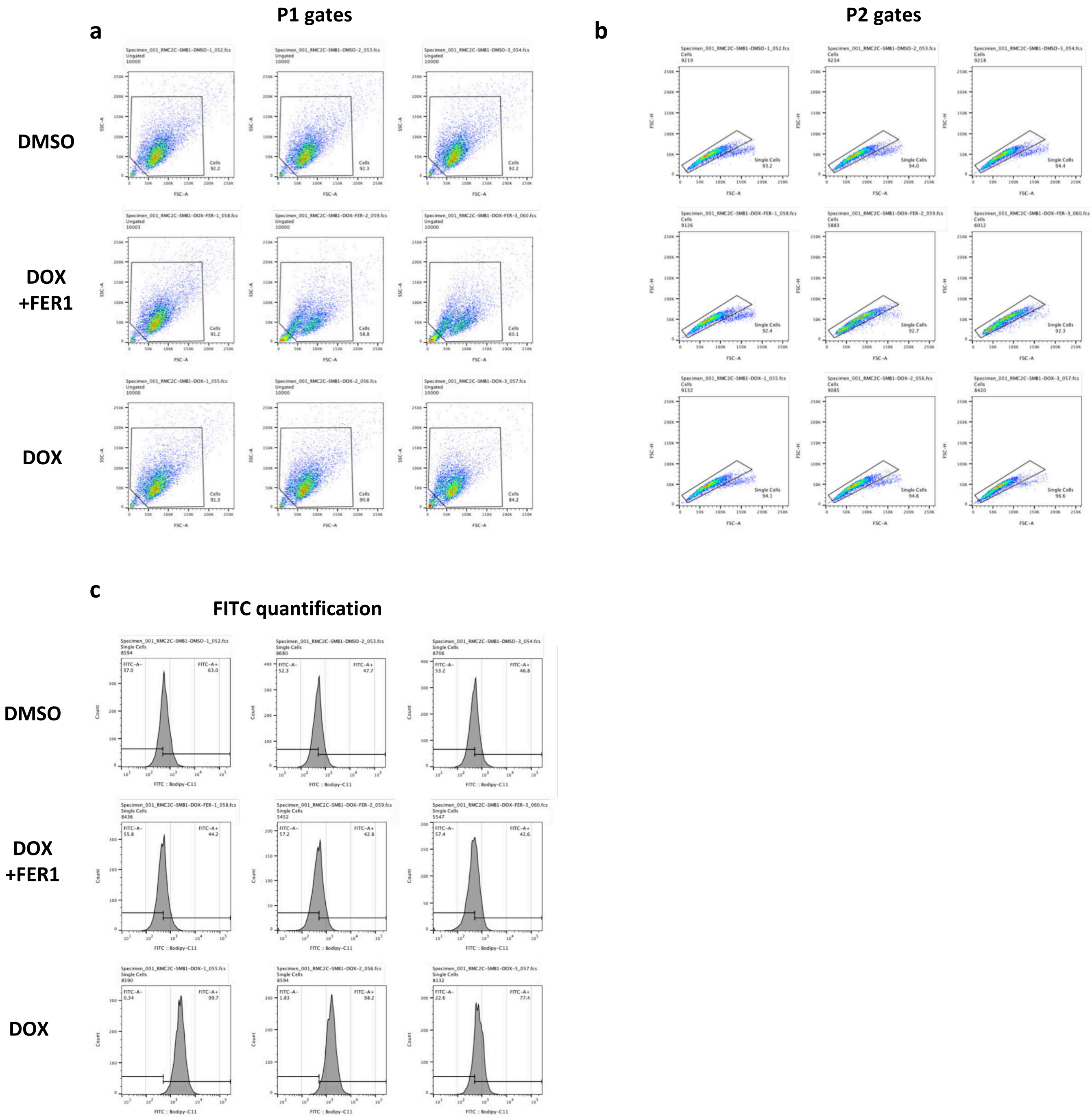

Vokshi et al., Suppl. Fig. 8

Supplementary Figure 8. Representative example of gating strategy for Fig. 5c. $1x10^6$ RMC2C cells were stained with 10uM BODIPY-C11 for 20min before harvesting. After three PBS washes, cells were analyzed on the BD FACS Fortessa using FACSDiva and FlowJo softwares. The gating strategy involved **a.** selecting normal-size viable cells (P1) through FSC-A and SSC-A optical detectors; **b.** filtering P1 cells using FSC-A and FSC-H channels to select single cells (P2) and **c.** measuring oxidized BODIPY-C11 in live P2 cells by FITC fluorescence (510 nm). As lipid peroxidation induces a switch of the BODIPY-C11 fluorescence from red (590nm) to green (510nm), only FITC was quantified relative to DMSO controls. Final results are shown as histograms using BODIPY-C11::FITC on X axis and normalized cell count on Y axis.

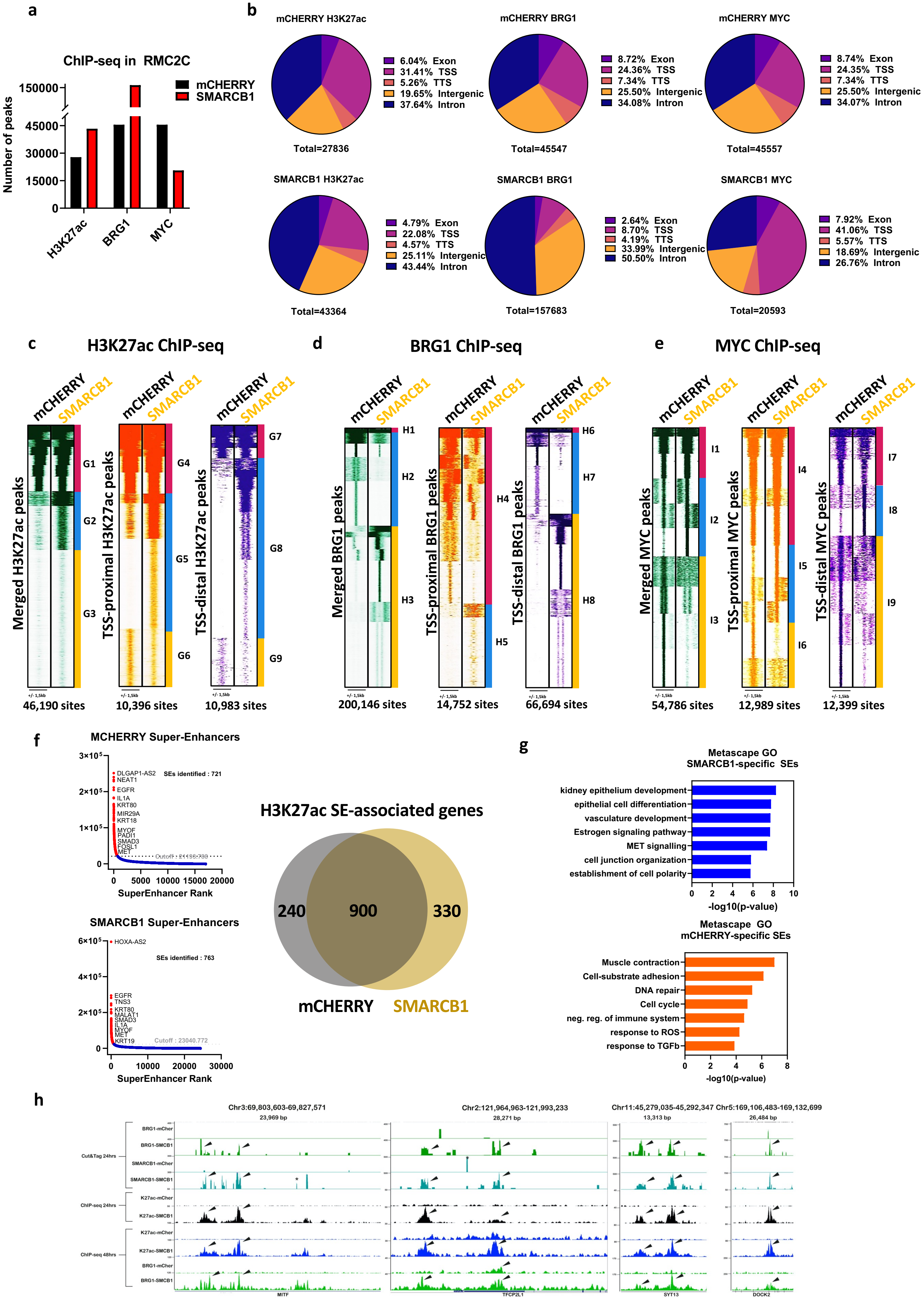

Vokshi et al., Suppl. Fig. 9

Supplementary Figure 9. a. Number of peaks of H3K27ac, BRG1 and MYC in SMARCB1 or mCHERRY-expressing cells as quantified by the MACS algorithm. **b.** Pie charts showing the relative distribution of H3K27ac, BRG1 and MYC peaks on defined genome elements. **c-e**. Read density maps of H3K27ac (C), BRG1 (D), MYC (E) peaks in SMARCB1- or mCHERRY-expressing cells using either all merged, TSS-proximal or TSS-distal sites as a reference. **f.** ROSE identification of H3K27ac Super-Enhancers (SE) in RMC2C cells expressing SMARCB1 or mCHERRY (left), and Venn diagram of shared and specific SE-associated genes (right). **g.** Ontology enrichment analysis of mCHERRY- and SMARCB1-specific SE-associated genes. P-values were calculated by GREAT using a binomial test corrected with Benjamin-Hochberg FDR adjustment. **h**. UCSC genome track snapshots showing the SMARCB1, BRG1 and H3K27ac signals at regulatory elements of selected relevant genes.

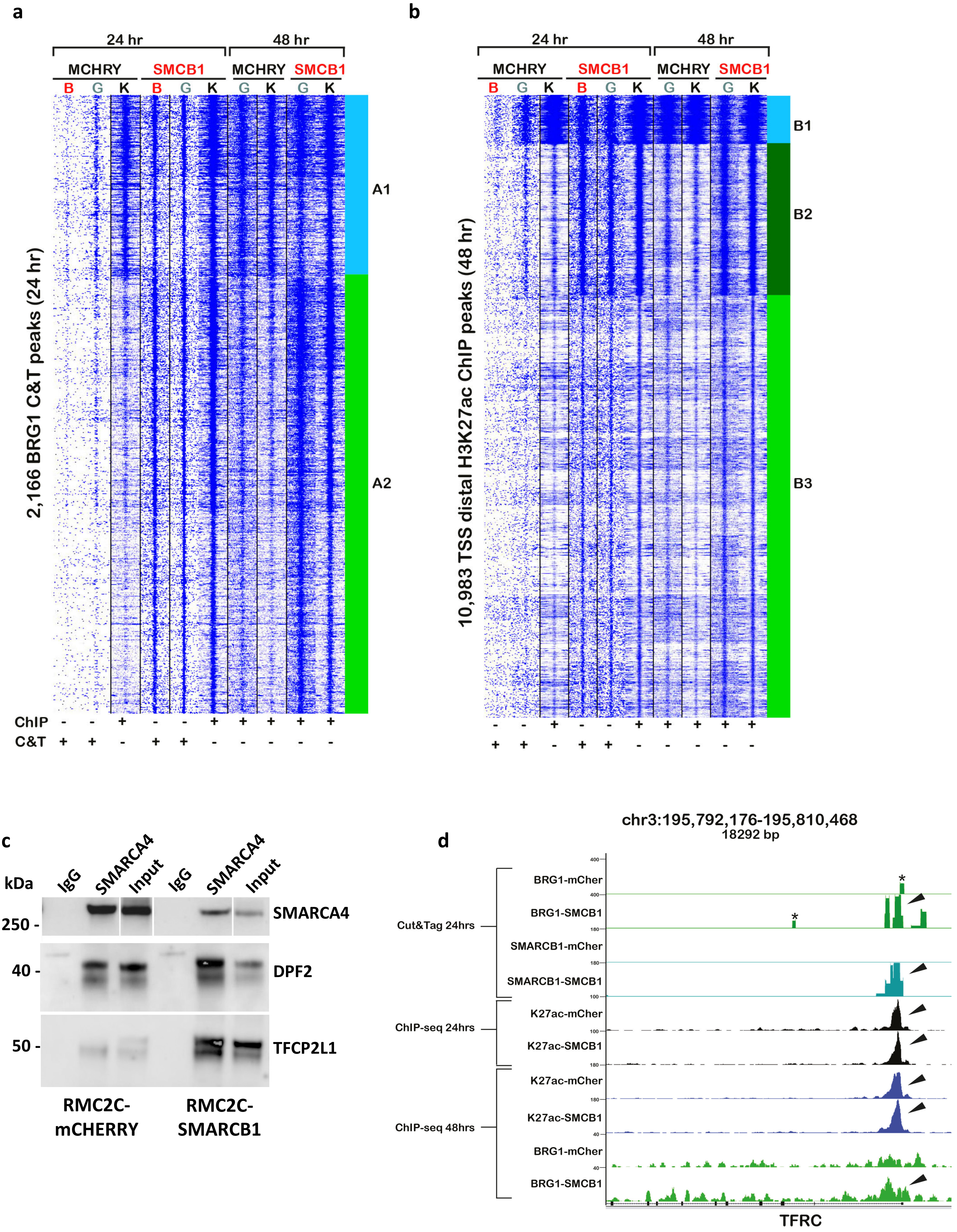

Vokshi et al., Suppl. Fig. 10

Supplementary Figure 10. **a.** Read density heat map of Cut&Tag and H3K27ac ChIP-seq 24 hours after Dox treatment of mCherry (MCHRY) or SMARCB1 (SMCB1) expressing cells. **b**. Read density heat map of Cut&Tag and H3K27ac ChIP-seq 24 hours after Dox treatment at the distal located BRG1 and H3K27ac marked sites 48 hours after Dox treatment. B= SMARCB1; G = BRG1; K = H3K27ac. **c**. Immunoblot showing co-precipitation of TFCP2L1 along with SWI/SNF subunits BRG1 and DPF2. n=2 independent biological replicates. Molecular mass markers in kDa are indicated. **d**. UCSC genome track snapshots showing the SMARCB1, BRG1 and H3K27ac signals at the TFRC promoter region. Source data are provided as a Source Data file 2.

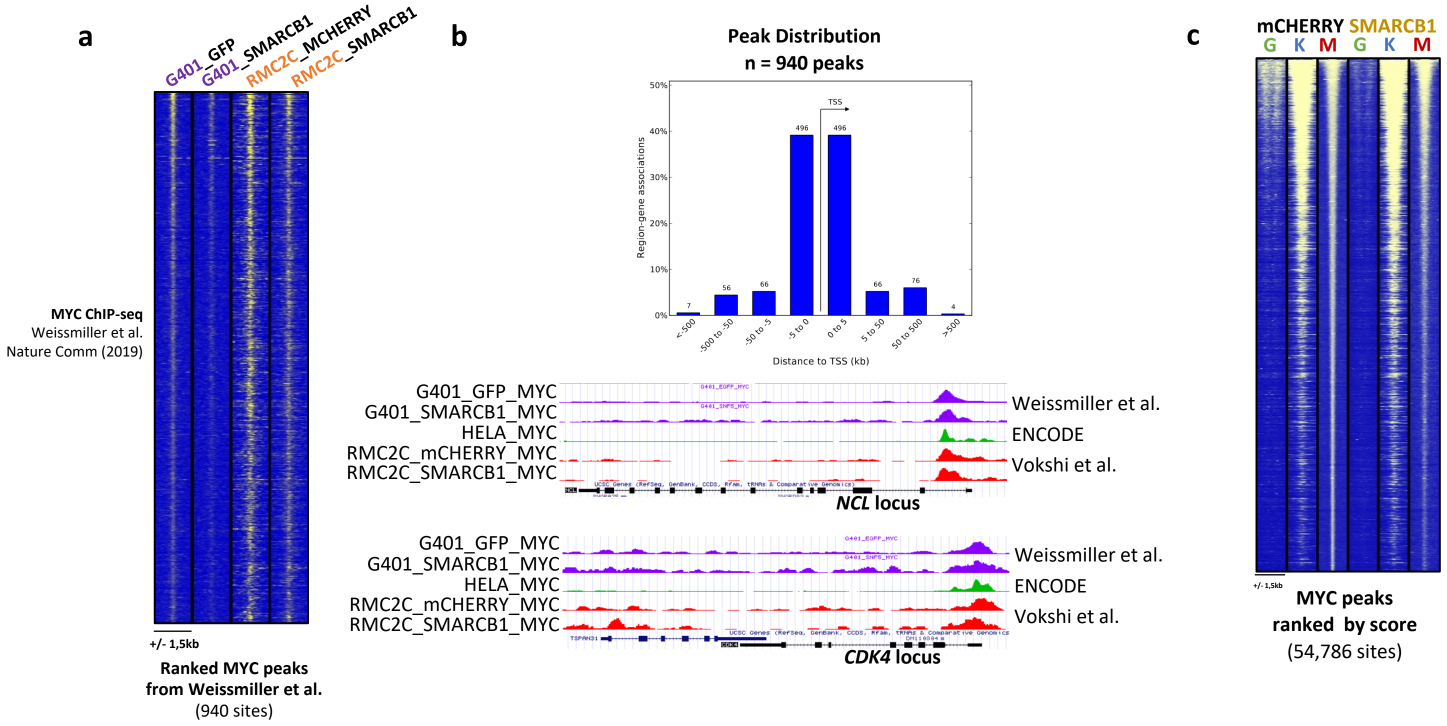

d

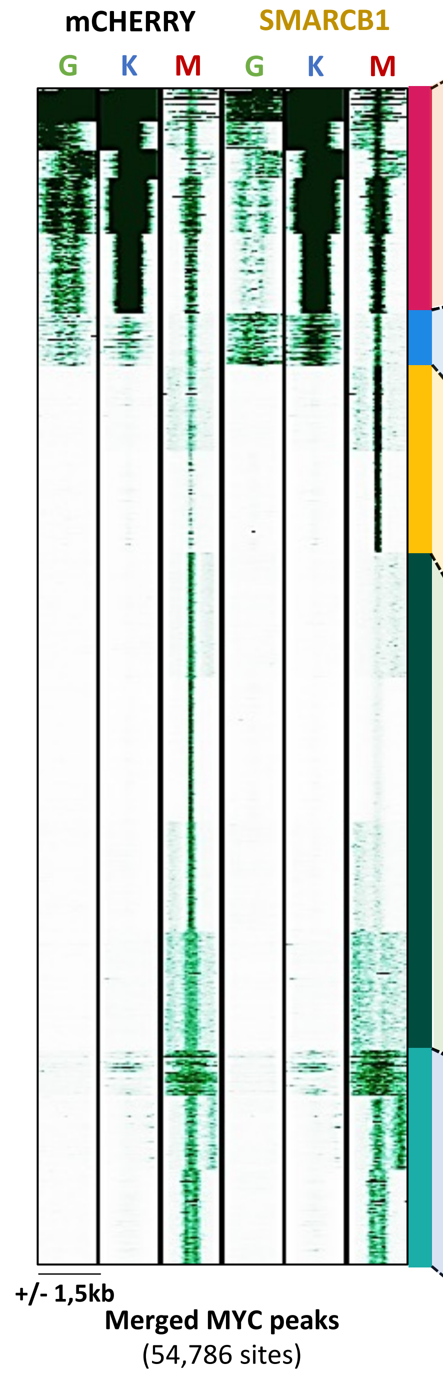

D1

Consensus	Motif name	# sites	E-value
cCACGTGg	MYC::MAX	5896	3E-131
cGCCCCCACCc	MAZ	6215	1,1E-107
gCTGGGCAGGGCA	KLF15	3175	1,9E-94
CGCCCTCG	E2F6	3904	4,8E-78
cACTTCCG	ETS1	1512	5E-81

D2

Consensus	Motif name	# sites	E-value
cCACGTGg	MYC::MAX	1558	8,1E-22
CCCCGCCC	EGR1	872	5,8E-19
CGCCcCC	CTCFL	1546	1,1E-15
TGACTCA	BATF::JUN	772	4,3E-14
CGCCTGC	ZEB1	818	2,8E-13

D3

Consensus	Motif name	# sites	E-value
cCACGTGg	MYC::MAX	3712	1,4E-65
gCTGGGCAGGGCA	KLF15	1662	8,9E-42
CGaGCG	AHR:ARNT	1782	4,7E-36
CGCCcCC	CTCFL	4077	1,5E-33
CCCCGCCC	EGR1	2984	2,1E-30

D4

Consensus	Motif name	# sites	E-value
cCACGTGg	MYC::MAX	8828	9,1E-147
ACGTGc	HIF1A	8436	1,4E-70
AAAATAAAAA	MEF2A	8680	8,1E-67
GGTGTGG	SNAI1	7211	1,2E-65
CGCCCTCG	E2F6	8156	1,3E-53

D5

Consensus	Motif name	# sites	E-value
CGCCCTCG	KLF15	8142	2,6E-121
cGCCCCCACCc	MAZ	3340	1,4E-108
CGCCCCC	CTCF	3135	2,3E-72
cCACGTGg	MYC::MAX	7271	1E-69
CGCCTGC	TCF3	4564	8,1E-63

Vokshi et al., Suppl. Fig. 11

Supplementary Figure 11. **a.** Tornado read density maps comparing MYC occupancy in G401 and RMC2C cells. **b.** Distribution of G401 MYC peaks with respect to the TSS. Examples of MYC binding at the *NCL* and *CDK4* loci. **c.** Tornado read density maps showing BRG1, H3K27ac and MYC sites ranked decreasingly by MYC peak score. **d.** RSAT-based motif enrichment analysis at each of the MYC sub-clusters.

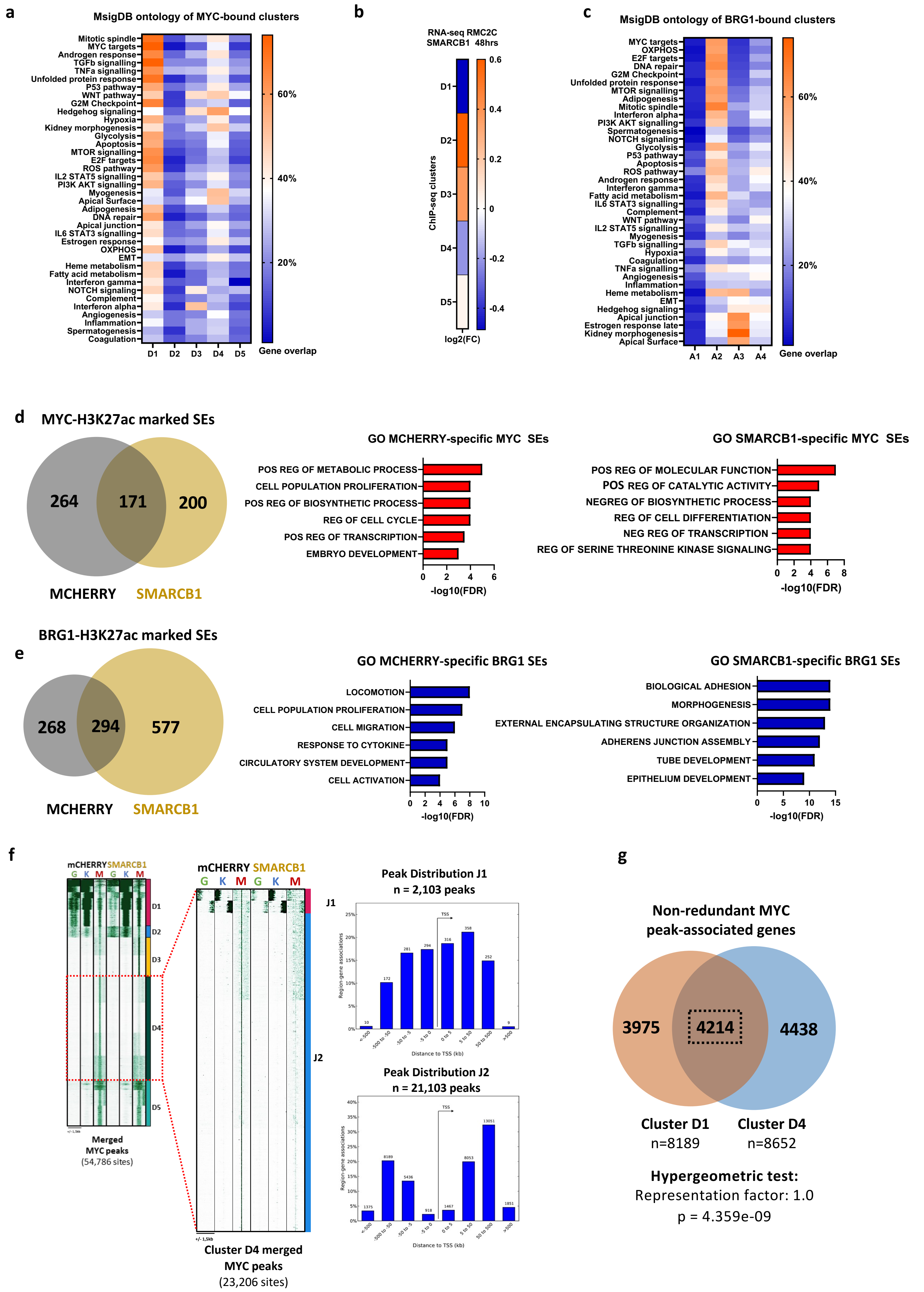

Vokshi et al., Suppl. Fig. 12

Supplementary Figure 12. **a.** Percentage of genes associated with MYC clusters as defined by HOMER in the indicated GSEA Hallmark Genesets. **b.** Changes in expression of genes associated with each MYC cluster upon SMARCB1 expression. **c.** Percentage of genes associated with BRG1 clusters as defined by HOMER in the indicated GSEA Hallmark Genesets. **d-e.** Venn diagram of SE-associated genes defined by MYC peak score (D) or BRG1 peak score (E) revealing common and specific SEs in SMARCB1- and mCHERRY-expressing RMC2C cells (left) and associated ontology analysis of SMARCB1- and mCHERRY-specific SE-associated genes (right). **f.** Read density maps showing sub-clustering of MYC D4 sites, with their peak distribution as calculated by GREAT (middle), and the associated ontology analysis of associated genes. **g.** Venn diagram of non-redundant MYC-bound genes found in clusters D1 and D4. P-values were calculated using a two-tailed hypergeometric test.

Supplementary Dataset 1. Markers defining cell clusters of the treated tumour, naive tumour and PDX (IC-pPDX-132) cells as calculated by Seurat FindMarkers algorithm. P-values were calculated using the non-parametric Wilcoxon rank sum test corrected with Bonferroni FDR adjustment.

Supplementary Dataset 2. Bulk RNA-seq of NAT, RMC primary tumours and lymph node metastases (n=44) from the MDACC cohort.

Supplementary Dataset 3. RNA-seq of RMC2C and RMC219 cells at 12hrs and 48hrs after SMARCB1 re-expression. P-values were calculated using the Wald test corrected with Benjamin-Hochberg FDR adjustment.

Supplementary Table 1. List of qPCR primers

Targets	Forward Sequence	Reverse Sequence
CD274	GGCATCCAAGATACAAACTCAA	CAGAAGTTCCAATGCTGGATTA
STAT1	TGAGTTGATTTCTGTGTCTGAAGTT	ACACCTCGTCAAACTCCTCAG
IRF1	GGCACATCCCAGTGGAAG	CCCTTCCTCATCCTCATCTGT
ACTB	CCAACCGCGAGAAGATGA	CCAGAGGCGTACAGGGATAG
GAPDH	CCCCGGTTTCTATAAATTGAGC	CTTCCCCATGGTGTCTGAG
RPL13A	AACCTCCTCCTTTTCCAAGC	AGCGTACGACCACCACCTT
TBP	CGGCTGTTTAACTTCGCTTC	CACACGCCAAGAAACAGTGA
SMARCB1	GCGCTGAGCAAGACCTTC	CCTCGGAACATACGGAGGTA

Supplementary Table 2. List of antibodies and compounds used

Targets	Manufacturers	References
CDH1	CST	3195
CLDN1	Abcam	15098
MITF	Interchim	MS-771-P
VIM	CST	5741
SLUG	CST	9585
PDL1	CST	13684
MYC	SCT	sc-40
NFE2L2	Abcam	62352
TFCP2L1	Sigma	HPA029708
SMARCB1	CST	91735
VCL	Sigma	V4505
FN1	Sigma	F3648
GPX4	R&D BioTechne	5457-SP
ACSL4	ThermoFisher	PA5-89830
HA	Sigma	H6908
SMARCA4	Abcam	110641
SMARCA2	CST	11966
SMARCC1	Bethyl Lab	A301-038A
SMARCC2	SCT	sc10756
SMARCD1	BD Transduction labs	611728
SMARCD2	Abcam	166622
SMARCD3	CST	622665
SMARCE1	BL	A300-810A
ACTL6A	Abcam	131272
ACTB	Inhouse (IGBMC)	2D7
BCL7A	Invitrogen	PA5-27123
BCL7B	SCT	sc-134278
ARID1A	CST	12354
ARID1B	CST	92964
PBRM1	Merck	ABE70
ARID2	Abcam	166850
BRD7	Abcam	56036
DPF1	ThermoFisher	PA5-61895
DPF2	Abcam	134942
DPF3	ThermoFisher	PA5-38011
ZEB1	CST	3396
JUN	CST	9165
TFRC	Invitrogen	13-6800
4-HNE	Abcam	HNEJ-2-ab48506

AlexaFluor-488	Invitrogen	goat anti mouse # A11001
AlexaFluor-488	Invitrogen	Goat against Rabbit: 111-035-144
HRP Coupled	Jackson ImmunoResearch	Goat against Mouse: 115-036-71
HRP Coupled	Jackson ImmunoResearch	Goat against Rabbit: 111-035-144
**Compounds**		
Ferrostatin-1	SelleckChem	#S7243
zVAD-fmk	MedChemExpress	#HY-16658B
Necrostatin-1	MedChemExpress	#HY-15760
Camptothecin	SelleckChem	#S1288
IFNg	Peprotech	300-02
Bodipy 581/591 C11	ThermoFisher	#D3861
Bafilomycin A1	Sigma	#19-148
C12FDG	Invitrogen	#D2893
RSL3	SelleckChem	8155
Doxycycline	Sigma	#D9891
