# Supplementary material for: SMARCB1 regulates a TFCP2L1-MYC transcriptional switch promoting renal medullary carcinoma transformation and ferroptosis resistance
Source: Nat Commun. 2023 May 26;14:3034. doi: 10.1038/s41467-023-38472-y (PMC10220073; doi:10.1038/s41467-023-38472-y)
Supplement: Supplementary file 2 — Description of Additional Supplementary Files [file 41467_2023_38472_MOESM2_ESM.pdf]

Description of Additional Supplementary Files

File Name: Supplementary Data 1
Description: Markers defining cell clusters of the treated tumour, naive tumour and PDX (IC-pPDX-132) cells as calculated by Seurat FindMarkers algorithm. P-values were calculated using the non-parametric Wilcoxon rank sum test corrected with Bonferroni FDR adjustment.

File Name: Supplementary Data 2
Description: Bulk RNA-seq of NAT, RMC primary tumours and lymph node metastases (n=44) from the MDACC cohort.

File Name: Supplementary Data 3
Description: RNA-seq of RMC2C and RMC219 cells at 12hrs and 48hrs after SMARCB1 re-expression. P-values were calculated using the Wald test corrected with Benjamin-Hochberg FDR adjustment.
