# Supplementary material for: SMARCB1 regulates a TFCP2L1-MYC transcriptional switch promoting renal medullary carcinoma transformation and ferroptosis resistance
Source: Nat Commun. 2023 May 26;14:3034. doi: 10.1038/s41467-023-38472-y (PMC10220073; doi:10.1038/s41467-023-38472-y)
Supplement: Supplementary file 6 — Reporting Summary [file 41467_2023_38472_MOESM6_ESM.pdf]

nature portfolio

Corresponding author(s): Gabriel Malouf

Last updated by author(s): 30/03/2023

Reporting Summary

Nature Portfolio wishes to improve the reproducibility of the work that we publish. This form provides structure for consistency and transparency in reporting. For further information on Nature Portfolio policies, see our Editorial Policies and the Editorial Policy Checklist.

Statistics

For all statistical analyses, confirm that the following items are present in the figure legend, table legend, main text, or Methods section.

n/a	Confirmed	
☐	☒	The exact sample size (n) for each experimental group/condition, given as a discrete number and unit of measurement
☐	☒	A statement on whether measurements were taken from distinct samples or whether the same sample was measured repeatedly
☐	☒	The statistical test(s) used AND whether they are one- or two-sided<br>*Only common tests should be described solely by name; describe more complex techniques in the Methods section.*
☐	☒	A description of all covariates tested
☐	☒	A description of any assumptions or corrections, such as tests of normality and adjustment for multiple comparisons
☐	☒	A full description of the statistical parameters including central tendency (e.g. means) or other basic estimates (e.g. regression coefficient) AND variation (e.g. standard deviation) or associated estimates of uncertainty (e.g. confidence intervals)
☐	☒	For null hypothesis testing, the test statistic (e.g. F, t, r) with confidence intervals, effect sizes, degrees of freedom and P value noted<br>*Give P values as exact values whenever suitable.*
☒	☐	For Bayesian analysis, information on the choice of priors and Markov chain Monte Carlo settings
☐	☒	For hierarchical and complex designs, identification of the appropriate level for tests and full reporting of outcomes
☐	☒	Estimates of effect sizes (e.g. Cohen's d, Pearson's r), indicating how they were calculated

Our web collection on statistics for biologists contains articles on many of the points above.

Software and code

Policy information about availability of computer code

Data collection	no software was used
Data analysis	CellRanger (v 3.1), R (v4.0.2), Seurat v3.2.0, SCENIC v1.1.2.2, Similarity Weighted Nonnegative Embedding (SWNE), r-package GSVA, R package VISION V2.2.0, Flowjo v6.8, STAR version 2.5.3a, Bioconductor package DESeq2 version 1.16.1,MCP-counter v1.2.0, CIBERSORTx algorithm, Bowtie, MACS, seqMINER, ROSE, MEME Suite, RSAT, AUC V1.8.0

For manuscripts utilizing custom algorithms or software that are central to the research but not yet described in published literature, software must be made available to editors and reviewers. We strongly encourage code deposition in a community repository (e.g. GitHub). See the Nature Portfolio guidelines for submitting code & software for further information.

Data

Policy information about availability of data

All manuscripts must include a data availability statement. This statement should provide the following information, where applicable:

- Accession codes, unique identifiers, or web links for publicly available datasets
- A description of any restrictions on data availability
- For clinical datasets or third party data, please ensure that the statement adheres to our policy

The data sets described here have been deposited at GEO with the accession number GSE181001

Human research participants

Policy information about studies involving human research participants and Sex and Gender in Research.

Reporting on sex and gender	RMC from two patients were analyzed, one male and one female.
Population characteristics	1 male patient aged 16 years old with regional lymph node and adrenal gland metastases (pT4N1M1) at presentation and 1 female aged 21 with lung metastases at diagnosis
Recruitment	Due to the rarity of the tumour we retrospectively analysed the only two tumours with fresh material available that were available. There was no prospective recruitment.
Ethics oversight	The two RMC samples subjected to scRNA-seq were collected from Strasbourg University Hospital and Curie Institute, according to institutional guidelines. Sample collection for further research analysis was approved ethical Committees of Strasbourg University Hospital and Curie Institute and all patients provided an informed written consent for the use of material for further research.

Note that full information on the approval of the study protocol must also be provided in the manuscript.

Field-specific reporting

Please select the one below that is the best fit for your research. If you are not sure, read the appropriate sections before making your selection.

☒ Life sciences ☐ Behavioural & social sciences ☐ Ecological, evolutionary & environmental sciences

For a reference copy of the document with all sections, see nature.com/documents/nr-reporting-summary-flat.pdf

Life sciences study design

All studies must disclose on these points even when the disclosure is negative.

Sample size	Two human tumour samples were used for scRNA-seq as well as one PDX sample. No a priori calculation of sample size was done as experiments were performed based on the limited availability of access to human tumour samples. For all other cell based experiments excluding ChIP-seq and Cut&Tag a N= minimum of 3 biological replicates were used to generate data. Data from each given experimental condition showed no significant differences indicating a sufficient sample size.
Data exclusions	None
Replication	All attempts at replications were successful
Randomization	Not relevant for this study where each sample was subjected to a specific experimental procedure.
Blinding	Blinding was not possible as the data were acquired and analyzed by the same person that performed the experimental procedure

Reporting for specific materials, systems and methods

We require information from authors about some types of materials, experimental systems and methods used in many studies. Here, indicate whether each material, system or method listed is relevant to your study. If you are not sure if a list item applies to your research, read the appropriate section before selecting a response.

Materials & experimental systems

n/a	Involved in the study	
☐	☒	Antibodies
☐	☒	Eukaryotic cell lines
☒	☐	Palaeontology and archaeology
☐	☒	Animals and other organisms
☐	☒	Clinical data
☒	☐	Dual use research of concern

Methods

n/a	Involved in the study	
☐	☒	ChIP-seq
☐	☒	Flow cytometry
☒	☐	MRI-based neuroimaging

Antibodies

Antibodies used

CDH1 CST 3195
CLDN1 Abcam 15098
MITF Interchim MS-771-P
VIM CST 5741
SLUG CST 9585
PDL1 CST 13684
MYC SCT sc-40
NFE2L2 Abcam 62352
TFCP2L1 Sigma HPA029708
SMARCB1 CST 91735
VCL Sigma V4505
FN1 Sigma F3648
GPX4 R&D BioTechne 5457-SP. Clone # 565320
ACSL4 ThermoFisher PA5-89830
HA Sigma H6908
SMARCA4 Abcam 110641
SMARCA2 CST 11966
SMARCC1 Bethyl Lab A301-038A
SMARCC2 SCT sc10756
SMARCD1 BD Transduction labs 611728
SMARCD2 Abcam 166622
SMARCD3 CST 622665
SMARCE1 BL A300-810A
ACTL6A Abcam 131272
ACTB Inhouse (IGBMC) 2D7
BCL7A Invitrogen PA5-27123
BCL7B SCT sc-134278
ARID1A CST 12354
ARID1B CST 92964
PBRM1 Merck ABE70
ARID2 Abcam 166850
BRD7 Abcam 56036
DPF1 ThermoFisher PA5-61895
DPF2 Abcam 134942
DPF3 ThermoFisher PA5-38011
ZEB1 CST 3396
JUN CST 9165
TFRC Invitrogen 13-6800
4HNE. HNEJ-2-ab48506. 1/5000
AlexaFluor-488 Invitrogen goat anti mouse # A11001 and goat anti-rabbit # A32731 1/500
Jackson ImmunoResearch; Goat against Mouse: 115-036-71; Goat against Rabbit: 111-035-144 dilution 1.2000

Validation

https://www.cellsignal.com/products/primary-antibodies/e-cadherin-24e10-rabbit-mab/3195
https://www.abcam.com/claudin-1-antibody-ab15098.html
https://www.fishersci.fr/shop/products/microphthalmia-transcription-factor-mitf-ab-1-mouse-monoclonal-antibody/p-4552643
https://www.cellsignal.com/products/primary-antibodies/vimentin-d21h3-xp-rabbit-mab/5741
https://www.cellsignal.com/products/primary-antibodies/slug-c19g7-rabbit-mab/9585
https://www.cellsignal.com/products/primary-antibodies/pd-l1-e1l3n-xp-rabbit-mab/13684
https://www.clinisciences.com/autres-produits-186/c-myc-9e10-ac-1000071.html
https://www.abcam.com/nrf2-antibody-ep1808y-chip-grade-ab62352.html
https://www.sigmaaldrich.com/FR/fr/product/sigma/hpa029708
https://www.cellsignal.com/products/primary-antibodies/smarcb1-baf47-d8m1x-rabbit-mab/91735
https://www.sigmaaldrich.com/FR/fr/product/sigma/v4505
https://www.sigmaaldrich.com/FR/fr/product/sigma/f3648
https://www.rndsystems.com/products/human-mouse-rat-glutathione-peroxidase-4-gpx4-antibody-565320_mab5457
https://www.thermofisher.com/antibody/product/ASCL4-Antibody-Polyclonal/PA5-89830
https://www.sigmaaldrich.com/FR/fr/product/sigma/h6908
https://www.abcam.com/brg1-antibody-epncir111a-ab110641.html
https://www.cellsignal.com/products/primary-antibodies/brm-d9e8b-xp-rabbit-mab/11966
https://yris.ozyme.fr/fr/company/ozyme/product/rabbit-anti-smarcc2-baf170-ab-affinity-purified-beta301-038a-1
https://www.labome.com/product/BD-Biosciences/611728.html
https://www.abcam.com/smarcd2-antibody-epr20860-251-ab220164.html
https://www.sigmaaldrich.com/FR/fr/product/sigma/av35652?
gclid=Cj0KCQjw0oyYBhDGARIsAMZEuMsdjq1MYmDFoo2MhW_dP6O5ZjBiRyKVMIkCSoFubIFF2vWDbtr3y0waAk2GEALw_wcB
https://www.thermofisher.com/antibody/product/BAF57-SMARCE1-Antibody-Polyclonal/A300-810A
https://www.abcam.com/actl6a-antibody-epr7443-ab131272.html
https://www.thermofisher.com/antibody/product/BCL7A-Antibody-Polyclonal/PA5-27123
https://www.scbt.com/p/bcl-7b-antibody-nq-b25
https://www.cellsignal.com/products/primary-antibodies/arid1a-baf250a-d2a8u-rabbit-mab/12354
https://www.cellsignal.com/products/primary-antibodies/arid1b-baf250b-e9j4t-rabbit-mab/92964
https://www.merckmillipore.com/FR/fr/product/Anti-BAF180-Antibody,MM_NF-ABE70?ReferrerURL=https%3A%2F%

2Fwww.google.com%2F
https://www.abcam.com/arid2-antibody-ab166850.html
https://www.abcam.com/brd7-antibody-2d3-bsa-and-azide-free-ab255776.html
https://www.thermofisher.com/antibody/product/DPF1-Antibody-Polyclonal/PA5-61895
https://www.abcam.com/dpf2req-antibody-epr9206b-ab134942.html
https://www.thermofisher.com/antibody/product/DPF3-Antibody-Polyclonal/PA5-38011
https://www.cellsignal.com/products/primary-antibodies/zeb1-d80d3-rabbit-mab/3396
https://www.cellsignal.com/products/primary-antibodies/c-jun-60a8-rabbit-mab/9165
https://www.thermofisher.com/antibody/product/Transferrin-Receptor-Antibody-clone-H68-4-Monoclonal/13-6800
https://www.abcam.com/products/primary-antibodies/4-hydroxynonenal-antibody-hnej-2-ab48506.html
https://www.thermofisher.com/antibody/product/Goat-anti-Mouse-IgG-H-L-Cross-Adsorbed-Secondary-Antibody-Polyclonal/A-11001
https://www.thermofisher.com/antibody/product/Goat-anti-Rabbit-IgG-H-L-Highly-Cross-Adsorbed-Secondary-Antibody-Polyclonal/A32731
https://www.jacksonimmuno.com/catalog/products/115-036-071
https://www.jacksonimmuno.com/catalog/products/111-035-144

Eukaryotic cell lines

Policy information about cell lines and Sex and Gender in Research

Cell line source(s)	RMC-2C was a kind gift from Dr Nizar Tannir (MDACC.) RMC-219 was a kind gift from Dr James Hsieh (MSKCC). UOK353 and UOK360 were a kind gift from Dr Martson Linehan (NCI). None of these cell lines are commercially available. HEK293T cells were obtained from ATCC.
Authentication	Cell lines were authenticated in this study by immunoblot showing absence of SMARCB1 expression and by RNA-seq
Mycoplasma contamination	All cell lines were regularly tested as negative for Mycoplasma infection using the Venor™ GeM Mycoplasma Detection Kit, and used at less than 10 passages
Commonly misidentified lines (See ICLAC register)	No lines of this category were used

Animals and other research organisms

Policy information about studies involving animals; ARRIVE guidelines recommended for reporting animal research, and Sex and Gender in Research

Laboratory animals	Animal care and use for this study were performed in accordance with the recommendations of the European Community (2010/63/UE) for the care and use of laboratory animals and carried out in accordance with the principles of the Declaration of Helsinki and with GDPR regulations. The experiments were approved by the Curie Institute animal ethical committee CEEA-IC #118 (Authorization APAFIS#11206-2017090816044613-v2 given by National Authority) and performed in accordance with the internal, national and European guidelines of Animal Care and Use. Mice were maintained in IVC cages in a semi pathogen-free facility under standard housing conditions with continuous access to food and water. Curie Institute animal facilities comply with all appropriate standards (cages, space per animal, temperature (22 °C), light, 12 hour light/dark cycle, 50% humidity, continuous access to food and water), and all cages are enriched with nesting materials. The establishment of PDX received approval by the Institut Curie institutional review board OBS170323 CPP ref 3272; n de dossier 2015- A00464-45). Written institutional informed consent was obtained from the patient.
Wild animals	None
Reporting on sex	One female mouse was used to propagate the analyszed human PDX.
Field-collected samples	None
Ethics oversight	The experiments were approved by the Curie Institute animal ethical committee CEEA-IC #118 (Authorization APAFIS#11206-2017090816044613-v2 given by National Authority) and performed in accordance with the internal, national and European guidelines of Animal Care and Use. The establishment of PDX received approval by the Institut Curie institutional review board OBS170323 CPP ref 3272; n de dossier 2015- A00464-45). Written institutional informed consent was obtained from the patient.

Note that full information on the approval of the study protocol must also be provided in the manuscript.

Clinical data

Policy information about clinical studies
All manuscripts should comply with the ICMJE guidelines for publication of clinical research and a completed CONSORT checklist must be included with all submissions.

Clinical trial registration	not relevant to the study

Study protocol
not relevant to the study

Data collection
de-identified data was collected from medical records

Outcomes
not relevant to the study

ChIP-seq

Data deposition

☒ Confirm that both raw and final processed data have been deposited in a public database such as GEO.

☒ Confirm that you have deposited or provided access to graph files (e.g. BED files) for the called peaks.

Data access links
May remain private before publication.

The data sets described here have been deposited at GEO with the accession number GSE181001

Files in database submission

ChIP-seq
H3K27ac: RMC2C-mCherry. RMC2C-SMARCB1
BRG1: RM2C2-mCherry. RM2C2-SMARCB1.
MYC; RM2C2 mCherry. RMC2C SMARCB1.
H3K27ac 24 hours: RMC2C-mCherry. RMC2C-SMARCB1
Cut&Tag.
BRG1: RMC2C-mCherry. RMC2C-SMARCB1
SMARCB1 RMC2C-mCherry. RMC2C-SMARCB1

scRNA-seq
RMC Treated Tumor
RMC NAT
RMC PDX
RMC Naive Tumor

Bulk RNA-seq
RMC219_NEG_1
RMC219_NEG_2
RMC219_NEG_3
RMC219_12hr_1
RMC219_12hr_2
RMC219_12hr_3
RMC219_48hr_1
RMC219_48hr_2
RMC219_48hr_3
RMC2C_NEG_1
RMC2C_NEG_2
RMC2C_NEG_3
RMC2C_12hr_1
RMC2C_12hr_2
RMC2C_12hr_3
RMC2C_48hr_1
RMC2C_48hr_2
RMC2C_48hr_3

Genome browser session
(e.g. UCSC)

http://genome-euro.ucsc.edu/cgi-bin/hgTracks?db=hg19&lastVirtModeType=default&lastVirtModeExtraState=&virtModeType=default&virtMode=0&nonVirtPosition=&position=chr2%3A177877349%2D178347541&hgsid=290080315_51mrY24Eh49dVbzJSOXCsOYpd5Pv

Methodology

Replicates
1

Sequencing depth

All ChIP-seq 1X 50bp reads.
H3K27ac:
RMC2C-mCherry. Total 45,651,238 Unique 39,272,579
RMC2C-SMARCB1 Total. 48,495,748 Unique 42,341,613
BRG1
RM2C2-mCherry. Total 34,343,622 Unique 28,448,574
RM2C2-SMARCB1. Total 31,938,486 Unique 24,598,565
MYC
RM2C2 mCherry. Total 35,323,012 Unique 26,869,365
RMC2C SMARCB1. Total 35,665,559 Unique 24,356,818

H3K27ac 24 hours.
RMC2C-mCherry. Total 42,507,612 Unique 34 006 089

RMC2C-SMARCB1 Total 46,343,953 Unique 37 075 162

Cut&Tag
All samples Paired end 100 bp reads
BRG1
RMC2C-mCherry . Total mapped 10,797,516
RMC2C-SMARCB1 Total mapped 28,276,285
SMARCB1
RMC2C-mCherry . Total mapped. 7,116,186
RMC2C-SMARCB1 Total mapped 41,562,322

Antibodies

MYC SCT sc-40; SMARCA4(BRG1) Abcam 110641; H3K27ac Abcam 4729; SMARCB1 CST 91735

Peak calling parameters

Sequenced reads were mapped to the Homo sapiens genome assembly hg19 using Bowtie with the following arguments: -m 1 --strata --best -y -S -l 40 -p 2.
Peak calling with MACS. Parameters: -q 0.01 --broad --nomodel --extsize 151

Data quality

Peaks with <FDR 1%: Myc 45557 and 20593 peaks , BRG1, 45547 and 157683, H3K37ac 32836 and 43364 summarized in Fig. S8b.

Software

Sequenced reads were mapped to the Homo sapiens genome assembly hg19 using Bowtie with the following arguments: -m 1 --strata --best -y -S -l 40 -p 2. After sequencing, peak detection was performed using the MACS software

Flow Cytometry

Plots

Confirm that:

☒ The axis labels state the marker and fluorochrome used (e.g. CD4-FITC).

☒ The axis scales are clearly visible. Include numbers along axes only for bottom left plot of group (a 'group' is an analysis of identical markers).

☒ All plots are contour plots with outliers or pseudocolor plots.

☒ A numerical value for number of cells or percentage (with statistics) is provided.

Methodology

Sample preparation

Cells were grown and treated as described in each experiment and cultured for the indicated times before harvesting and flow cytometry.

Instrument

LSRII Fortessa (BD Biosciences)

Software

Flowjo software v 6.8.

Cell population abundance

Cell population abundance was determined using the indicated gating strategies

Gating strategy

Main Fig. 3c and Fig.S4b, cells were gated on CD44 and EPCAM; Fig.4e and 5C, g and h; cells were gated using BODIPY-C11, FITC Annexin V or FITC activated caspase 3 as indicated. A representative example of gating strategy is shown in Fig. S7.

☒ Tick this box to confirm that a figure exemplifying the gating strategy is provided in the Supplementary Information.
