# Supplementary material for: SMARCB1 regulates a TFCP2L1-MYC transcriptional switch promoting renal medullary carcinoma transformation and ferroptosis resistance
Source: Nat Commun. 2023 May 26;14:3034. doi: 10.1038/s41467-023-38472-y (PMC10220073; doi:10.1038/s41467-023-38472-y)
Supplement: Supplementary file 7 — Source Data [file 41467_2023_38472_MOESM7_ESM.zip › Source Data/Source data-Figures.pptx]

## Slide 1
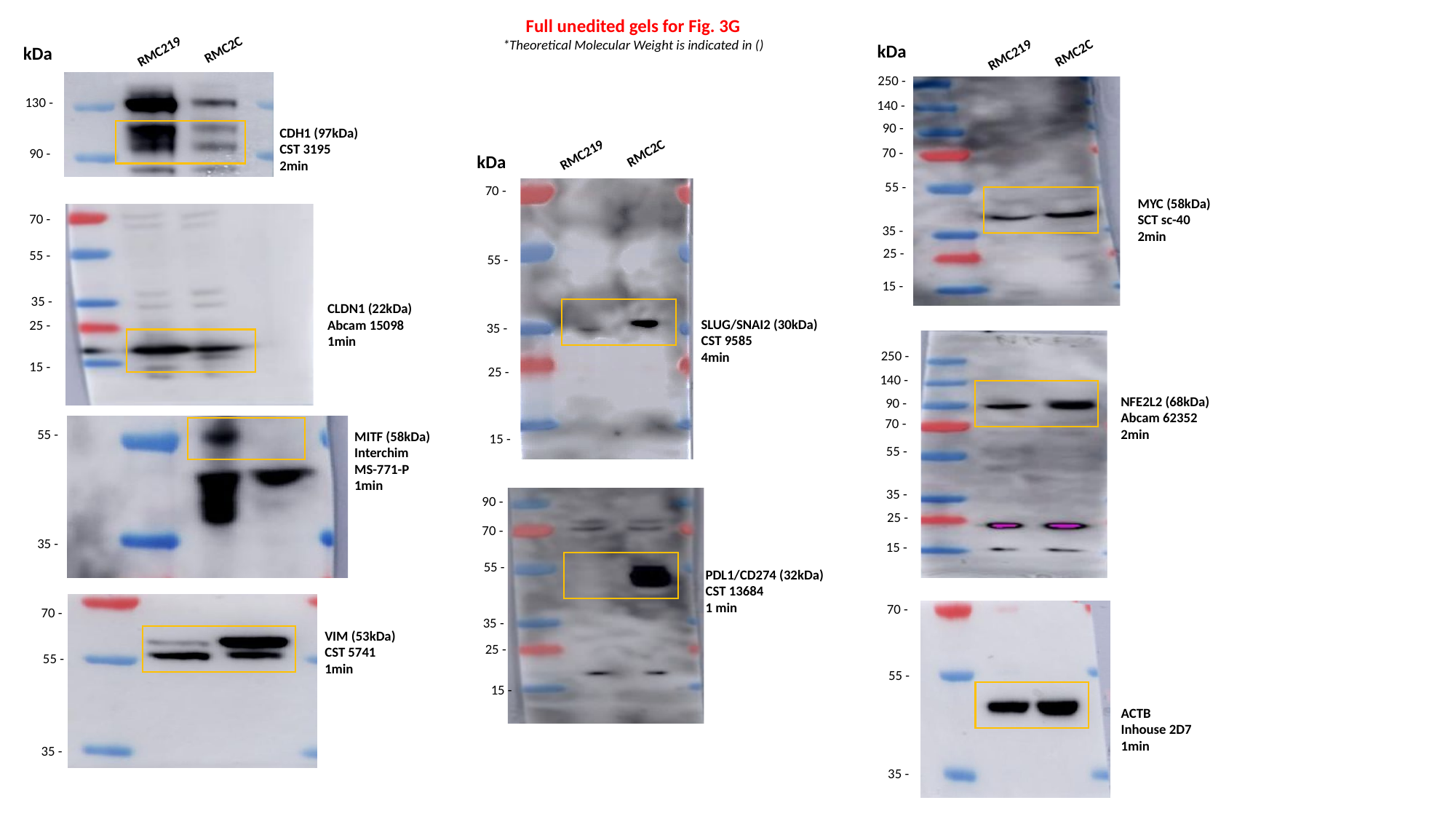

RMC2C
RMC2C
Full unedited gels for Fig. 3G
*Theoretical Molecular Weight is indicated in ()
RMC219
RMC219
kDa
kDa
250 -
140 -
90 -
70 -
55 -
MYC (58kDa)
SCT sc-40
2min
35 -
25 -
15 -
130 -
RMC2C
RMC219
CDH1 (97kDa)
CST 3195
2min
90 -
kDa
70 -
55 -
SLUG/SNAI2 (30kDa)
CST 9585
4min
35 -
25 -
15 -
70 -
55 -
35 -
CLDN1 (22kDa)
Abcam 15098
1min
25 -
15 -
250 -
140 -
NFE2L2 (68kDa)
Abcam 62352
2min
90 -
70 -
55 -
MITF (58kDa)
Interchim
MS-771-P
1min
35 -
55 -
35 -
90 -
70 -
55 -
PDL1/CD274 (32kDa)
CST 13684
1 min
35 -
25 -
15 -
25 -
15 -
70 -
70 -
VIM (53kDa)
CST 5741
1min
55 -
35 -
55 -
ACTB
Inhouse 2D7
1min
35 -

## Slide 2
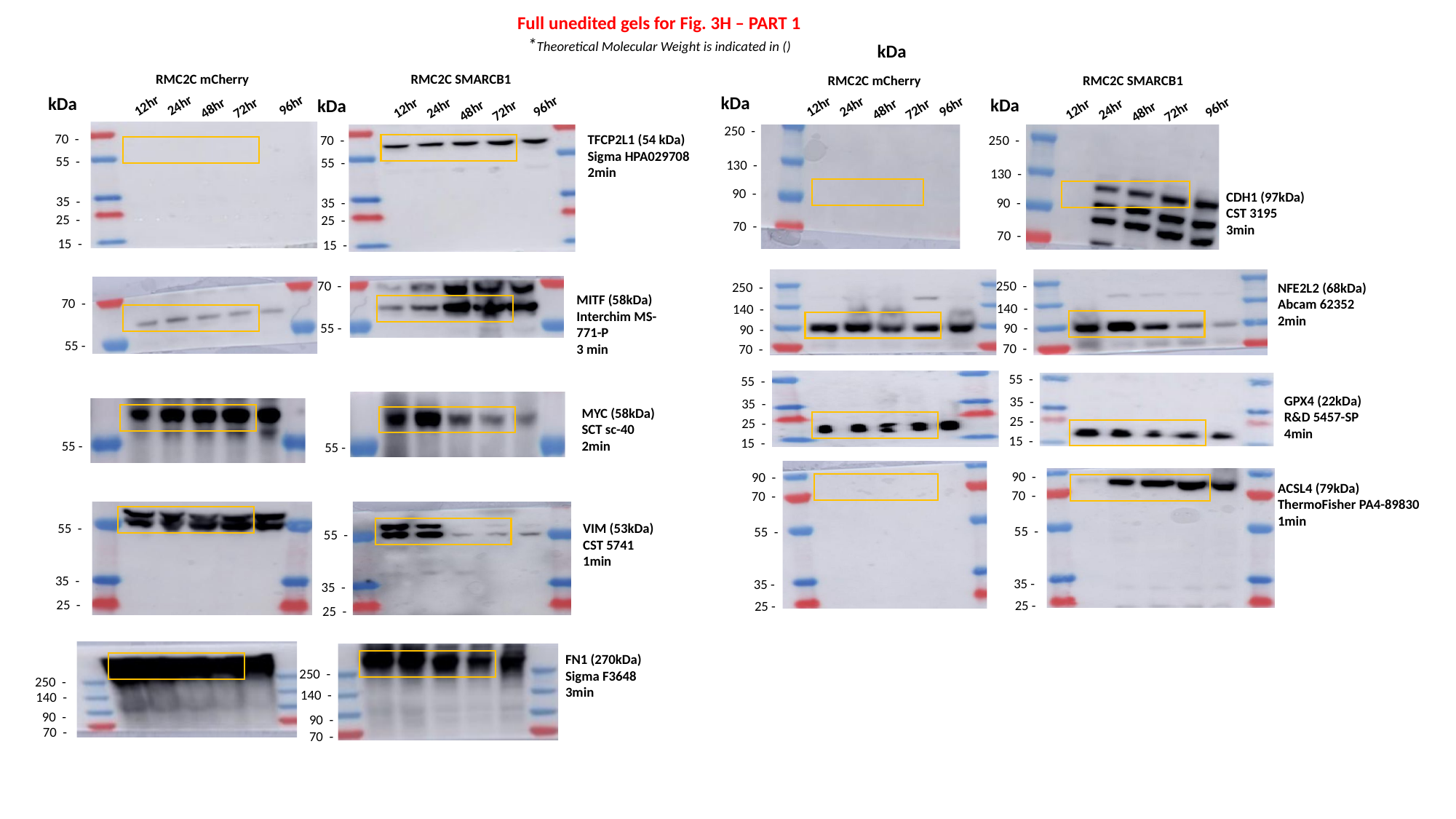

Full unedited gels for Fig. 3H – PART 1
*Theoretical Molecular Weight is indicated in ()
kDa
RMC2C mCherry
RMC2C SMARCB1
96hr
24hr
12hr
96hr
24hr
48hr
72hr
12hr
72hr
48hr
RMC2C mCherry
RMC2C SMARCB1
96hr
24hr
12hr
96hr
24hr
48hr
72hr
12hr
72hr
48hr
kDa
kDa
kDa
kDa
250 -
130 -
90 -
70 -
250 -
130 -
CDH1 (97kDa)
CST 3195
3min
90 -
70 -
70 -
TFCP2L1 (54 kDa)
Sigma HPA029708
2min
70 -
55 -
55 -
35 -
35 -
25 -
25 -
15 -
15 -
250 -
250 -
NFE2L2 (68kDa)
Abcam 62352
2min
140 -
140 -
90 -
90 -
70 -
70 -
70 -
MITF (58kDa)
Interchim MS-771-P
3 min
70 -
55 -
55 -
55 -
55 -
GPX4 (22kDa)
R&D 5457-SP
4min
35 -
35 -
MYC (58kDa)
SCT sc-40
2min
25 -
25 -
15 -
15 -
55 -
55 -
90 -
70 -
55 -
35 -
25 -
90 -
70 -
55 -
35 -
25 -
ACSL4 (79kDa)
ThermoFisher PA4-89830
1min
VIM (53kDa)
CST 5741
1min
55 -
55 -
35 -
35 -
25 -
25 -
FN1 (270kDa)
Sigma F3648
3min
250 -
250 -
140 -
140 -
90 -
90 -
70 -
70 -

## Slide 3
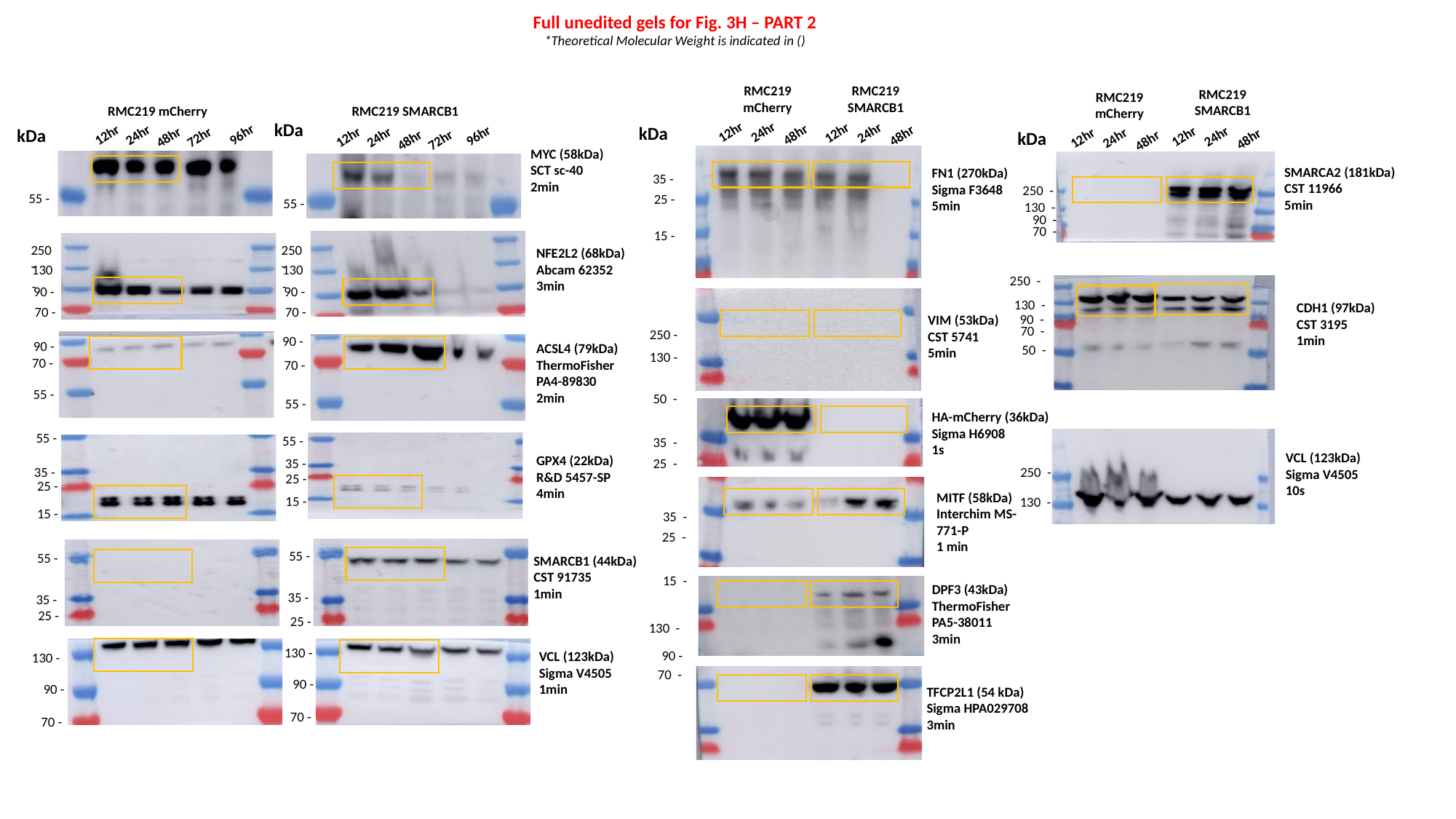

Full unedited gels for Fig. 3H – PART 2
*Theoretical Molecular Weight is indicated in ()
RMC219 mCherry
24hr
12hr
48hr
RMC219 SMARCB1
RMC219 SMARCB1
RMC219 mCherry
24hr
12hr
48hr
RMC219 mCherry
RMC219 SMARCB1
96hr
24hr
12hr
96hr
24hr
48hr
72hr
12hr
72hr
48hr
kDa
kDa
MYC (58kDa)
SCT sc-40
2min
55 -
55 -
250 -
250 -
NFE2L2 (68kDa)
Abcam 62352
3min
130 -
130 -
90 -
90 -
70 -
70 -
90 -
90 -
ACSL4 (79kDa)
ThermoFisher PA4-89830
2min
70 -
70 -
55 -
55 -
55 -
55 -
GPX4 (22kDa)
R&D 5457-SP
4min
35 -
35 -
25 -
25 -
15 -
15 -
55 -
55 -
SMARCB1 (44kDa)
CST 91735
1min
35 -
35 -
25 -
25 -
130 -
VCL (123kDa)
Sigma V4505
1min
130 -
90 -
90 -
70 -
70 -
24hr
12hr
48hr
24hr
12hr
48hr
kDa
kDa
SMARCA2 (181kDa)
CST 11966
5min
FN1 (270kDa)
Sigma F3648
5min
35 -
25 -
15 -
250 -
130 -
90 -
70 -
250 -
130 -
CDH1 (97kDa)
CST 3195
1min
90 -
VIM (53kDa)
CST 5741
5min
70 -
250 -
50 -
130 -
50 -
HA-mCherry (36kDa)
Sigma H6908
1s
35 -
VCL (123kDa)
Sigma V4505
10s
25 -
250 -
MITF (58kDa)
Interchim MS-771-P
1 min
130 -
35 -
25 -
15 -
DPF3 (43kDa)
ThermoFisher PA5-38011
3min
130 -
90 -
70 -
TFCP2L1 (54 kDa)
Sigma HPA029708
3min

## Slide 4
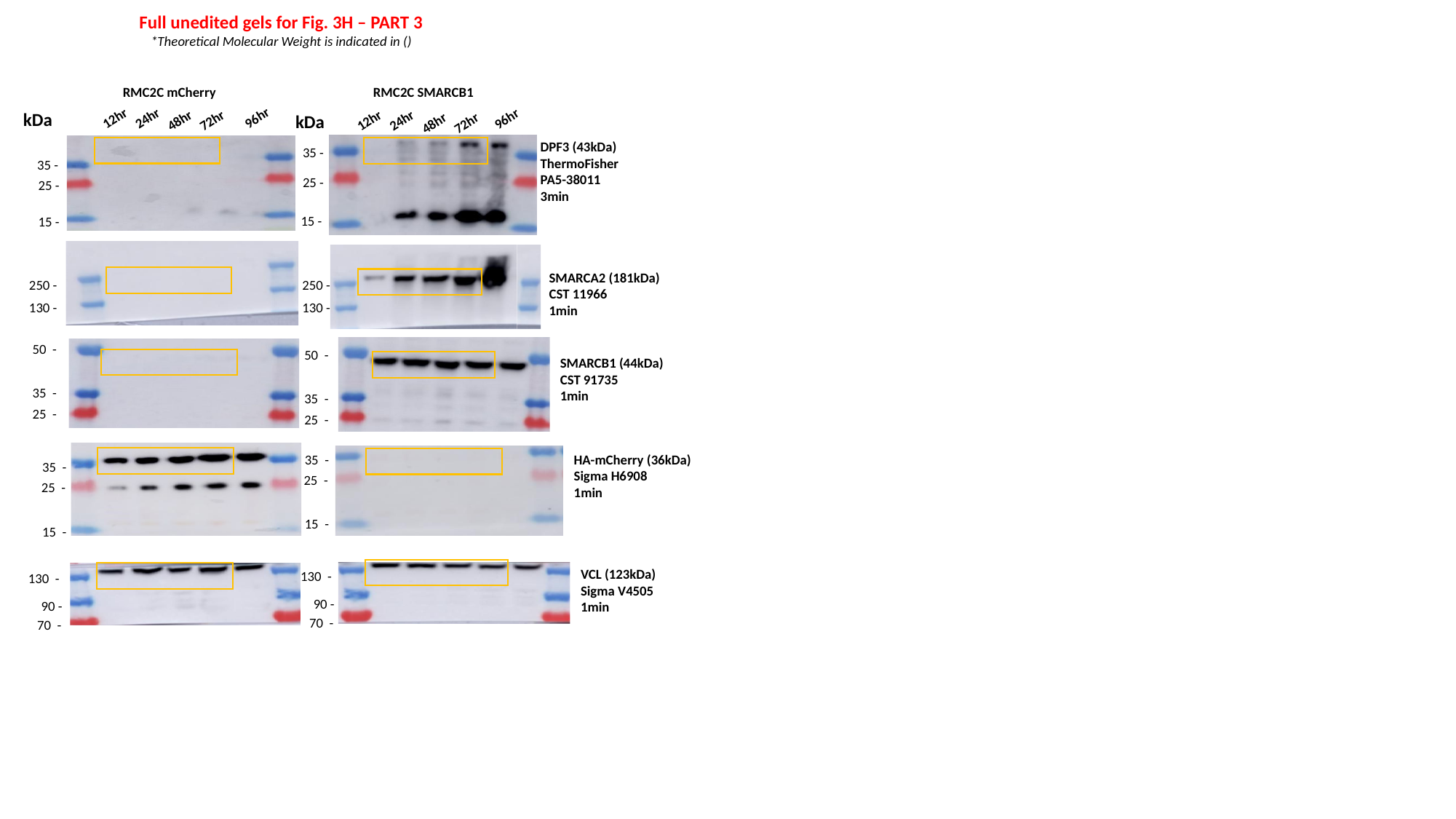

Full unedited gels for Fig. 3H – PART 3
*Theoretical Molecular Weight is indicated in ()
RMC2C mCherry
RMC2C SMARCB1
96hr
24hr
12hr
96hr
24hr
48hr
72hr
12hr
72hr
48hr
kDa
kDa
DPF3 (43kDa)
ThermoFisher PA5-38011
3min
35 -
35 -
25 -
25 -
15 -
15 -
SMARCA2 (181kDa)
CST 11966
1min
250 -
250 -
130 -
130 -
50 -
50 -
SMARCB1 (44kDa)
CST 91735
1min
35 -
35 -
25 -
25 -
35 -
HA-mCherry (36kDa)
Sigma H6908
1min
35 -
25 -
25 -
15 -
15 -
VCL (123kDa)
Sigma V4505
1min
130 -
130 -
90 -
90 -
70 -
70 -

## Slide 5
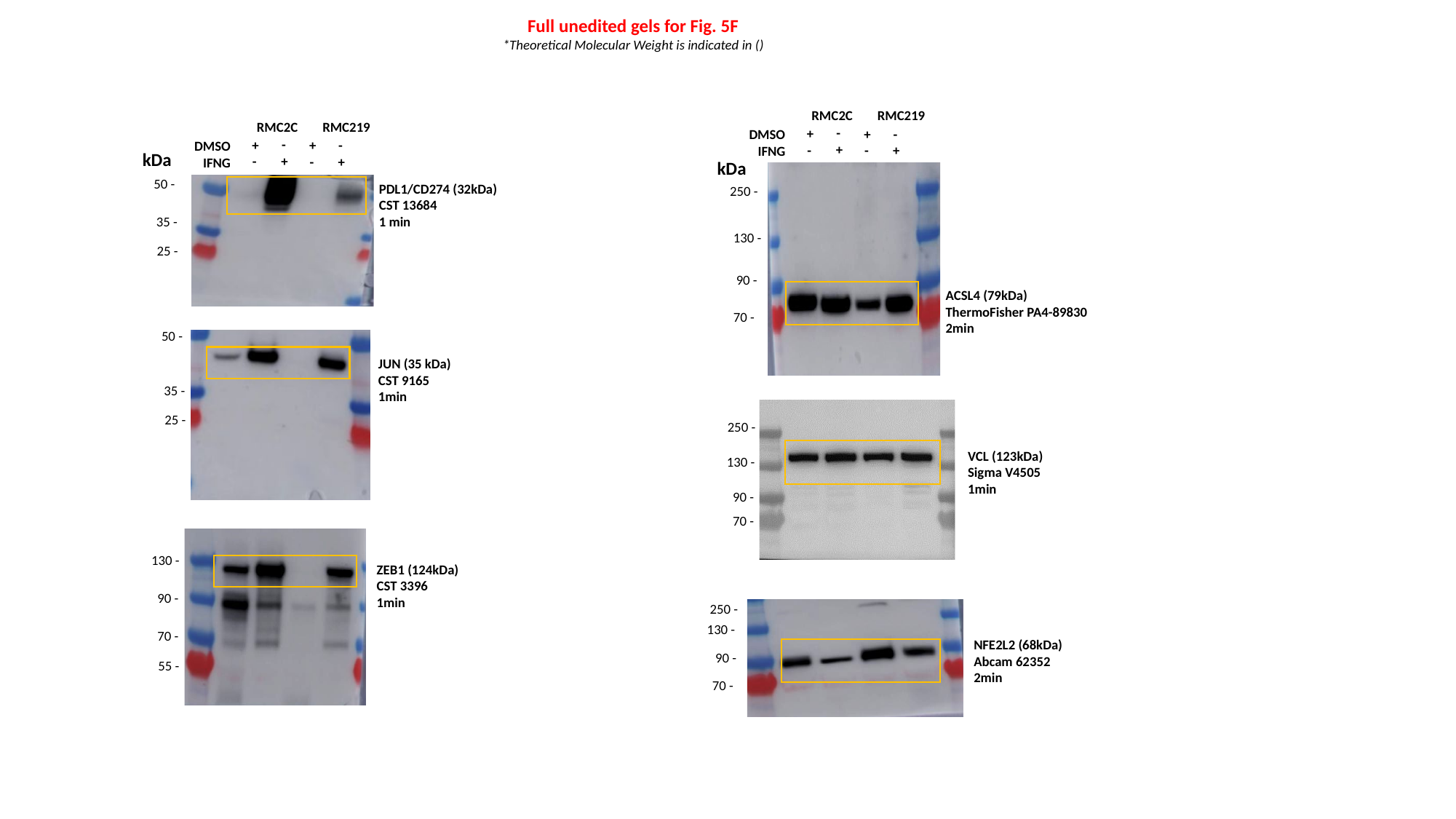

Full unedited gels for Fig. 5F
*Theoretical Molecular Weight is indicated in ()
RMC2C
RMC219
-
+
+
-
-
+
+
-
DMSO
IFNG
RMC2C
RMC219
-
+
+
-
-
+
+
-
DMSO
IFNG
kDa
kDa
250 -
130 -
90 -
ACSL4 (79kDa)
ThermoFisher PA4-89830
2min
70 -
50 -
PDL1/CD274 (32kDa)
CST 13684
1 min
35 -
25 -
50 -
JUN (35 kDa)
CST 9165
1min
35 -
25 -
250 -
VCL (123kDa)
Sigma V4505
1min
130 -
90 -
70 -
130 -
ZEB1 (124kDa)
CST 3396
1min
90 -
70 -
55 -
250 -
130 -
NFE2L2 (68kDa)
Abcam 62352
2min
90 -
70 -

## Slide 6
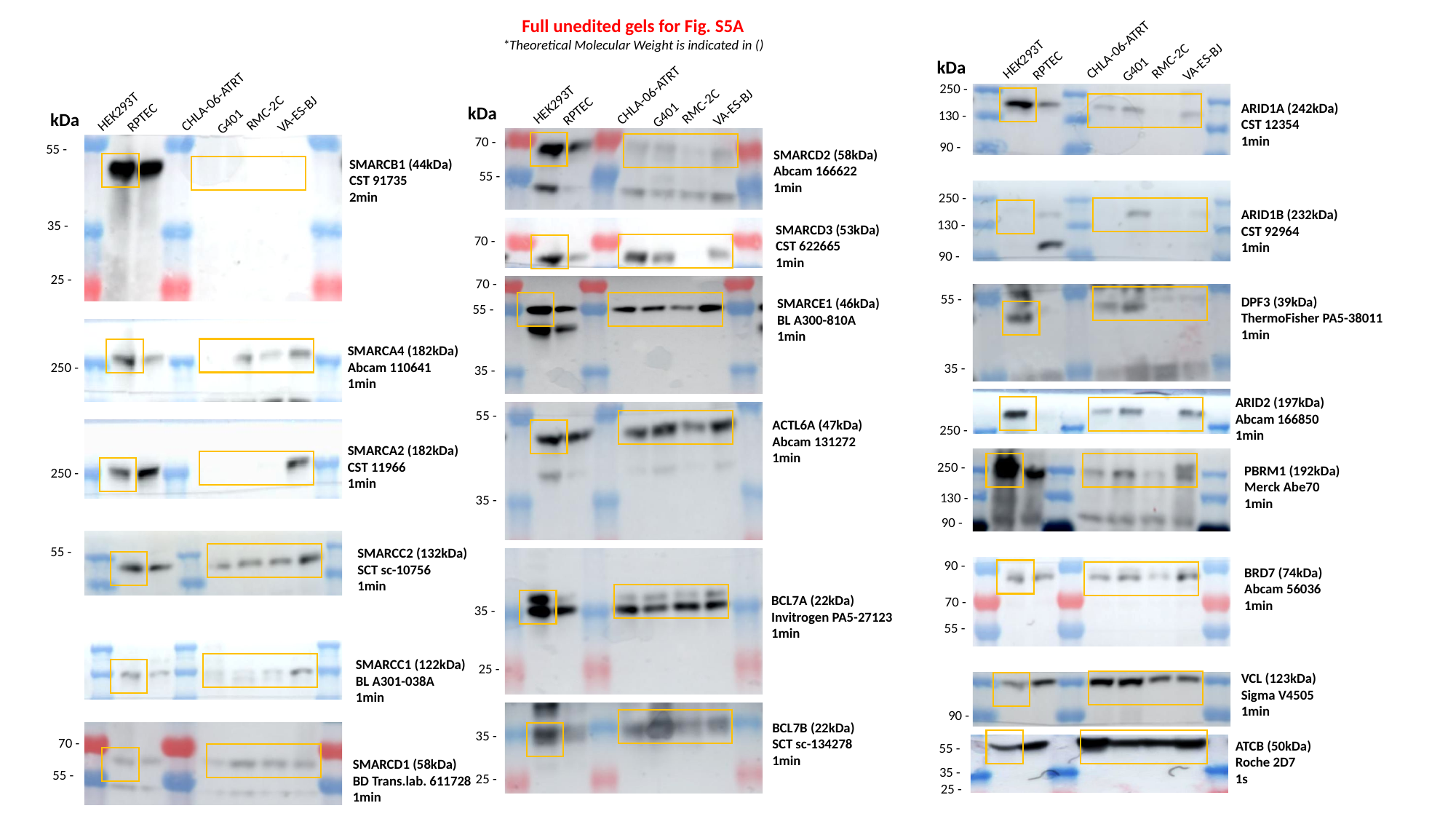

Full unedited gels for Fig. S5A
*Theoretical Molecular Weight is indicated in ()
CHLA-06-ATRT
HEK293T
RMC-2C
VA-ES-BJ
RPTEC
G401
kDa
250 -
CHLA-06-ATRT
HEK293T
RMC-2C
VA-ES-BJ
RPTEC
G401
CHLA-06-ATRT
HEK293T
RMC-2C
VA-ES-BJ
RPTEC
G401
ARID1A (242kDa)
CST 12354
1min
kDa
130 -
kDa
70 -
90 -
55 -
SMARCD2 (58kDa)
Abcam 166622
1min
SMARCB1 (44kDa)
CST 91735
2min
55 -
250 -
ARID1B (232kDa)
CST 92964
1min
130 -
35 -
SMARCD3 (53kDa)
CST 622665
1min
70 -
90 -
25 -
70 -
55 -
DPF3 (39kDa)
ThermoFisher PA5-38011
1min
SMARCE1 (46kDa)
BL A300-810A
1min
55 -
SMARCA4 (182kDa)
Abcam 110641
1min
250 -
35 -
35 -
ARID2 (197kDa)
Abcam 166850
1min
55 -
ACTL6A (47kDa)
Abcam 131272
1min
250 -
SMARCA2 (182kDa)
CST 11966
1min
250 -
PBRM1 (192kDa)
Merck Abe70
1min
250 -
130 -
35 -
90 -
55 -
SMARCC2 (132kDa)
SCT sc-10756
1min
90 -
BRD7 (74kDa)
Abcam 56036
1min
BCL7A (22kDa)
Invitrogen PA5-27123
1min
70 -
35 -
55 -
SMARCC1 (122kDa)
BL A301-038A
1min
25 -
VCL (123kDa)
Sigma V4505
1min
90 -
BCL7B (22kDa)
SCT sc-134278
1min
35 -
70 -
ATCB (50kDa)
Roche 2D7
1s
55 -
SMARCD1 (58kDa)
BD Trans.lab. 611728
1min
35 -
55 -
25 -
25 -

## Slide 7
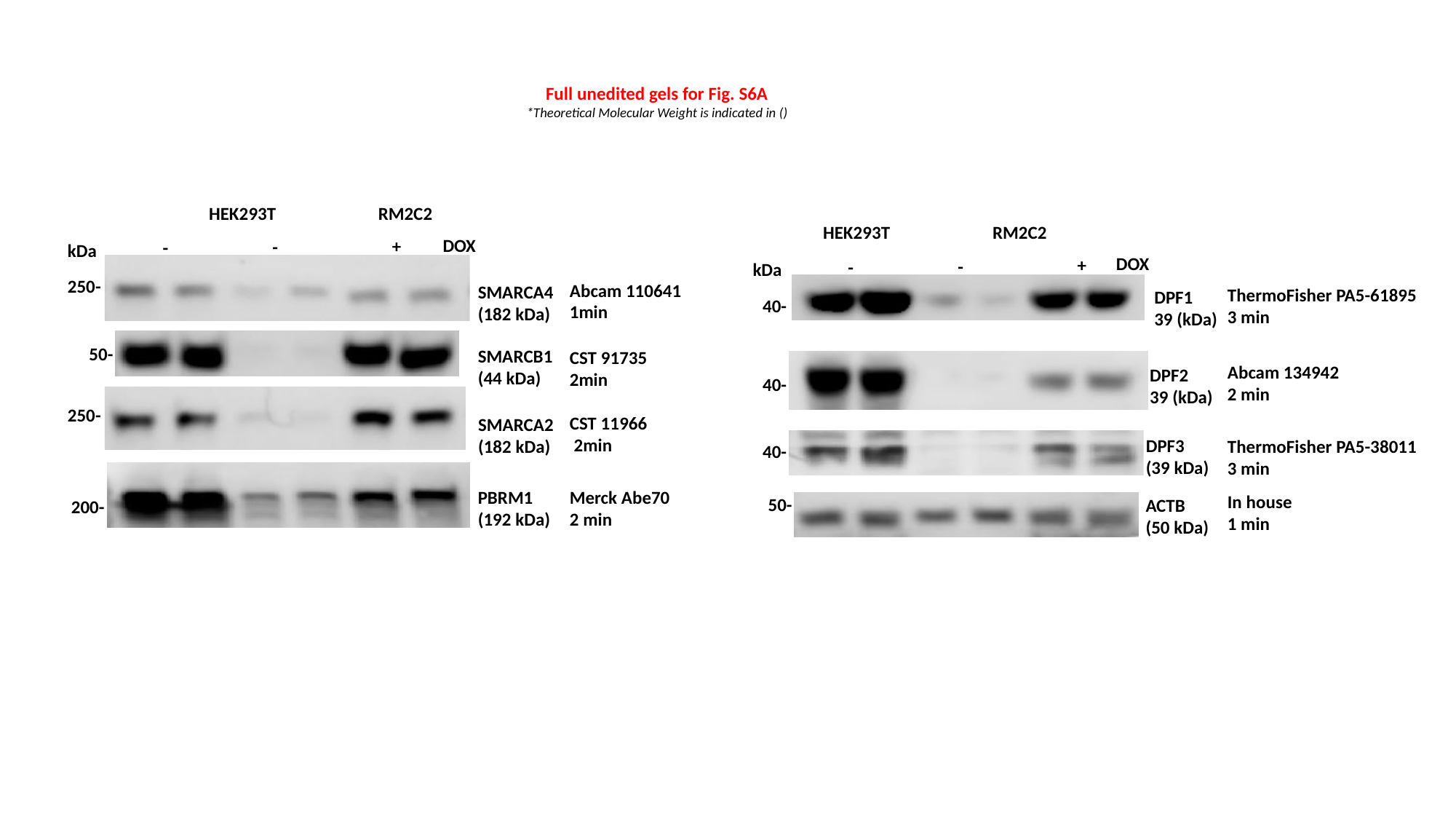

Full unedited gels for Fig. S6A
*Theoretical Molecular Weight is indicated in ()
RM2C2
HEK293T
RM2C2
HEK293T
DOX
+
-
-
kDa
DOX
+
-
-
kDa
250-
Abcam 110641
1min
SMARCA4
(182 kDa)
ThermoFisher PA5-61895
3 min
DPF1
39 (kDa)
40-
50-
SMARCB1
(44 kDa)
CST 91735
2min
Abcam 134942
2 min
DPF2
39 (kDa)
40-
250-
CST 11966
 2min
SMARCA2
(182 kDa)
DPF3
(39 kDa)
ThermoFisher PA5-38011
3 min
40-
PBRM1
(192 kDa)
Merck Abe70
2 min
In house
1 min
50-
ACTB
(50 kDa)
200-

## Slide 8
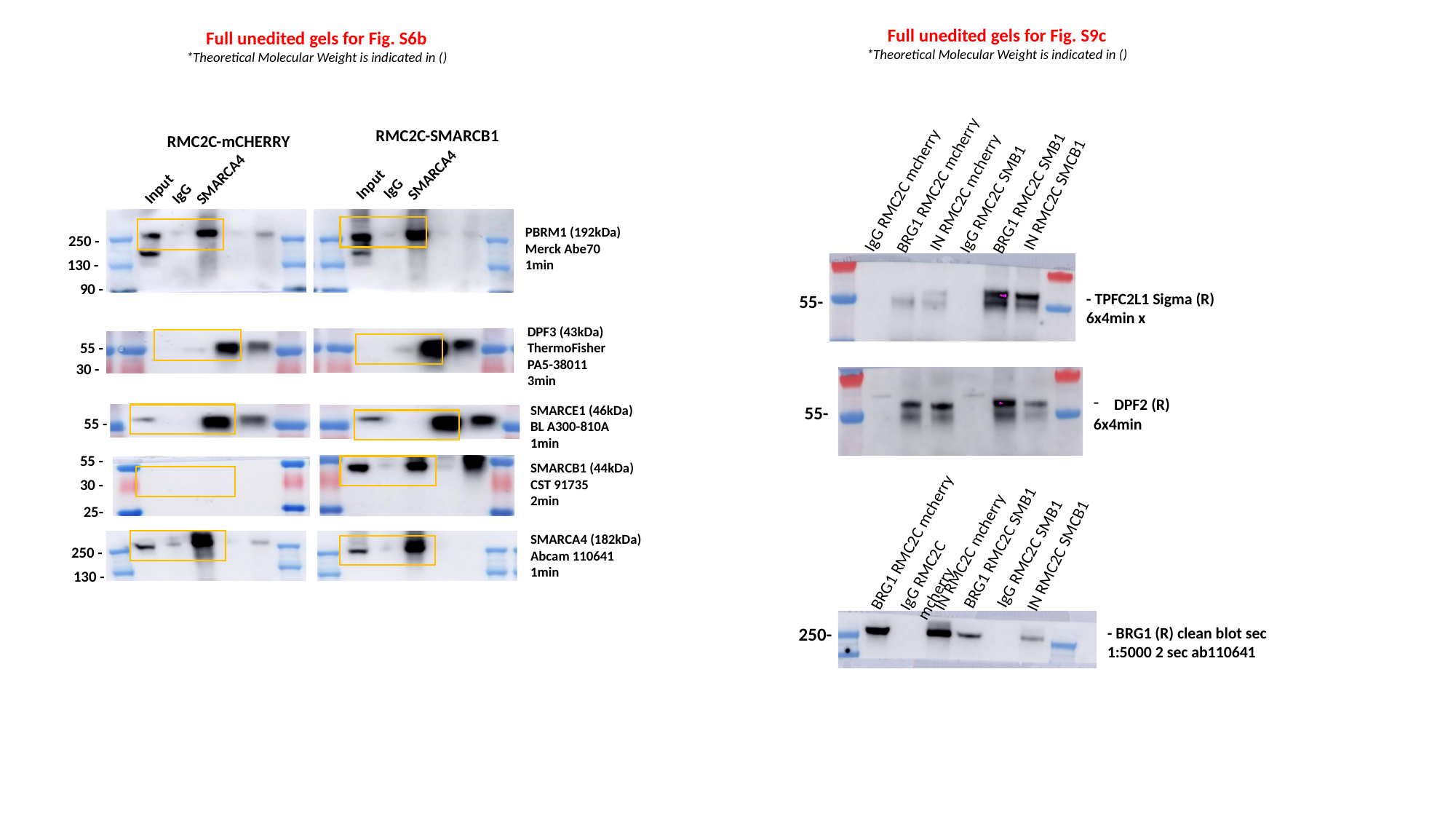

Full unedited gels for Fig. S9c
*Theoretical Molecular Weight is indicated in ()
Full unedited gels for Fig. S6b
*Theoretical Molecular Weight is indicated in ()
RMC2C-SMARCB1
RMC2C-mCHERRY
SMARCA4
SMARCA4
IgG
Input
BRG1 RMC2C mcherry
IgG
Input
IN RMC2C SMCB1
IN RMC2C mcherry
IgG RMC2C mcherry
IgG RMC2C SMB1
BRG1 RMC2C SMB1
PBRM1 (192kDa)
Merck Abe70
1min
250 -
130 -
90 -
- TPFC2L1 Sigma (R) 6x4min x
55-
DPF3 (43kDa)
ThermoFisher PA5-38011
3min
55 -
30 -
DPF2 (R)
6x4min
55-
SMARCE1 (46kDa)
BL A300-810A
1min
55 -
55 -
SMARCB1 (44kDa)
CST 91735
2min
30 -
25-
BRG1 RMC2C mcherry
SMARCA4 (182kDa)
Abcam 110641
1min
IN RMC2C SMCB1
IgG RMC2C SMB1
BRG1 RMC2C SMB1
IN RMC2C mcherry
IgG RMC2C mcherry
250 -
130 -
250-
- BRG1 (R) clean blot sec
1:5000 2 sec ab110641
